# Multisite Proton-Coupled Electron Transfer Enables Iodanyl Radical Catalysis

**DOI:** 10.1021/jacs.5c14648

**Published:** 2025-11-18

**Authors:** Phong Thai, Brandon L. Frey, Remy F. Lalisse, Lauv Patel, Poulami Mukherjee, Zhihui Song, Raanan Carmieli, Osvaldo Gutierrez, David C. Powers

**Affiliations:** † Department of Chemistry, 14736 Texas A&M University, College Station, Texas 77843, United States; ‡ Department of Chemistry and Biochemistry, University of California, Los Angeles, California 90095, United States; § Department of Chemistry and Biochemistry, University of Maryland, College Park, Maryland 20742, United States; ∥ Department of Chemical Research Support, 34976 Weizmann Institute of Science, Rehovot 7610001, Israel

## Abstract

The utility of hypervalent iodine reagents is often ascribed to the selective two-electron redox events that interconvert I­(I), I­(III), and I­(V) species during substrate oxidation. We recently reported 1,2-diiodoveratrole (**4a**) as an efficient catalyst for intramolecular oxidative C–H/N–H coupling and proposed that N–H activation was accomplished by an iodanyl radical (i.e., an I­(I)/I­(II) catalytic cycle) without accessing the corresponding I­(III) derivative. Transient iodanyl radicals have been proposed during reductive activation of I­(III) reagents, but the role of I­(II) intermediates in substrate activation is underexplored. Here, we report a combined experimental and computational investigation of N–H activation and C–N coupling promoted by iodanyl radicals. The assembled data indicate that anodically generated iodanyl radicals directly promote C–H/N–H coupling through a multisite proton-coupled electron transfer (MS-PCET) mechanism where the iodanyl radical serves as an electron acceptor and a carboxylate additive serves as a proton acceptor. Based on these mechanistic insights, two second-generation catalysts—1-iodo-4-methoxy-2-(trifluoromethyl)­benzene (**4c**) and 6,7-diiodo-1,1,4,4-tetramethyl-1,2,3,4-tetrahydronaphthalene (**4d**)—were developed. These catalysts display tailored redox properties that significantly expand the scope of both intra- and intermolecular metal-free electrocatalytic C–N bond-forming chemistry. Together, these results demonstrate that (1) iodanyl radicals can engage directly in substrate activation without the intermediacy of I­(III) species and (2) systematic variation of redox properties of iodanyl radicals enables rational catalyst optimization. The realization of one-electron hypervalent iodine mechanisms provides synthetic opportunities complementary to classical two-electron strategies and enables the development of new catalyst design concepts for metal-free electrocatalysis.

## Graphical abstract

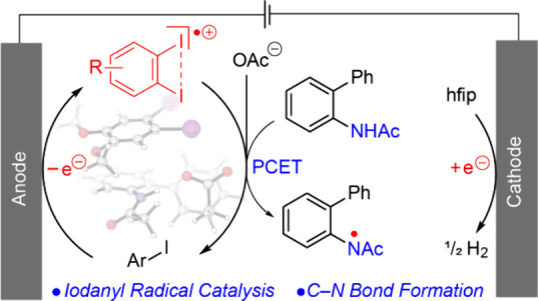

## Introduction

The past decade has witnessed incredible progress in the development of catalysis with heavy main group elements, including phosphorus, bismuth, and iodine.^1–8^ Like the late 4d- and 5d-transition metal ions that underpin many metal-catalyzed processes, heavy main group elements typically exhibit selective two-electron oxidation-reduction chemistry and engage in ligand exchange processes critical to coupling reactions.^9–12^ Unlike many transition metals, which display facile bidirectional oxidation and reduction by virtue of the closely spaced valence d-orbitals,^13^ heavy main group elements feature more widely spaced valence orbitals and thus typically engage in unidirectional redox reactions.^14,15^ As a result, while myriad efficient catalytic methods have been developed based on transition metal catalysts, heavy main group compounds are often utilized as stoichiometric reagents. The noted recent progress in heavy main group catalysis has been in large part enabled by the discovery of strategies—such as ligand-enforced geometrical distortion^16–19^ —that facilitate bidirectional redox chemistry at these elements.

Hypervalent iodine­(III)- and (V)-based compounds typically display unidirectional reduction chemistry (i.e., I­(III) to I­(I) or I­(V) to I­(III), respectively), which has been leveraged in a diverse array of oxidative substrate functionalization reactions (Figure 1).^9,20–23^ While progress has been made toward identifying protocols for hypervalent iodine catalysis,^24–30^ achieving selective oxidation of aryl iodides in preference to oxidatively labile substrates remains a significant challenge.^31–35^ The development of strategies to facilitate organoiodine oxidation while retaining (or expanding) the mechanistic diversity available to hypervalent iodine compounds would provide new opportunities in metal-free redox catalysis.

**Figure 1:**
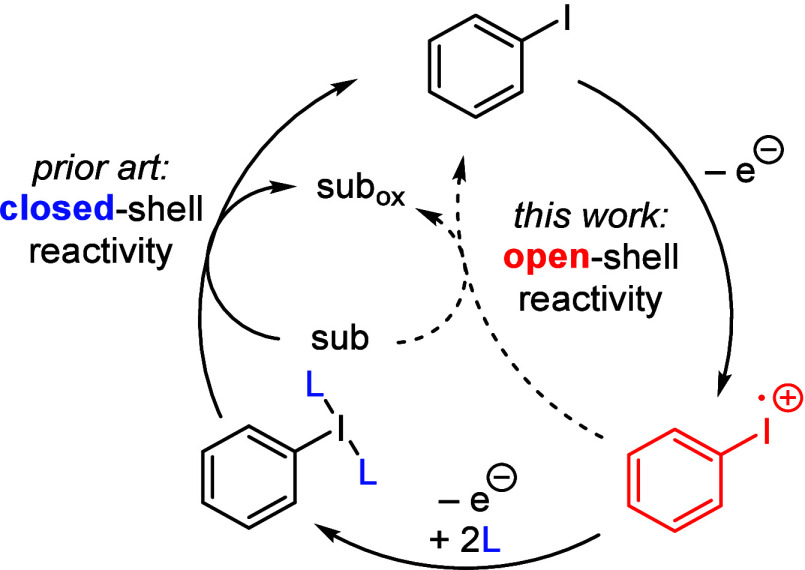
Hypervalent iodine reactivity via closed- or open-shell pathways.

During efforts to develop aerobic and electrochemical methods for the synthesis of hypervalent iodine compounds,^36–38^ we have become interested in the chemistry of aerobically or anodically generated iodanyl radicals (i.e., I­(II) species)^39,40^ as either intermediates en route to hypervalent iodine­(III) or as intermediates capable of direct substrate activation. The intermediacy of iodanyl radicals in synthesis was first proposed in the context of C–H halogenation reactions (Figure 2).^41^ Photolysis of I­(III) chloride **1** in the presence of hydrocarbon substrates afforded high levels of site selectivity, which was attributed to reactivity preferences of the corresponding iodanyl radical toward H atom abstraction (HAA) (Figure 2a). Maruoka and co-workers described similar structure-dependent selectivities in C–H oxygenation: Increased site selectivity and commensurate reduced reaction yield were observed for reactions that utilized bulkier aryl iodides (**3**, Figure 2b).^42^ These observations were interpreted as evidence of HAA by a transient iodanyl radical. In these reactions, I–X homolysis generates both an iodanyl radical and a ligand-based radical (i.e., X·) and differentiating which of these reactive species engages in substrate oxidation is challenging.^43–45^ A recent report by Xia et al. demonstrated a C–H bond functionalization protocol that was proposed to proceed via iodanyl radical intermediates (Figure 2c).^46^ Under these conditions, an iodanyl radical derived from Kita’s catalyst **2** trapped an alcohol to generate alkoxide-bound iodanyl radical **3**. Subsequent α-scission from this intermediate was proposed to generate a hexafluoroisopropoxy radical, which then promotes hydrogen-atom abstraction. Alternatively, HAT could proceed directly to iodanyl radical **3**. Despite these reports,^39–41,46–49^ the explicit role(s) of iodanyl radicals and the reaction mechanism(s) available to them have not received sufficient experimental scrutiny.

**Figure 2:**
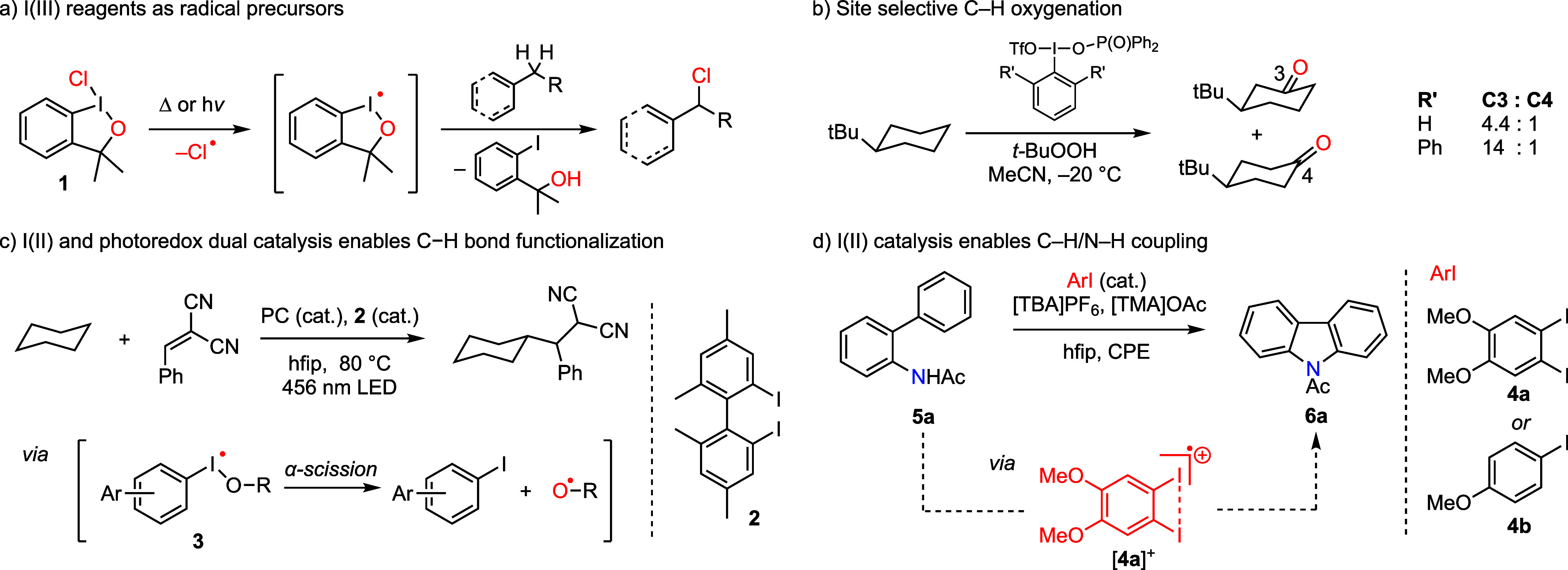
Summary of iodanyl radical chemistry. Thermal or photochemical activation of I–X bonds has been proposed to generate iodanyl radical intermediates. Based on substituent-dependent selectivities, the iodanyl radical, and not the ligand-centered radical, has been proposed to be directly involved in substrate activation in a) C–H chlorination and b) C–H oxygenation. c) Dual aryl iodide and photoredox catalysis promotes C–H bond functionalization (PC = 2,4,6-triphenylpyrylium tetrafluoroborate). d) Aryl iodide-catalyzed oxidative C–H/N–H coupling with **5a** affords heterocycle **6a**. These reactions have been proposed to proceed at iodanyl radical intermediates (i.e., [**4a**]^+^).

We recently identified 1,2-diiodoveratrole (**4a**) as an efficient (down to 0.5 mol %) electrocatalyst for intramolecular C–H/N–H coupling in the presence of carboxylate bases (Figure 2d).^50^ We developed electrocatalysis with **4a** based on the hypothesis that σ-delocalization between the proximal iodine substituents (i.e., oxidatively induced formation of a heavy σ-bond^51,52^ ) would facilitate iodine-centered oxidation. In comparison, 4-iodoanisole (**4b**), which cannot engage in delocalized iodine-centered oxidation is a much less efficient catalyst.^40^ Electrochemical data, *in situ* spectroscopic experiments, and crystallographic data of iodanyl radical [**4a**]^+^ suggested direct engagement of the iodanyl radical in substrate activation. Moreover, in the presence of added acetate, iodanyl radical [**4a**]^+^ promotes intramolecular C–H amination of **5a** to afford carbazole **6a**.^50^ *These preliminary findings not only raised the tantalizing prospect of heretofore unappreciated one-electron mechanisms in hypervalent iodine chemistry and catalysis but also provided the first well-defined venue to evaluate the elementary mechanistic steps available for substrate activation at an iodanyl radical*.

Here, we disclose the first detailed experimental and theoretical study of substrate activation at an iodanyl radical. The described experimental data, which is supported by modern quantum chemical calculations, indicate substrate activation via a multisite proton-coupled electron transfer (MS-PCET) mechanism in which the iodanyl radical serves as an electron acceptor and carboxylate additives serve as the proton acceptor (“multisite” PCET refers to a formal H-atom transfer in which the proton and electron go to two distinct acceptors^53^). These results provided the mechanistic basis for systematic catalyst optimization: Employing either more strongly oxidizing iodanyl radicals or more basic proton acceptors enabled the extension of iodanyl radical catalysis to C–N bond-forming reactions with previously inaccessible electron-deficient substrates and intermolecular C–N bond-forming reactions.

## Results and Discussion

The following discussion focuses on the chemistry of 1,2-diiodoveratrole (**4a**) and the corresponding iodanyl radical ([**4a**]^+^). We present 1) a detailed analysis of the speciation of iodanyl radical [**4a**]^+^ during catalysis, 2) the mechanism(s) by which [**4a**]^+^ engages in substrate functionalization, and 3) a catalyst optimization campaign that leveraged mechanistic insights to deliver significantly more active iodanyl radical catalysts. Analogous mechanistic experiments for 4-iodoanisole (**4b**) are described in the Supporting Information.

### Speciation of [**4a**]^+^ during Catalysis

During the development of C–H/N–H coupling under aryl iodide electrocatalysis, we observed that both aryl iodide catalyst and carboxylate base were required for efficient C–N bond construction (Figure 2d).^40,50^ To evaluate the speciation of [**4a**]^+^ in the presence of added carboxylates and substrates that feature N–H bonds, we carried out a series of electroanalytical and computational experiments. For the electrochemical experiments, we chose to utilize square wave voltammetry (SWV) experiments to avoid the capacitive background current that obscures some of the critical features described here during analogous cyclic voltammetry (CV) experiments (for corresponding CV analysis, see Figure S1).^54^ Computational details and optimized coordinates can be found in the Supporting Information. In these calculations, 2-methyl-1-propanol solvation was used due to the similar dielectric constant as compared to hfip.^55,56^ To account for the strong H-bonding interaction of acetate with hfip,^57^ calculations were carried out with explicitly hfip-solvated acetate.

#### Acetate Binding

We carried out a series of SWV experiments to evaluate the effect of carboxylate additives on the electrochemistry of **4a**. SWV experiments were performed on hfip solutions of **4a** (5.0 mM) with a 0.10 M tetrabutylammonium hexafluorophosphate ([TBA]­PF_6_) supporting electrolyte. The SWV of **4a** displays one well-defined oxidation peak centered at 1.20 V vs. Fc^+^/Fc which we ascribed to the one-electron oxidation of **4a** to [**4a**]^+^ (Figure 3 (black line)); no further oxidative features are observed up to 1.8 V. Addition of tetramethylammonium acetate ([TMA]­OAc) did not significantly impact the oxidative peak centered at 1.20 V, suggesting added acetate does not affect the formation or consumption of [**4a**]^+^ on the electrochemical time scale.^58^ On the other hand, addition of [TMA]­OAc resulted in a concentration-dependent growth of a new peak centered at 1.66 V (Figure 3 (blue line)). The current associated with this acetate-dependent peak saturates at roughly 30 mM [TMA]­OAc, which corresponds to 6 equiv of acetate with respect to **4a**. We ascribe the feature at 1.66 V to the oxidation of an acetate-bound adduct of [**4a**]^+^ (i.e., oxidation of [**4a**]­OAc to [**4a**]­OAc_2_) and the observation that the current associated with this feature does not saturate until superstoichiometric acetate loading suggests an unfavorable equilibrium binding of carboxylate with iodanyl radical [**4a**]^+^ (*vide infra*). Computational analysis of [**4a**]­OAc_2_ suggested that potential acetate dissociation is unfavorable (Figure S2). Bulk electrolysis of **4a** was performed at 1.66 V vs. Fc^+^/Fc in the presence of acetate did not result in any observable I­(III) compounds, which suggests that [**4a**]­OAc_2_ may not be stable under the electrochemical conditions (*vide infra*).

**Figure 3:**
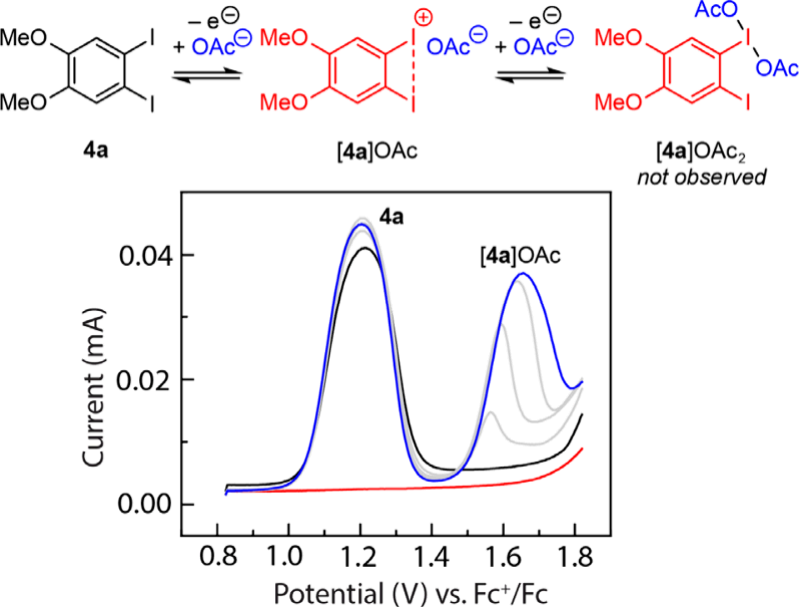
Electrochemical investigation of carboxylate binding to the iodanyl radical. SWV of a solution of **4a** (5.0 mM) in 0.10 M [TBA]­PF_6_/hfip varying [TMA]­OAc loading at 0.0 (black line), 7.2, 13.4, 22.0 (grey line), and 28.1 mM (blue line); SWV of a 35 mM solution of [TMA]­OAc (red line) in 0.10 M [TBA]­PF_6_/hfip. SWV conditions: glassy carbon working electrode, Pt counter electrode, and Ag^+^/Ag reference electrode and pulsed at 15 Hz with 25 mV amplitude and 4.0 mV increments. The SWV was externally referenced to Fc^+^/Fc.

Whereas the first oxidation feature (1.20 V vs. Fc^+^/Fc) is insensitive to the identity of the carboxylate additive; the potential of the second oxidation is carboxylate dependent. SWV of **4a** in the presence of [TMA]­OPiv, a stronger base than acetate, displays the carboxylate-insensitive feature at 1.20 V and the carboxylate-dependent feature at 1.60 V with a saturation point at approximately 4.5 equiv of pivalate (Figure S3a), suggesting that pivalate binding is more favorable and that the adduct, [**4a**]­OPiv, is easier to oxidize than [**4a**]­OAc. SWV of **4a** in the presence of [TMA]­TFA, a weaker base than acetate, also displays the carboxylate-insensitive feature at 1.20 V, but the carboxylate-dependent feature is at >1.8 V (Figure S3b), which suggests that [**4a**]­TFA does not easily undergo oxidation compared to [**4a**]­OAc and [**4a**]­OPiv. In the absence of aryl iodide, none of the tetramethylammonium carboxylates display significant electrochemical features in this potential window (Figure S4).^40^

Computationally, we evaluated the thermodynamics of binding of acetate to [**4a**]^**+**^ by considering three potential structures: A solvent-separated ion pair, [**4a**]­OAc, in which the acetate ion is engaged in H-bonding with hfip (i.e., [**4a**]^+^ and hfip–OAc^–^), a neutral acetate-stabilized I­(II) compound (i.e., **4a**–OAc; Figure 4a), and a contact ion pair, in which the acetate binds to the π face of [**4a**]^+^ (i.e., [**4a**]^+^···OAc^–^; Figure 4b). Initial structures were generated through manual conformational searching. All conformational isomers were optimized at the UB3LYP-D3/DGDZVP2-DGDZVP (I)-SMD (2-methyl-1-propanol) level, and the lowest-energy structures were used for subsequent calculations. The solvent-separated ion pair is the lowest energy formulation. Neutral iodanyl radical **4a**–OAc is 6.7 kcal/mol higher in energy and contact ion pair [**4a**]^+^···OAc^–^ is 3.2 kcal/mol higher in energy (for a noncovalent interaction plot (NCIPLOT)^59^ of [**4a**]^+^···OAc^–^, see Figure S5). Most of the energy cost associated with binding of acetate to [**4a**]^+^ originates from acetate desolvation from hfip (2.5 kcal/mol, Figure S6). The computed binding thermodynamics are consistent with the electroanalytical data, in which excess acetate was needed to saturate the current associated with one-electron oxidation of the iodanyl radical-carboxylate adduct. Further, consistent with the formulation as a solvent-separated ion pair without an explicit I–O interaction, the *in situ* EPR spectrum obtained during electrolysis of **4a** does not change significantly when measured in the presence of acetate (Figure S7).

**Figure 4:**
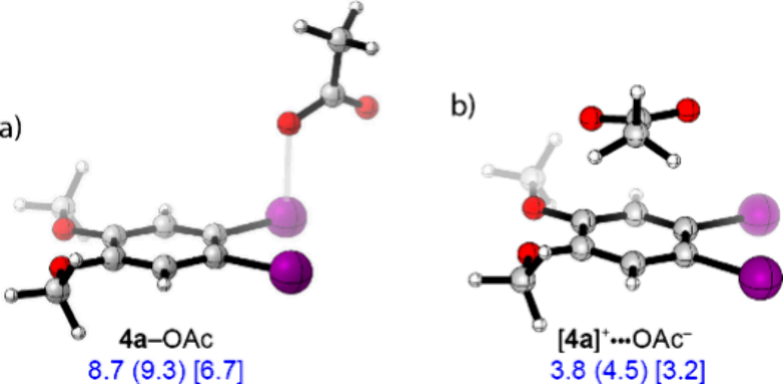
Computational analysis of acetate coordination to [**4a**]^+^ as a) a neutral acetate-stabilized I­(II) compound or b) a contact ion pair. The energies are referenced versus a solvent-separated ion pair of [**4a**]­OAc in which the acetate ion is engaged in H-bonding with hfip (i.e., [**4a**]^+^ and hfip–OAc^–^). Computations were carried out using the UB3LYP-D3/DGDZVP2-DGDZVP­(I)-SMD (2-methyl-1-propanol) level of theory, Δ*E* (Δ*H*) [Δ*G*].

#### Substrate Binding

SWV experiments were also carried out to evaluate the impact of biarylacetamide **5a** on the electrochemical behavior of **4a**. As was observed with added acetate, the oxidative peak was centered at 1.20 V vs. Fc^+^/Fc in the SWV of **4a** was not affected by the addition of **5a** (Figure S8). A second feature (1.72 V vs. Fc^+^/Fc) was observed to grow with increasing [**5a**]. We assign this feature to direct anodic oxidation of **5a**; the SWV of **5a** in the absence of **4a** also displays this feature. Computationally, association of **5a** to either **4a** or [**4a**]^**+**^ was calculated to be essentially thermoneutral (−0.3 and −2.0 kcal/mol, respectively; Figure S9). The non-covalent interaction (NCI) plot between **5a** and [**4a**]^+^ is provided in Figure S10. These results suggest there is no significant interaction of [**4a**]^+^ with **5a** and reaffirm the lack of substrate activation by [**4a**]^+^ in the absence of carboxylate additives.

### Mechanism of Substrate Activation

Figure 5 depicts the reaction mechanisms that we considered to be N–H activation by [**4a**]­OAc. Potential pathways include (a) disproportionation of the anodically generated iodanyl radical to afford I­(I) and I­(III) species with substrate activation at the resulting I­(III) species, (b) hydrogen-atom transfer (HAT) from substrate to the iodanyl radical to generate an iodine–hydride and a nitrogen-centered radical, (c) α-scission from alkoxide-bound iodanyl radical **8** to generate an alkoxide radical that engages in subsequent HAA, (d) electron transfer from acetate to [**4a**]^+^ to generate an acetoxy radical, which then engages substrate via HAA, and (e) MS-PCET between [**4a**]­OAc and **5a** in which [**4a**]^+^ serves as the electron acceptor and OAc^–^ serves as the proton acceptor.

**Figure 5:**
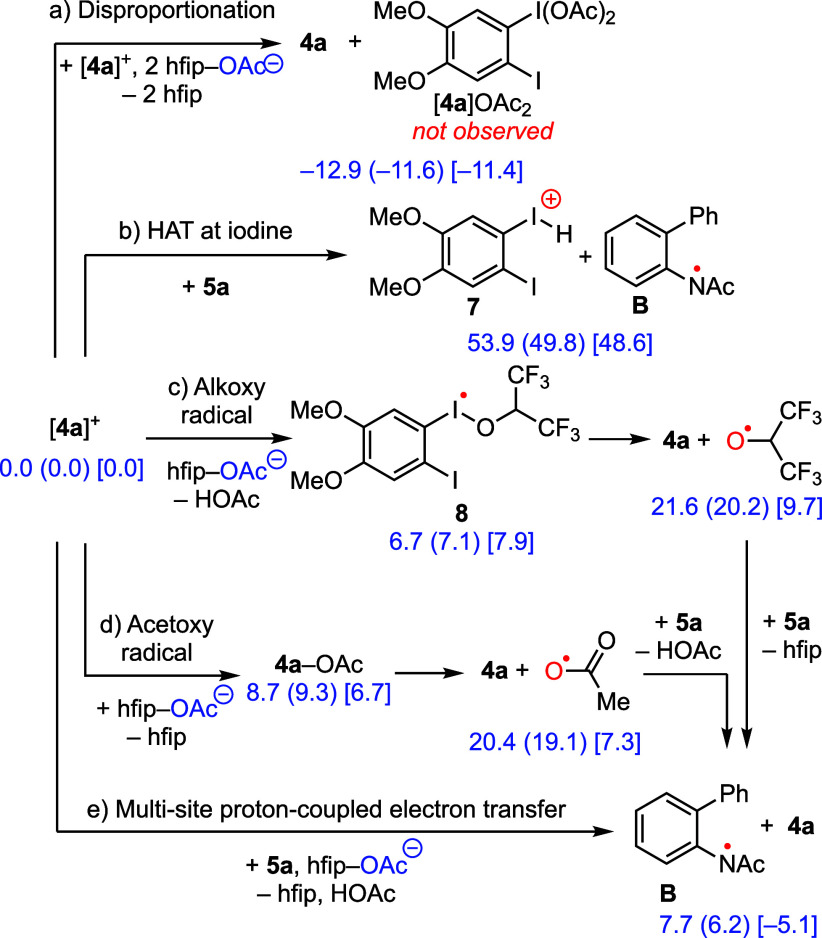
Reaction pathways for N–H activation at iodanyl radical [**4a**]^**+**^. Computations were carried out using UB3LYP-D3/DGDZVP2- DGDZVP­(I)-SMD (2-methyl-1-propanol) level of theory, Δ*E* (Δ*H*) [Δ*G*].

#### Iodanyl Radical Disproportionation

Disproportionation of [**4a**]^+^ would provide access to the I­(III) compound [**4a**]­OAc_2_ with concurrent generation of **4a** at the one-electron potential of **4a** (Figure 5, Path a). We previously observed such a bimolecular disproportionation pathway from a spectroscopically characterized *ortho*-*t*-butylsulfonyl iodanyl radical during the electrochemical synthesis of I­(III) reagents.^38^ In the case of [**4a**]^+^, bimolecular disproportionation to afford **4a** and I­(III) derivative [**4a**]­OAc_2_ is calculated to be downhill by −11.4 kcal/mol. Despite the favorable reaction thermodynamics, no I­(III) species are observed following bulk electrolysis of **4a**. Similarly, attempts to chemically prepare an I­(III) reagent by treatment with peracetic acid,^60^ *m*CPBA,^61^ O_2_/CH_3_CHO,^36^ or sodium perborate^62^ also failed to deliver I­(III) compounds. These observations are in contrast to the oxidation of 1,2-diiodobezene, which readily afford oxo-bridge diiodine­(III) compounds,^36^ and may indicate inherent instability of I­(III) derivatives of **4a**.

In addition to disproportionation, one could envision other pathways to generate the I­(III) derivatives. 2-fold oxidation of **4a** to generate an I­(III) intermediate is not viable because the second oxidation event observed at 1.66 V vs. Fc^+^/Fc in the SWV experiments (*vide supra*) is >400 mV above the potential relevant to catalysis with **4a** (i.e., 1.22 V vs. Fc^+^/Fc). Alternatively, Cariou et al. proposed oxidation of anodically generated iodanyl radicals by cathodically generated superoxide in their iodoarene-catalyzed spirocyclization of *N*-methoxyamides.^63^ We exclude this possibility because rigorous exclusion of oxygen from electrocatalytic cyclization of **5a** had no effect on the yield of carbazole, and thus, superoxide is not influencing the observed chemistry.

#### HAT to an Iodanyl Radical

HAA by [**4a**]^**+**^ to afford an iodine–hydride (i.e., ArI–H species) is an elementary step that is often invoked in the chemistry of reductively generated iodanyl radicals.^41,42,64,65^ Despite these proposals, there is no spectroscopic or structural characterization of any I–H compounds. We exclude such a pathway in our system based on the computed reaction energetics for HAA from **5a** to [**4a**]^**+**^ to afford **7** (Δ*G* = 48.6 kcal/mol, Figure 5, Path b).

#### N–H Activation by Alkoxy Radicals

Xia et al. reported that hexafluoroisopropoxy radical could be generated via the homolysis of an hfip-bound iodanyl radical.^46^ To evaluate the potential of an analogous pathway for the substrate activation of [**4a**]^+^, we examined an analogous pathway via intermediate **8** (Figure 5, path c). The formation of complex **8** was calculated to be uphill by 7.9 kcal/mol. Subsequent homolysis of the I–O bond to generate **4a** and an alkoxy radical is slightly further uphill (9.7 kcal/mol). HAT from the N–H valence of **5a** to the alkoxy radical was calculated to be favorable (Δ*G* = −5.1 kcal/mol). An alternate pathway involving an HAT step from **5a** to **8** was excluded due to high energetics (Δ*G* = 54.4 kcal/mol, Figure S11).^46^ Experimentally, [**4a**]^+^ was shown to be persistent in hfip at room temperature for several days,^50^ suggesting that hfip oxidation by [**4a**]^+^ is not rapid. Finally, electrolysis of an hfip solution of **5a** and **4a** in the absence of acetate afforded only trace C–N coupling product, suggesting that direct substrate activation by [**4a**]^+^ was unlikely.

#### N–H Activation by Acetoxy Radicals

A number of processes could be envisioned to give rise to acetoxy radicals, which could then engage in HAA with **5a** to generate aminyl radical **B** and acetic acid (Figure 5, Path d). We exclude direct interfacial oxidation (i.e., Kolbe electrochemistry) due to the lack of observed electrochemistry of TMA­[OAc] at potentials relevant to catalysis with **4a** (Figure 3 (red line)). Previous studies from our laboratory have also ascribed the redox innocence of acetate during catalysis to strong H-bonding between acetate and hfip, which inhibits acetate oxidation.^40^ Meanwhile, the formation of neutral acetate-stabilized I­(II) compound **4a**–OAc (Δ*G* = 6.7 kcal/mol) followed by homolysis could be envisioned to give rise to an acetoxy radical. Alternately, the acetoxy radical could result from direct electron transfer from acetate to [**4a**]^**+**^. While both processes are unfavorable (Δ*G* = 7.3 kcal/mol from [**4a**]^+^ and hfip–OAc^–^), subsequent HAT from the N–H valence of **5a** to the acetoxy radical is downhill (Δ*G* = −5.1 kcal/mol).

Experimentally, the decarboxylation products that are characteristic of acetoxy radicals (i.e., CO_2_, methane, and ethane) are not observed by gas chromatography (GC) analysis of the headspace following electrolysis of **4a** with [TMA]­OAc (4.0 equiv) in the absence of **5a** (Figure S12). In addition, diacetyl peroxide, which could be envisioned to form via the dimerization of acetoxy radicals, is also not observed (Figure S13). Together, these observations are inconsistent with carboxylate-centered redox activity and the intermediacy of acetoxy radicals during catalysis.

#### Multisite Proton-Coupled Electron Transfer

Lastly, we considered a MS-PCET mechanism in which [**4a**]^+^ serves as an electron acceptor and the acetate functions as the proton acceptor (Figure 5, Path e, and Figure 6). A ternary complex (**A**) of [**4a**]^+^, **5a**, and OAc^–^ assembled by H-bonding and weak noncovalent interactions (see Figure S14 for an NCI plot) was found at 4.6 kcal/mol above the constituents (Figure 6a). MS-PCET within complex **A** proceeds via a barrierless process from **A** via **TS**_**AB**_ (Figure 7, *vide infra*) and results in aminyl radical **B** and acetic acid (−5.1 kcal/mol). Consistent with electron transfer from **5a** to [**4a**]^+^, during the conversion from **A** to **TS**_**AB**_, the Mulliken charge located on iodine atoms decreases from 0.0 and +0.3 to −0.1 and −0.1, respectively (Figure S15), and the spin density shifts from iodine (0.5 → 0.0) to nitrogen (0.0 → 0.3) (Figure S16).

**Figure 6:**
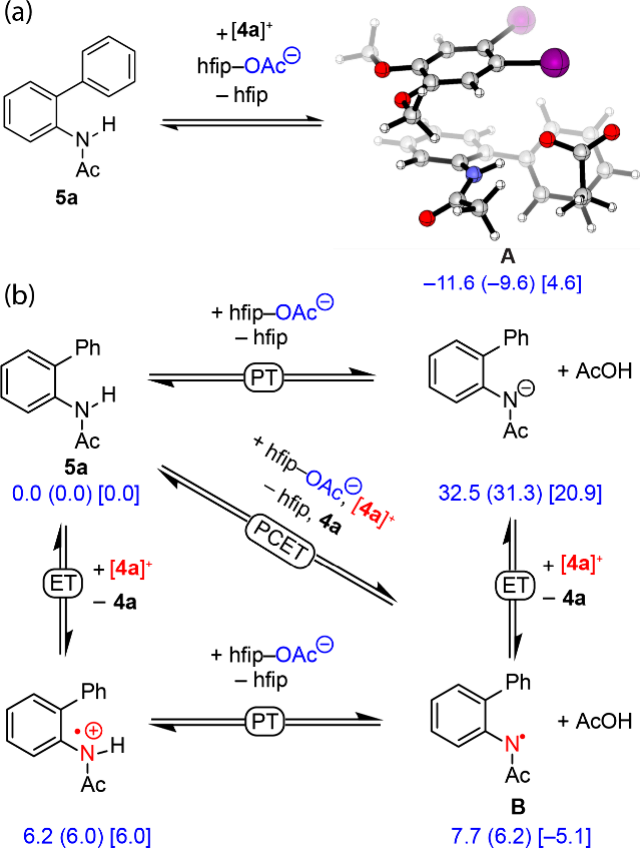
(a) MS-PCET proceeds within the ternary complex assembled from [**4a**]^**+**^, **5a**, and OAc^–^. (b) Square scheme for N–H bond activation using the iodanyl radical [**4a**]^**+**^ as the oxidant and acetate as the base. Computations were carried out using UB3LYP-D3/DGDZVP2- DGDZVP­(I)-SMD (2-methyl-1-propanol) level of theory, Δ*E* (Δ*H*) [Δ*G*].

**Figure 7:**
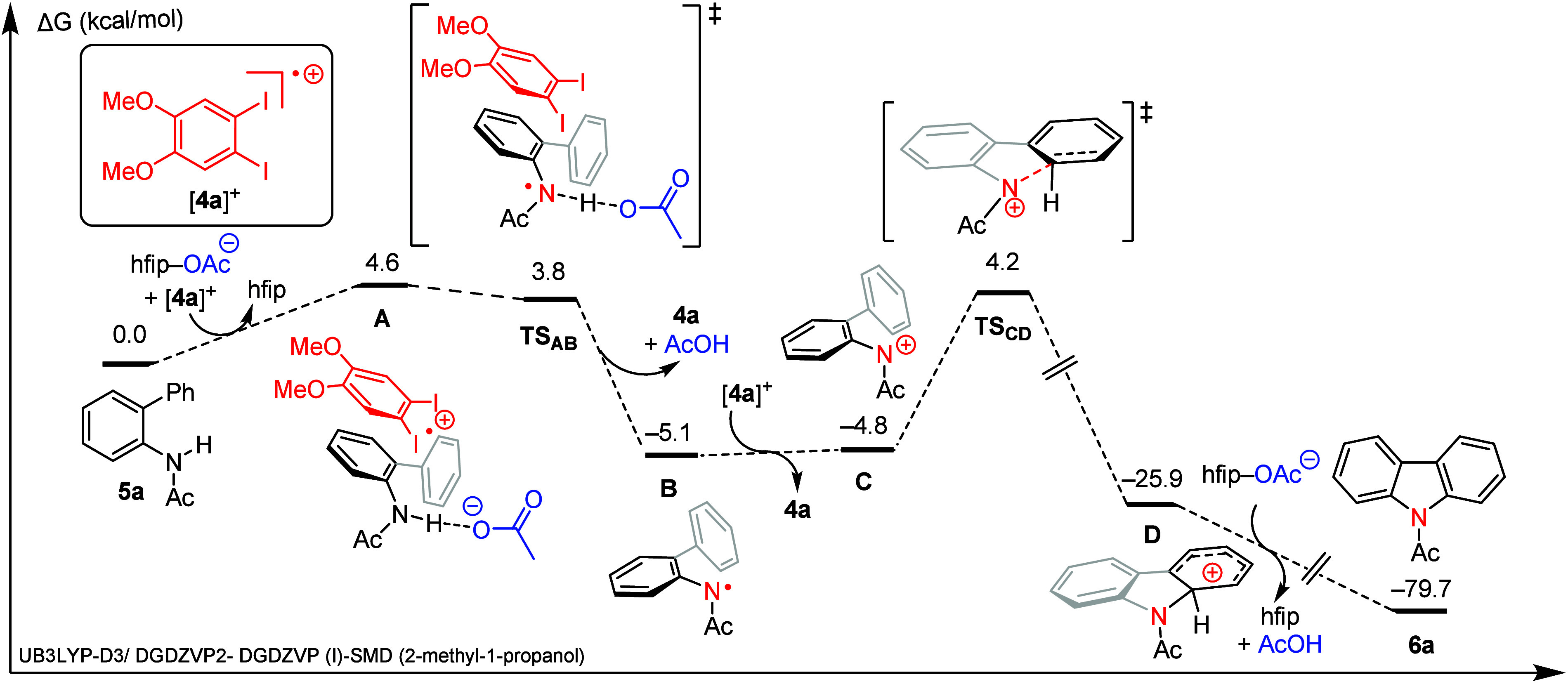
Reaction coordinate diagram of electrochemical catalyzed C–H/N–H coupling of **5a** by iodanyl radical [**4a**]^**+**^ and acetate. Computations were carried out using the UB3LYP-D3/DGDZVP2-DGDZVP (I)-SMD (2-methyl-1-propanol) level of theory, Δ*G*; Δ*E* and Δ*H* were omitted for clarity.

A sequential ET-PT pathway proceeds through an intermediate uphill by 6.0 kcal/mol, and a sequential PT-ET mechanism proceeds through an intermediate uphill by 20.9 kcal/mol (Figure 6b). Further, while MS-PCET proceeds from **A** (4.6 kcal/mol) without a barrier (TS_AB_ = 3.8 kcal/mol), the ET step of the ET-PT process confronts a barrier of 6.4 kcal/mol (calculated using Nelson’s four-point method^66^). The higher calculated barrier for the ET step in the ET-PT pathway compared with MS-PCET suggests that either both pathways may be accessible or the PCET pathway is asynchronous. The implications of this mechanism of catalyst design are discussed in detail in the “Impact on Synthetic Chemistry” Section below.

### Mechanism of C–N Bond Formation

Following initial N–H activation of **5a** via MS-PCET with [**4a**]­OAc, cyclization of radical **B** to afford carbazole **6a** requires the removal of a second H-atom equivalent. Figure 7 summarizes the results of the computational evaluation of the conversion of **B** to **6a**. The low-energy pathway identified in these studies proceeds via initial electron transfer from **B** to [**4a**]^+^ to afford the aminium cation **C**. Cyclization of **C** proceeds via **TS**_**CD**_ (via a barrier of 9.0 kcal/mol) to generate carbocation **D** (downhill by 21.1 kcal/mol). Subsequent deprotonation of **D** by acetate was computed to be highly exothermic at −53.8 kcal/mol to product **6a** with no discrete transition state. Overall, the pathway for C–N bond construction proceeds through an *N*-centered radical (**B**) that is analogous to intermediates previously proposed in the intramolecular cyclization of **5a**.^67^ Further, these results support the viability of one-electron pathway, which is complementary to reported open-shell mechanisms utilizing I­(III) reagents in hypervalent iodine-mediated C–N coupling.^68–70^

### Impact on Synthetic Chemistry

We sought to challenge the working hypothesis of N–H activation via an MS-PCET mechanism through catalyst derivatization studies. We hypothesized that if an MS-PCET reaction is operative, both the reduction potential of the iodanyl radical and the basicity of the carboxylate additive should have a dramatic impact on the efficiency of electrocatalytic C–N coupling. Here, we present the development of two new iodanyl radical catalysts, **4c** and **4d**. Pairwise manipulation of the iodanyl radical reduction potential and the basicity of the carboxylate additive enabled a substantial expansion of the generality and efficiency of iodanyl radical-catalyzed C–N coupling.

#### Catalyst Design

We envisioned that efficient MS-PCET processes could be applied to stronger N–H bonds if more strongly oxidizing iodanyl radicals were available. To evaluate this hypothesis, we prepared 1-iodo-4-methoxy-2-(trifluoromethyl)­benzene (**4c**) and 6,7-diiodo-1,1,4,4-tetramethyl-1,2,3,4-tetrahydronaphthalene (**4d**) (Figure 8). We hypothesized that [**4c**]^+^ would be a more strongly oxidizing iodanyl radical than [**4a**]^+^ due to the presence of a strongly electron-withdrawing −CF_3_ substituent. We speculated that weak I–F interactions between the fluorine atoms of the proximal −CF_3_ substituent and the formally I­(II) center could render [**4c**]^+^ persistent. We similarly envisioned that [**4d**]^+^ would be more oxidizing than [**4a**]^+^ because the strongly donating −OMe substituents of **4a** were replaced by less donating alkyl groups. In this case, we targeted dimethylation of the benzylic sites to avoid decomposition of the corresponding iodanyl radicals by activation of the benzylic C–H bonds, as we previously observed in 1,2-diiodo-4,5-dimethylbezene.^71^

**Figure 8:**
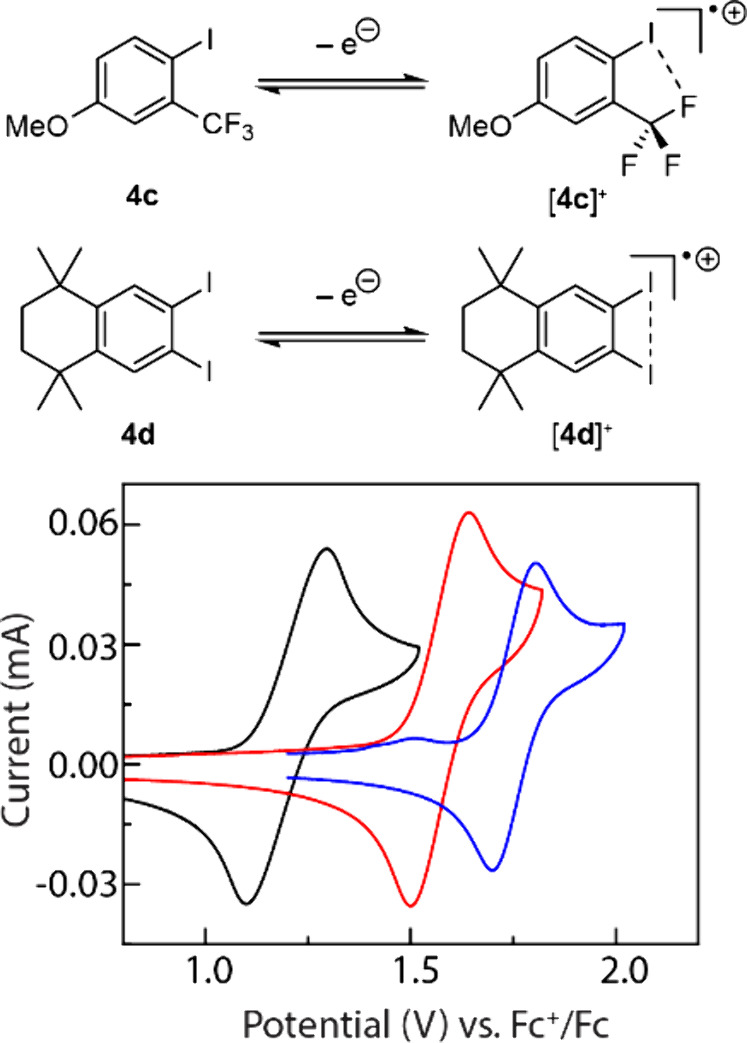
CVs of 5.0 mM solutions of **4a** (black line), **4c** (red line), and **4d** (blue line) in 0.10 M [TBA]­PF_6_/hfip. CV conditions: glassy carbon working electrode, Pt counter electrode, and Ag^+^/Ag reference electrode and scanned anodically at 100 mV/s. The CVs were externally referenced to Fc^+^/Fc.

Consistent with these design considerations, the iodanyl radicals derived from **4c** and **4d** are both more oxidizing than those from **4a** (*E*_1/2_ = 1.13 V vs. Fc^+^/Fc; *I*_pc_/*I*_pa_ = 0.94, Figure 8 (black line)): CV analysis of **4c** reveals *E*_1/2_ = 1.58 V vs Fc^+^/Fc (*I*_pc_/*I*_pa_ = 1.00, Figure 8 (black line)) and CV analysis of **4d** reveals *E*_1/2_ = 1.75 V vs Fc^+^/Fc (*I*_pc_/*I*_pa_ = 0.95, Figure 8 (blue line)).

Using the chemical and electrochemical conditions developed for the synthesis of [**4a**]^+^, the iodanyl radical cations [**4c**]^+^ and [**4d**]^+^ were prepared. A UV-vis spectrum collected during electrolysis of **4c** revealed the growth of two low energy absorbances centered at 620 and 840 nm (Figure 9 (red line)). Similar measurements carried out during the electrolysis of **4d** revealed features centered at 670 and 750 nm (Figure 9 (blue line)). Observation of low energy transitions is consistent with spectral data previously obtained for [**4a**]^+^ (i.e., broad absorbance at 645 nm, Figure 9 (black line)) and is consistent with TD-DFT calculations of [**4c**]^+^ and [**4d**]^+^ (Figures S17–S18). Based on these calculations, we assign these low energy transitions as predominantly π to π* character with significant contributions from the iodine centers.

**Figure 9:**
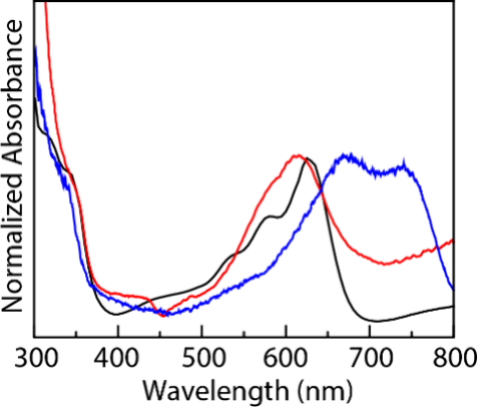
UV-vis spectra obtained during the electrolysis of solutions of 5.0 mM iodanyl radicals [**4a**]^**+**^ (black line), [**4c**]^**+**^ (red line), or [**4d**]^**+**^ (blue line) in 0.20 M [TBA]­PF_6_/hfip. Condition: Pt honeycomb dual working/counter electrode, and Ag^+^/Ag reference electrode; electrolysis was carried out at 1.20, 1.56, or 1.72 V vs. Fc^+^/Fc values for **4a**, **4c**, or **4d**, respectively. For **4d**, the solvent used was 2,2,2-trifluoroethanol and electrolysis was carried out at −30 °C.

#### Intramolecular C–N Coupling

Catalyst **4a** promotes efficient intramolecular C–N coupling with electron-rich and -neutral biarylacetamides: As examples, substrates **5a**, **5b**, and **5c** cyclize to the corresponding carbazoles in greater than 90% yield under the action of catalyst **4a** (Figure 10).^72^ In contrast, electron-deficient substrates such as **5d**–**5f**, represent challenges for catalysis with **4a**. The more oxidizing catalysts **4c** and **4d** display complementary substrate scope as compared with **4a**: These catalysts are poorly efficient for electron-rich and -neutral substrates due to unselective background oxidation of substrates at the one-electron potentials of **4c** and **4d** (Figure S19).^73^ In contrast, these catalysts efficiently promote the C–N coupling of electron-deficient substrates. Product **6d** bearing a *para*-ester group obtained in 92% and 72% yield when employing **4c** or **4d**, respectively, versus 20% yield when **4a** was used. Similarly, the more challenging substrates bearing a cyano or nitro group could be oxidized with catalyst **4c** in 83% (**6e**) and 72% (**6f**) yield, or with catalyst **4d** in 83% (**6e**) and 60% (**6f**) yield. In these cases, catalysis with **4a** delivered a trace amount of products (Figure S20).

**Figure 10:**
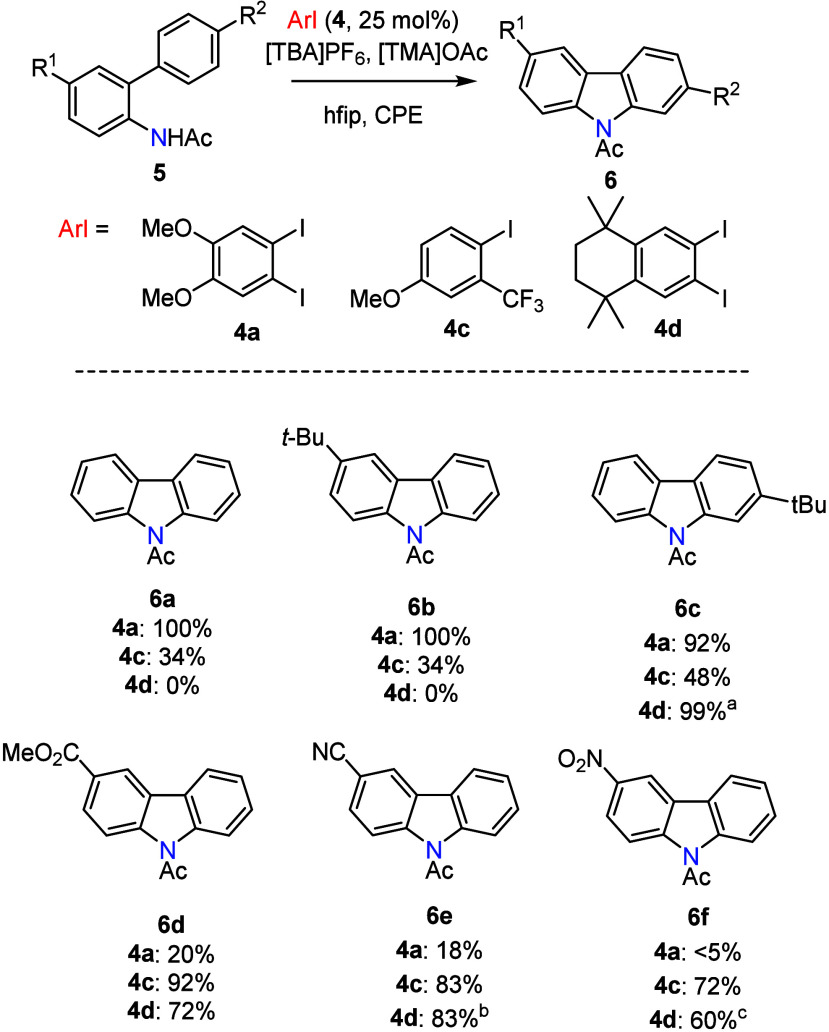
Intramolecular C–H/N–H coupling. Yields were determined by ^1^H NMR against a 1,3,5-trimethoxybenzene internal standard. Standard conditions: **5** (0.20 mmol, 40 mM in 5.0 mL hfip), [TMA]­OAc (2.0 equiv), **4** (25 mol %), [TBA]­PF_6_ (0.20 M), CPE at 1.20, 1.56, or 1.72 V vs. Fc^+^/Fc when using **4a**, **4c**, or **4d**, respectively. CPE for ∼50 C (2.6 F/mol). Undivided cells, glassy anodes, platinum cathodes, and Ag^+^/Ag reference. When using **4d**, the solvent was 0.8 mL:4.2 mL of CH_2_Cl_2_:hfip. ^a^Electrolysis at 1.72 V vs. Fc^+^/Fc in the absence of **4d** afforded similar yield; ^b^CPE was carried out for 120 C (6.2 F/mol); ^c^CPE was carried out for 250 C (13.0 F/mol).

#### Intermolecular C–N Coupling

Intermolecular C–N coupling of hydrazine derivatives with simple aromatic substrates has been demonstrated with hypervalent iodine reagents but represents a challenge for iodanyl radical catalysis.^74^ Catalyst **4a** is completely ineffective in promoting the amination of benzene with *N*-acetylephthalimide **9** using [TMA]­OAc as a base (Figure 11), regardless of catalyst loading. We attribute the lack of observed reactivity to the significant potential difference between **4a** (*E*_1/2_ = 1.20 V vs. Fc^+^/Fc) and **9** (*E*_1/2_ > 2.00 V vs. Fc^+^/Fc). The use of more strongly oxidizing iodanyl radical catalysts in combination with acetate enabled intermolecular C–N bond construction to be achieved, albeit in a modest yield. For example, at 10 mol % **4d**, carbazole **10a** is obtained in 48% yield. Changing the carboxylate additive from [TMA]­OAc to the more basic [TMA]­OPiv afforded product **10a** in 74% yield (10 mol % **4d**). Using these conditions (10 mol % **4d** and [TMA]­OPiv), various halogenated and dihalogenated substrates were productive to deliver amination products, with fluoro-, 1,2-diiodo-, and 1,3-dichlorobenzene affording products in 66% (**10b**), 62% (**10c**), and 40% (**10d**) (Figure S21). Product **10e** bearing a benzylic C–H bond at the arene ring was also tolerated to be delivered in 78% yield, while product **10f** bearing an ester and iodo group was obtained in 71% yield.

**Figure 11:**
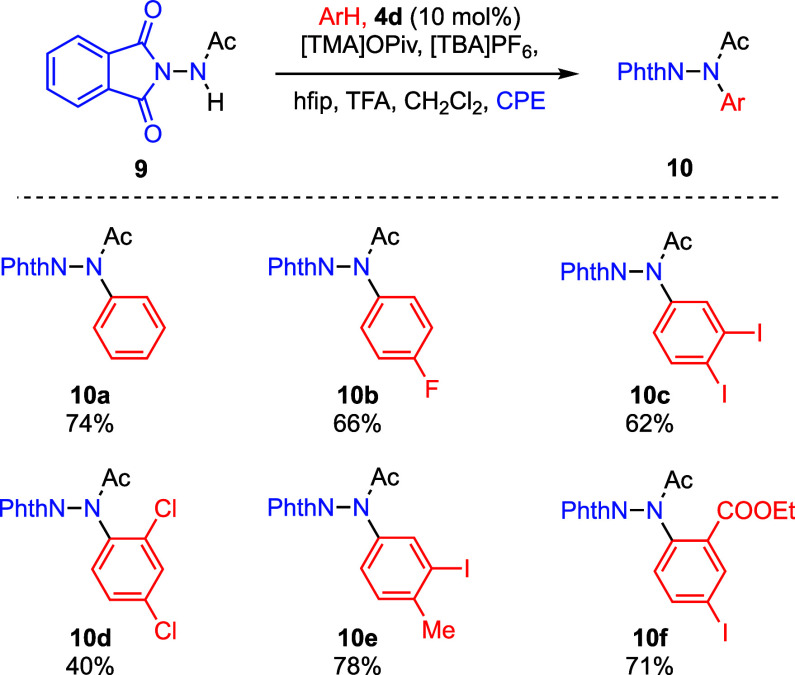
Intermolecular C–H/N–H coupling via iodanyl radical catalysis. Yields are NMR yields. Standard conditions: substrate **9** (0.20 mmol, 40 mM in hfip, 1.0 equiv), arene ArH (10.0 equiv), [TMA]­OPiv (2.0 equiv), TFA (2.0 equiv), CH_2_Cl_2_ (35 equiv), catalyst **4d** (10 mol %), [TBA]­PF_6_ (0.20 M), CPE at 1.72 vs Fc^+^/Fc for ∼80 C (4.1 F/mol), undivided cell, glassy carbon anode, platinum cathode, and Ag^+^/Ag reference. NPhth = phthalimide.

## Concluding Remarks

The development of robust platforms for metal-free electrocatalysis requires the development of catalysts that display facile bidirectional redox chemistry and well-defined substrate activation mechanisms. Hypervalent iodine reagents are a conceptually attractive platform for electrocatalysis because large families of aryl iodides with systematically tunable structures are available, and hypervalent iodine compounds have been demonstrated in a wide array of oxidative substrate functionalization reactions. Despite their promise, hypervalent iodine compounds are typically used as reagents, not catalysts, because the significant overpotentials needed to achieve two-electron oxidation of aryl iodides to the corresponding λ^3^-iodane derivatives (i.e., I­(III) compounds) are incompatible with oxidatively labile substrates.

Iodanyl radical catalysis, in which substrate activation is achieved at the one-electron potential of the aryl iodide, enables electrocatalysis by facilitating bidirectional redox chemistry and expanding the mechanisms available for substrate activation in hypervalent iodine chemistry. While iodanyl radicals have been sporadically proposed as reactive intermediates in stoichiometric reactions, these species are typically short-lived, and the reaction chemistry of iodanyl radicals has not been established. Recent progress toward iodanyl radical catalysis has been accelerated by the discovery of strategies to stabilize these open-shell species through delocalized I–I bonding, which has enabled iodanyl radical isolation, characterization, and evaluation of reactivity.

Here, we describe a detailed mechanistic investigation of N–H bond activation and C–N bond formation promoted by iodanyl radicals. The assembled data indicate that a MS-PCET mechanism is operative in which the iodanyl radical serves as an electron acceptor, while a carboxylate additive functions as the proton acceptor. Such a mechanism implies that both the reduction potential of the iodanyl radical intermediate and the basicity of the carboxylate additive afford independent opportunities for catalyst optimization. This concept has been demonstrated: Second-generation catalysts, designed to support more oxidizing iodanyl radical intermediates, in tandem with stronger carboxylate bases, expand the scope of C–N coupling to include electron deficient and intermolecular variants. Together, these results indicate the importance of catalyst design principles to elicit facile bidirectional redox chemistry, the importance of one-electron activation mechanisms in hypervalent iodine catalysis, and the potential to use iodanyl radicals as systematically tunable platforms for metal-free electrocatalysis.

## Supplementary Material



## Notes

Experimental procedures, electrochemical and spectral data, and Cartesian coordinates for the calculated structures (PDF)
