# Supplementary material for: Multisite Proton-Coupled Electron Transfer Enables Iodanyl Radical Catalysis
Source: J Am Chem Soc. 2025 Nov 18;147(48):44336–45. doi: 10.1021/jacs.5c14648 (PMC12679642; doi:10.1021/jacs.5c14648)
Supplement: Supplementary file 1 [file ja5c14648_si_001.pdf]

Supporting Information

Multi-Site Proton-Coupled Electron Transfer Enables Iodanyl Radical Catalysis

Phong Thai,[a,‡] Brandon L. Frey,[a,‡] Remy F. Lalisse,[b] Lauv Patel,[a] Poulami Mukherjee,[b] Zhihui Song,[c] Raanan Carmieli,[d] Osvaldo Gutierrez,[b,*] and David C. Powers[a,*]

[a] Department of Chemistry, Texas A&M University, College Station, Texas, 77843, United States
[b] Department of Chemistry and Biochemistry, University of California, Los Angeles, California, 90095, United States
[c] Department of Chemistry and Biochemistry, University of Maryland, College Park, Maryland, 20742, United States
[d] Department of Chemical Research Support, Weizmann Institute of Science, Rehovot, 7610001, Israel

‡These authors contributed equally to this work.

Email: o.gutierrez@g.ucla.edu, powers@chem.tamu.edu

Table of Contents

A. General Considerations S3
- A.1 Materials and Methods S3
- A.2 Characterization Details S3
- A.3 Electrochemical Details S4
- A.4 Computational Details S4

B. Synthesis and Characterization S5
- B.1 Synthesis of Starting Materials S5
- B.2 Intramolecular C–H Amination Electrocatalysis S8
- B.3 Intermolecular C–H Amination Electrocatalysis S9

C. Supporting Data S10

D. Mechanistic Data for 4-Iodoanisole Catalysis S31

E. Computational Data S44
- E.1 Optimized Coordinates for 1,2-Diiodoveratrole Catalysis S44
- E.2 Optimized Coordinates for 4-Iodoanisole Catalysis S62
- E.3 Optimized Coordinates of Miscellaneous Structures S79

F. Additional Data S98

G. NMR Spectra of New Compounds S103

H. References S110

A. General Considerations

A.1 Materials and Methods Tetrabutylammonium hexafluorophosphate ([TBA]PF_6) was purchased from Oakwood chemicals and recrystallized from ethanol. All other chemicals and solvents were obtained as ACS reagent grade and used as received. 1,1,1,3,3,3-Hexafluoro-2-isopropanol (hfip), 2,2,2-trifluoroethanol (tfe), and trifluoroacetic acid (TFA) were acquired from Oakwood Chemicals. Silica gel (0.060–0.200 mm, 60A for column chromatography) and 1,3-dichlorobenzene were obtained from Acros Organics. 4-Iodoanisole, 2-iodotoluene, acetic anhydride, tetrabutylammonium bromide ([TBA]Br), hexanes, ethyl acetate, dichloromethane (CH_2Cl_2), dimethylformamide (DMF), and benzene were purchased from Sigma Aldrich. Sodium persulfate ($Na_2S_2O_8$) and 2,5-dichloro-2,5-dimethylhexane were acquired from AmBeed. Anhydrous sodium sulfate, anhydrous sodium bicarbonate, and anhydrous potassium iodide were obtained from VWR. Pivalic acid, 2-bromoaniline, and (4-(methoxycarbonyl)phenyl)boronic acid were purchased from Tokyo Chemical Industry (TCI). Fluorobenzene, nitrobenzene, and tetramethylammonium hydroxide pentahydrate ([TMA]OH·$5H_2O$) were acquired from Beantown Chemical (BTC). Benzonitrile was obtained from EMD Millipore. Ethyl 3-iodobenzoate and 1,2-diiodobenzene and were purchased from Thermo Scientific Chemicals. Phenylboronic acid was acquired from Chem-Impex Int’l Inc. Palladium acetate ($Pd(OAc)_2$) and copper(I) iodide (CuI) were obtained from Strem Chemicals. (4-(Trifluoromethyl)phenyl)boronic acid was purchased from Matrix Scientific. Methanol and ethanol (200 proof) were acquired from Fischer Scientific. NMR solvents were purchased from Cambridge Isotope Laboratories and were used as received. All reactions were carried out under an ambient atmosphere unless otherwise noted. Tetramethylammonium acetate ([TMA]OAc),[1] tetramethylammonium pivalate ([TMA]OPiv),[2] 1,2-diiodo-4,5-dimethoxybenzene (**4a**),[3] 1-iodo-4-methoxy-2-(trifluoromethyl)benzene (**4c**),[4] *N*-(1,3-dioxoisoindolin-2-yl)acetamide (**9**),[5] diacetyl peroxide,[6] and 6,7-dibromo-1,2,3,4-tetrahydro-1,1,4,4-tetramethylnaphthalene (**S1**)[7] were prepared according to literature procedures. *N*-arylacetamide substrates **5a**–**5d**, **5f**, **S5a** were synthesized according to methods established in the literature.[8]

A.2 Characterization Details ^1H and ^{13}C NMR spectral acquisitions were recorded on an Inova 500 FT NMR (Varian), a VNMRS 500 FT NMR (Varian), or an Acsend™ 400 NMR (Bruker) and were referenced against residual proteo solvent signals: $CDCl_3$ (7.26 ppm, ^1H; 77.16 ppm, ^{13}C) and acetonitrile-d_3 (1.94 ppm, ^1H).[9] ^1H NMR data are reported as follows: chemical shift (δ, ppm), (multiplicity: s (singlet), d (doublet), t (triplet), m (multiplet), br (broad), integration). ^{13}C NMR data are reported as follows: chemical shift (δ, ppm). Mass spectrometry data was recorded on either Orbitrap Fusion™ Tribrid™ Mass Spectrometer or Q Exactive™ Focus Hybrid Quadrupole-Orbitrap™ Mass Spectrometer from ThermoFisher Scientific. An Agilent Trace 1300 GC with attached thermal conductivity detector and a custom-made 120 cm stainless steel column packed with Carbosieve-II was used for analysis of headspace gases. The column was kept at 200 °C and Ar was used as carrier gas. The detector was set to a temperature of 250 °C. Headspace gas (~300 μL) was transferred to the GC with a 0.50 mL Valco Precision Sampling Syringe (Series A-2) equipped with a Valco Precision sampling needle with a 5-point side port.

A.3 Electrochemical Details Cyclic voltammetry (CV), square wave voltammetry (SWV), and bulk electrolysis experiments were carried out using CH Instruments Electrochemical Analyzer (Model CHI620A or CHI620E) under ambient conditions unless specified otherwise. In all electrochemical experiments, a Ag^+/Ag reference solution was prepared using 0.10 M solution of $[TBA]PF_6$ in acetonitrile with 1.0 mM $AgNO_3$, and the applied voltage was corrected for IR compensation. CV and SWV experiments were carried out with a glassy carbon working electrode (circular surface, d = 3.0 mm), Pt counter electrode, and a Ag reference electrode (electrodes were purchased from CH Instruments). The working electrode was polished using 0.5 µm alumina in a figure eight motion prior to each measurement, and the distance between the working and counter electrode was 11.0 mm. CV and SWV scans starts at 0.80 V *vs.* Ag^+/Ag (0.62 V *vs.* Fc^+/Fc) and scans anodically at 23 °C; the voltametric plots are then externally referenced *vs.* Fc/Fc^+. For constant potential electrolysis (CPE), the Ag, glassy carbon, platinum (Pt), and graphite electrodes were purchased from IKA. All CPE experiments were carried out in a single-undivided 10-mL ElectraSyn glass vial (undivided cell) or in a Pro-Divide electrochemical cell (divided cell), both of which were purchased from IKA. In the undivided cell setup, the distance between the working and counter electrode was 6.0 mm; in the divided cell setup, the distance between the working and counter electrode was 18.0 mm. In both setups, the working and counter electrode each had a surface area of 312 mm^2.

A.4 Computational Details All ground state geometry optimizations were performed with Gaussian 16 Rev. C.01.[10] Open-shell ground state geometry optimizations were carried out with Density Functional Theory (DFT) calculations using the unrestricted B3LYP approximate density functional.[11, 12] The GD3 dispersion correction was used to simulate long-range dispersion interactions.[13-16] For all atoms except iodine the DGDZVP2 basis set was implemented and for iodine the DGDZVP basis set was used. These basis sets are commonly implemented for radical species.[17] Solvent effects were simulated in 2-methyl-1-propanol due to its similarity to hfip with the solvation model density (SMD) method with electrostatics in terms of the integral-equation formalism polarizable continuum model (IEF-PCM).[18-22] Optimized stationary points were confirmed to be local minima (all real vibrational frequencies) or transition states (one imaginary vibrational frequency) on the potential energy surface (PES) using vibrational frequency analysis. Each transition state was connected to its respective reactant and product by intrinsic reaction coordinate (IRC) analyses.[23-25] To evaluate the noncovalent interactions of the I(II) PCET reaction with [**4a**]$^+$ and [**4b**]$^+$, noncovalent interaction analysis (NCIPLOT) was conducted in which the reduced density gradient s (where s is a function of electron density *r* and its first derivative) is computed to show areas where noncovalent interactions are taking place.[26, 27]

B. Synthesis and Characterization

B.1 Synthesis of Starting Materials

Tetramethylammonium trifluoroacetate ([TMA]OTFA)

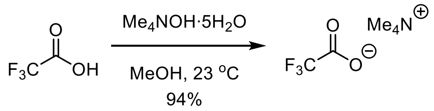

A 100-mL round-bottom flask was charged with trifluoroacetic acid (0.422 mL, 5.52 mmol, 1.00 equiv.), tetramethylammonium hydroxide pentahydrate ([TMA]OH· $5H_2O$, 1.01 g, 5.52 mol, 1.00 equiv.), and MeOH (5 mL). The mixture stirred vigorously for 10 min. The solvent was then removed *in vacuo* to afford the title compound as a white solid (973 mg, 94% yield). ^{1}H NMR (δ, 23 °C, 400 MHz, CD_3CN): 3.12 (s, 12H). ^{13}C NMR (δ, 23 °C, 100 MHz, CD_3OD): 162.2 (q, *J* = 35.1 Hz), 118.0 (q, *J* = 292.4 Hz), 55.9 (t, *J* = 4.1 Hz). ^{19}F NMR (δ, 23 °C, 376 MHz, CD_3CN): –75.9. HRMS-ESI: calculated for $[C_4H_{12}N]^+$ = 74.0964, observed $[C_4H_{12}N]^+$ = 74.0971; calculated for $[C_2F_3O]^-$ = 96.9901, observed $[C_2F_3O]^-$ = 112.9843.

6,7-Diiodo-1,1,4,4-tetramethyl-1,2,3,4-tetrahydronaphthalene (**4d**)

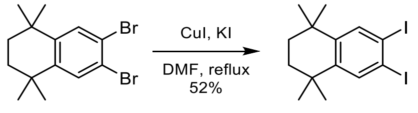

Under an N_2 atmosphere, a 250-mL round-bottom flask was charged with 6,7-dibromo-1,1,4,4-tetramethyl-1,2,3,4-tetrahydronaphthalene (**S1**, 202 mg, 0.584 mmol, 1.00 equiv.), potassium iodide (2.15 g, 12.7 mol, 22.0 equiv.), copper(I) iodide (2.21 g, 11.6 mol, 20.0 equiv.), and dry DMF (20 mL). The mixture was heated to reflux for 72 h. After cooling to 23 °C, the solvent was removed under reduced pressure. The residue was dissolved in CH_2Cl_2 (20 mL). To this solution was added an aqueous solution of $Na_2S_2O_8$ (1.0 M, 20 mL) and water (50 mL), and the mixture was stirred vigorously for 15 min. The organic layer was separated, dried over Na_2SO_4, and concentrated *in vacuo*. The residue was purified by SiO_2 chromatography (hexanes as eluent) to give the title compound as a white solid (130 mg, 52% yield). ^{1}H NMR (δ, 23 °C, 400 MHz, $CDCl_3$): 7.73 (s, 2H), 1.64 (s, 4H), 1.23 (s, 12H). ^{13}C NMR (δ, 23 °C, 100 MHz, $CDCl_3$): 147.3, 137.8, 104.2, 34.6, 34.1, 31.6. HRMS-APCI: calculated for $[C_{14}H_{18}I_2+H]^+$ = 439.9438, observed $[C_{14}H_{18}I_2+H]^+$ = 439.9484.

General Procedure for the Synthesis of *N*-arylacetamide Substrates

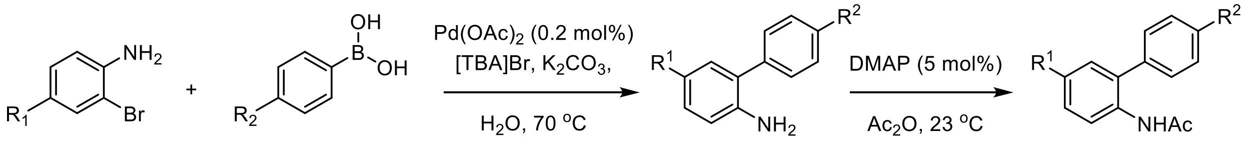

5

A 20-mL scintillation vial was charged with the appropriate 2-bromoaniline (5.60 mmol, 1.00 equiv.), the appropriate boronic acid (8.40 mmol, 1.50 equiv.), $Pd(OAc)_2$ (2.6 mg, 11 μmol, 0.20 mol%), [TBA]Br (1.80 g, 5.60 mmol, 1.00 equiv.), K_2CO_3 (1.93 g, 14.0 mmol, 2.50 equiv.), and H_2O (8.0 mL). A rubber septum was fitted onto the vial, and the reaction mixture was purged with N_2 gas for 10 min before being heated at 70 °C for 16 h. The mixture was cooled to 23 °C. The reaction mixture was extracted with EtOAc (3 × 30 mL). The combined organic fractions were combined, washed with 0.5 M NaOH (30 mL), washed with water (30 mL), dried over Na_2SO_4, and solvent was removed *in vacuo*. The obtained residue was used in the next step without further purification.

A 20-mL scintillation vial was charged with the residue from the previous step, 4-dimethylaminopyridine (DMAP, 34.2 mg, 0.280 mmol, 5.00 mol%), and acetic anhydride (5.0 mL). The reaction mixture was stirred at 23 °C for 16 h, after which 30 mL of EtOAc was added. The mixture was then transferred into a separatory funnel, washed with saturated $NaHCO_3$ aqueous solution until the evolution of CO_2 ceased. The layers were then separated. The organic fraction was dried over Na_2SO_4, and solvent was removed *in vacuo*. The residue was purified by SiO_2 chromatography (eluent 60:40 hexanes:ethyl acetate) to afford *N*-arylacetamides listed below.

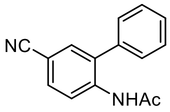

5e

N-(5-cyano-[1,1'-biphenyl]-2-yl)acetamide (**5e**). Prepared from 4-amino-3-bromobenzonitrile and phenylboronic acid, **5e** was obtained as a white solid (1.11 g, 84% yield). ^1H NMR (δ 23 °C, 400 MHz, $CDCl_3$): 8.58 (d, *J* = 8.7 Hz, 1H), 7.65 (dd, *J* = 8.7, 2.0 Hz, 1H), 7.57–7.48 (m, 4H), 7.35 (d, *J* = 8.0 Hz, 2H), 7.32 (br, 1H), 2.05 (s, 1H). The obtained spectral data are in agreement with those reported in the literature.[28]

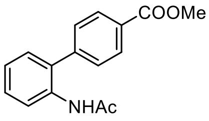

S5b

Methyl 2'-acetamido-[1,1'-biphenyl]-4-carboxylate (**S5b**). Prepared from 2-bromoaniline and 4-methoxycarbonylphenylboronic acid, **S5b** was obtained as a white solid (1.16 g, 77% yield). ^{1}H NMR (δ 23 °C, 400 MHz, $CDCl_3$): 8.21 (d, *J* = 8.3 Hz, 1H), 8.15 (d, *J* = 8.3 Hz, 2H), 7.47 (d, *J* = 8.3 Hz, 2H), 7.41 (t, *J* = 7.5 Hz, 1H), 7.26–7.19 (m, 2H), 6.98 (br, 1H), 3.96 (s, 3H), 2.03(s, 3H). The obtained spectral data are in agreement with those reported in the literature.[29]

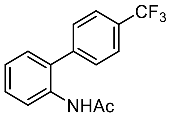

S5c

N-(4'-(trifluoromethyl)-[1,1'-biphenyl]-2-yl)acetamide (**S5c**). Prepared from 2-bromoaniline and 4-(trifluoromethyl)phenylboronic acid, **S5c** was obtained as a fluffy white solid (1.03 g, 66% yield). ^{1}H NMR (δ 23 °C, 400 MHz, $CDCl_3$): 8.19 (d, *J* = 8.1 Hz, 1H), 7.75 (d, *J* = 8.1 Hz, 2H), 7.51 (d, *J* = 7.9 Hz, 2H), 7.42 (ddd, *J* = 8.5, 6.0, 3.0, 1H), 7.24 (m, 2H), 6.92 (br, 1H), 2.05 (s, 3H). The obtained spectral data are in agreement with those reported in the literature.[30]

B.2 Intramolecular C–H Amination Electrocatalysis

General Procedure for Intramolecular Amination Reactions

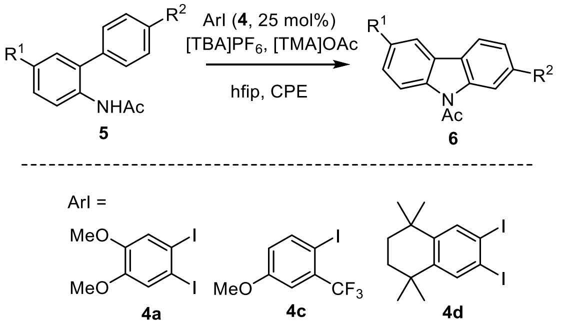

A 10-mL undivided electrochemical cell was charged with *N*-arylacetamide (**5**, 0.200 mmol, 1.00 equiv.), an aryl iodide catalyst (**4**, 50.0 μmol, 0.250 equiv.), [TMA]OAc (53.0 mg, 0.400 mmol, 2.00 equiv.), [TBA]PF_6 (390 mg, 1.01 mmol, 5.02 equiv.), and hfip (5.0 mL), and was fitted with a glassy carbon working electrode, a Pt counter electrode, and a Ag^+/Ag reference electrode. A constant potential of 1.40 V *vs.* Ag^+/Ag (1.22 V *vs.* Fc^+/Fc), 1.76 V *vs.* Ag^+/Ag (1.56 V *vs.* Fc^+/Fc), or 1.90 V *vs.* Ag^+/Ag (1.72 V *vs.* Fc^+/Fc) was applied to the reaction mixture when using **4a**, **4c**, or **4d**, respectively. The reaction was stirred at 23 °C until 50 C charge (~2.6 F/mol) was passed. The electrolysis was then stopped. 1,3,5-Trimethoxybenzene was added to the reaction mixture and an aliquot was taken for ^{1}H NMR analysis to determine NMR yield. The ^{1}H NMR spectra of carbazole products **6** were matched with those reported in the literature: **6a**,[31] **6b**,[31] **6c**,[32] **6d**,[33] and **6f**.[34] New compound, **6e** was isolated via preparative TLC (eluent 90:10 hexanes:ethyl acetate) and characterization data are detailed below.

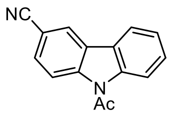

6e

9-acetyl-9H-carbazole-3-carbonitrile (**6e**). Prepared from **5e** with catalyst **4c** and obtained as a white solid (83% NMR yield). ^{1}H NMR (δ, 23 °C, 400 MHz, $CDCl_3$): 8.48 (d, *J* = 8.7 Hz, 1H), 8.30 (d, *J* = 1.5 Hz, 1H), 8.09 (d, *J* = 8.5 Hz, 1H), 8.05 (d, *J* = 7.6, 1H), 7.75 (dd, *J* = 8.7, 1.7 Hz, 1H), 7.59 (t, *J* = 7.8, 1H), 7.47 (t, *J* = 7.6 Hz, 1H), 2.93 (s, 3H). ^{13}C NMR (δ, 23 °C, 100 MHz, $CDCl_3$): 170.2, 139.0, 131.0, 128.8, 126.8, 125.1, 124.5, 124.2, 120.7, 119.3, 117.6, 116.0, 107.4, 77.4, 27.9. HRMS-ESI: calculated for $[C_{15}H_{10}N_2O+H]^+$ = 235.0866, observed $[C_{15}H_{10}N_2O+H]^+$ = 235.0863.

B.3 Intermolecular C–H Amination Electrocatalysis

General Procedure for Intermolecular Amination Reactions

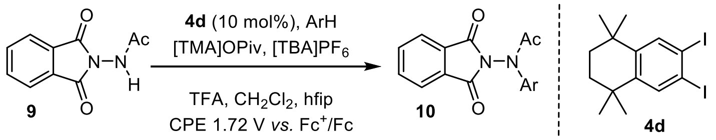

A 10-mL undivided electrochemical cell was charged with *N*-(1,3-dioxoisoindolin-2-yl)acetamide (**9**, 0.200 mmol, 1.00 equiv.), the appropriate arene (2.00 mmol, 10.0 equiv.), aryl iodide catalyst **4d** (8.8 mg, .020 mmol, 0.10 equiv.), [TMA]OPiv (70.1 mg, 0.400 mmol, 2.00 equiv.), [TBA]PF_6 (390 mg, 1.01 mmol, 5.02 equiv.), TFA (68.0 μL, 0.400 mmol, 2.00 equiv.), CH_2Cl_2 (0.8 mL), hfip (4.2 mL), and was fitted with a glassy carbon working electrode, a Pt counter electrode, and a Ag^+/Ag reference electrode. A constant potential of 1.90 V *vs.* Ag^+/Ag (1.72 V *vs.* Fc^+/Fc) was applied to the reaction mixture with stirring at 23 °C until 80 C charge (~4.2 F/mol) was passed. The electrolysis was then stopped. 1,3,5-Trimethoxybenzene was added to the reaction mixture and an aliquot was taken for ^{1}H NMR analysis to determine NMR yield. The ^{1}H NMR spectra of *N*-(1,3-dioxoisoindolin-2-yl)-*N*-arylacetamides **10a–10f** were well-matched to literature data.[35]

C. Supporting Data

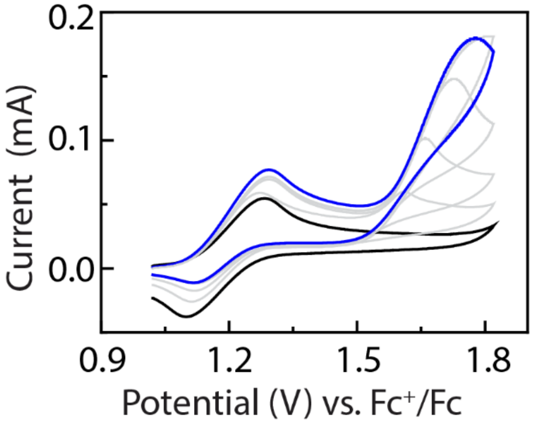

Figure S1. Cyclic voltammograms (CVs) of a 5.0 mM solution of **4a** in 0.20 M [TBA]PF_6/hfip. CVs were collected at varying concentrations of [TMA]OAc: 0.0 mM (—), 4.8, 8.9, 14.7, 18.7 (—), and 24.1 mM (—). CV conditions: Glassy carbon working electrode, Pt counter electrode, $AgNO_3$/Ag reference electrode, and scan rate of 100 mV/s. The CV was externally referenced to Fc^+/Fc.

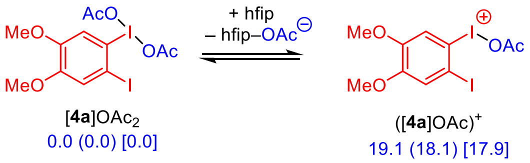

Figure S2. Computational evaluation of the speciation of I(III) derivative of [**4a**]OAc_2. Acetate dissociation is unfavorable. This calculation was carried out using UB3LYP-D3/ DGDZVP2- DGDZVP (I)-SMD (2-methyl-1-propanol) level of theory, ΔE (ΔH) [ΔG].

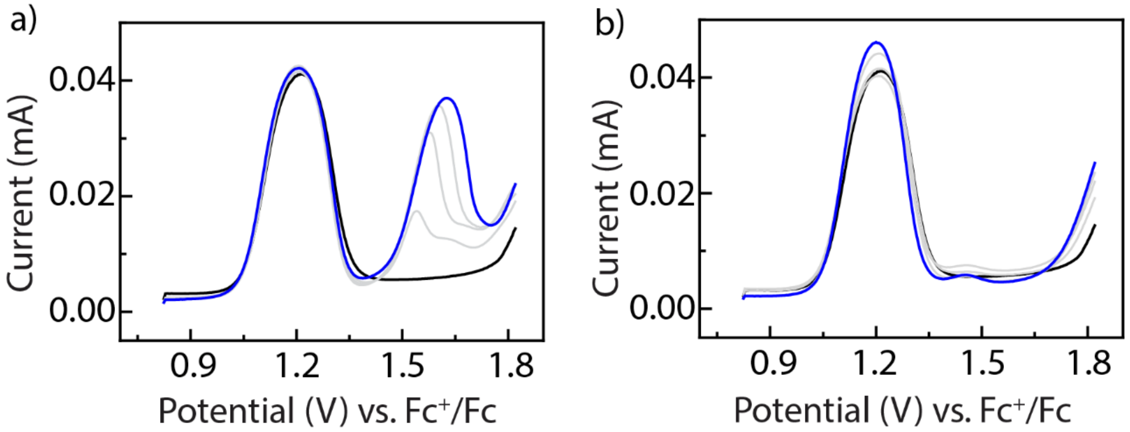

Figure S3. Square wave voltammograms (SWVs) of a 5.0 mM solution of **4a** in 0.20 M [TBA]PF_6/hfip varying a) [TMA]OPiv concentration: 0.0 mM (—), 4.7, 10.5, 18.3 (—), and 23.0 mM (—); and b) [TMA]TFA concentration: 0.0 mM (—), 5.2, 16.2, 21.4 (—), and 26.2 mM (—). SWV conditions: Glassy carbon working electrode, Pt counter electrode, and Ag^+/Ag reference electrode, and pulsed at 15 Hz with 25 mV amplitude and 4 mV increments. The SWV was externally referenced to Fc^+/Fc.

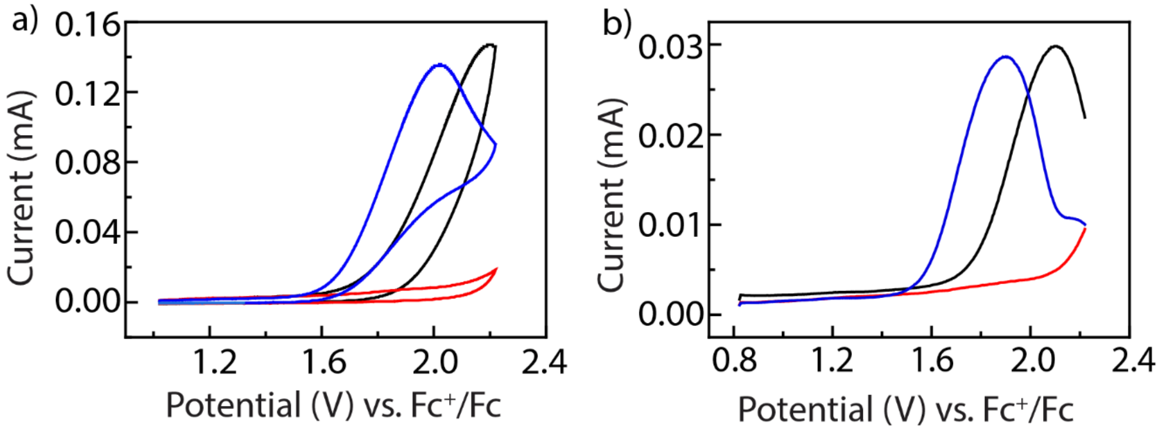

Figure S4. A) CVs and b) SWVs of 35 mM solutions of [TMA]OAc (—), [TMA]OPiv (—), and [TMA]TFA (—) in 0.20 M [TBA]PF_6/hfip. CV conditions: Glassy carbon working electrode, Pt counter electrode, Ag^+/Ag reference electrode, and scan rate of 100 mV/s. SWV conditions: Glassy carbon working electrode, Pt counter electrode, Ag^+/Ag reference electrode, and pulsed at 15 Hz with 25 mV amplitude and 4 mV increments. The CV and SWV were externally referenced to Fc^+/Fc.

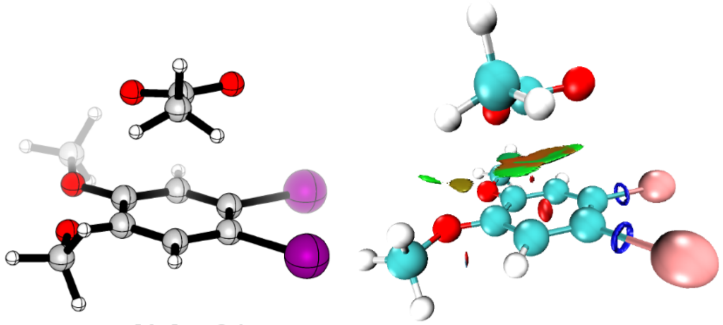

$[\mathbf{4a}]^{+}\cdots OAc^{-}$

Figure S5. A noncovalent interactions plot (NCIPLOT) showing weak Van-Der Waals interaction between acetate and the π face of [**4a**]+. The NCIPLOT was calculated using UB3LYP-D3/ DGDZVP2- DGDZVP (I)-SMD (2-methyl-1-propanol) level of theory.

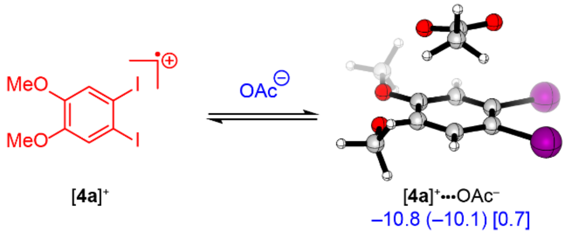

Figure S6. Computational evaluation of binding of [**4a**]$^+$ with unsolvated acetate. Computations were performed using UB3LYP-D3/ DGDZVP2- DGDZVP (I)-SMD (2-methyl-1-propanol) level of theory, ΔE (ΔH) [ΔG].

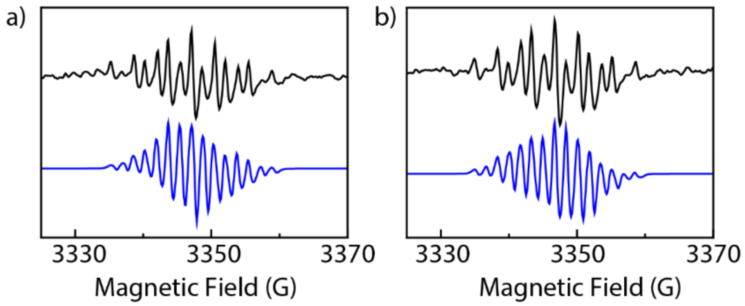

Figure S7. a) *In situ* EPR collected during the electrolysis of 1.0 mM **4a** in 0.20 M [TBA]PF_6/hfip at 1.40 V *vs.* Ag^+/Ag (1.22 V *vs.* Fc^+/Fc) (—) and simulated spectrum (—). b) *In situ* EPR collected during the electrolysis of 1.0 mM **4a** with 4.0 mM [TMA]OAc in 0.20 M [TBA]PF_6/hfip at 1.40 V *vs.* Ag^+/Ag (1.22 V *vs.* Fc^+/Fc) (—) and simulated spectrum (—). Electrolysis condition: Undivided electrochemical cell, glassy carbon working electrode, Pt counter electrode, and Ag^+/Ag reference electrode.

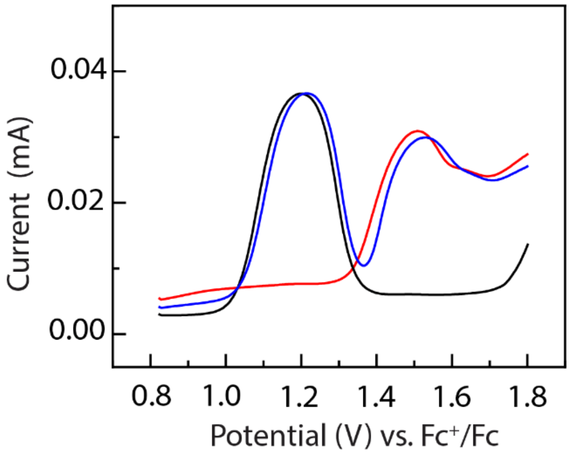

Figure S8. Square wave voltammograms (SWVs) of 0.20 M [TBA]PF_6/hfip solutions containing 5.0 mM **4a** (—), 10.0 mM **5a** (—), and mixture of 5.0 mM **4a** and 10.0 mM **5a** (—). SWV conditions: Glassy carbon working electrode, Pt counter electrode, and Ag^+/Ag reference electrode, and pulsed at 15 Hz with 25 mV amplitude and 4 mV increments. The SWV was externally referenced to Fc^+/Fc.

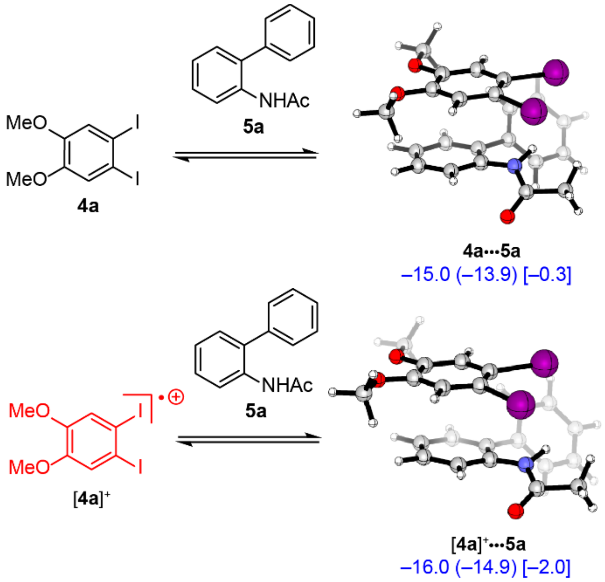

Figure S9. Computational evaluation of potential speciation equilibria of **4a** and [**4a**]$^+$ with **5**. Calculations were performed using UB3LYP-D3/ DGDZVP2- DGDZVP (I)-SMD (2-methyl-1-propanol) level of theory, ΔE (ΔH) [ΔG].

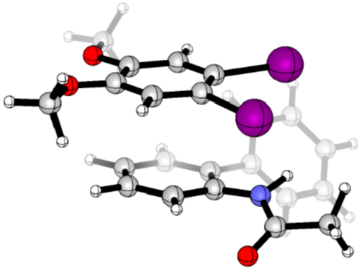

[4a]$^{+}$•••5a

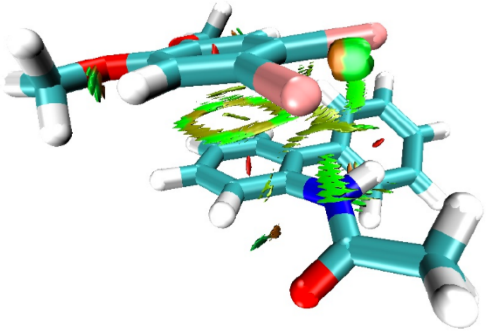

Figure S10. A noncovalent interactions plot (NCIPLOT) showing weak Van-Der Waals interaction via π-π stacking between **5a** and [**4a**]$^{+}$. The NCIPLOT was calculated using UB3LYP-D3/ DGDZVP2- DGDZVP (I)-SMD (2-methyl-1-propanol) level of theory.

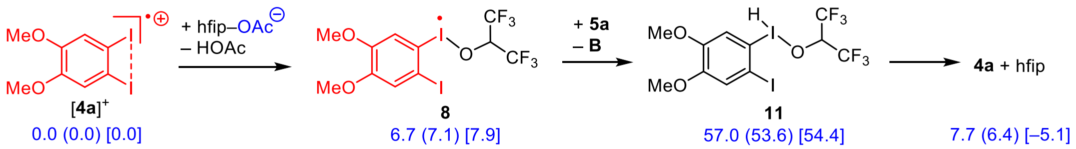

Figure S11. Computational evaluation of HAT from **5a** intermediate to **8** and subsequent reductive elimination from **11** to generate **4a** and hfip. The large energy penalty of the HAT step suggests this mechanism is unlikely to take place. This calculation was carried out using UB3LYP-D3/ DGDZVP2- DGDZVP (I)-SMD (2-methyl-1-propanol) level of theory, ΔE (ΔH) [ΔG].

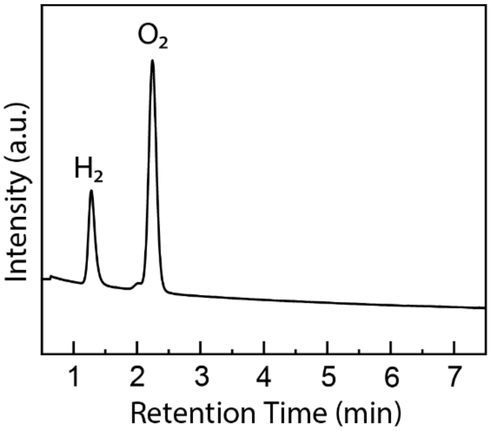

Figure S12. Gas chromatograph of headspace following electrolysis of **4a** in the presence of [TMA]OAc at 1.22 V *vs.* Fc^+/Fc. Procedure: An electrochemical cell was charged with 1,2-diiodo-4,5-dimethoxybenzene (**4a**, 78.0 mg, 0.200 mmol, 1.00 equiv.), [TMA]OAc (106 mg, 0.800 mmol, 4.00 equiv.), [TBA]PF_6 (390 mg, 1.01 mmol, 5.02 equiv.), and hfip (5.0 mL), and was fitted with a glassy carbon working electrode, a Pt counter electrode, and a Ag^+/Ag reference electrode. A constant potential of 1.40 V *vs.* Ag^+/Ag (1.22 V *vs.* Fc^+/Fc) was applied to the reaction mixture with stirring at 23 °C until 50 C charge (2.6 F/mol) was passed. The electrolysis was then stopped, and the headspace of the reaction mixture was analyzed by gas chromatography. None of the decarboxylation products expected from acetate oxidation (*i.e.*, methane, ethane, CO_2) were observed. The oxygen (O_2) peak (retention time (t) = 2.3 min) was due to the ambient conditions used for reaction setup. The hydrogen (H_2) peak (t = 1.3 min) was due to the reduction of solvent. The headspace volume was 9.5 cm^3. The volume of the empty reaction chamber (14.5 cm^3) was determined via measuring the volume of acetone needed to fill the chamber.

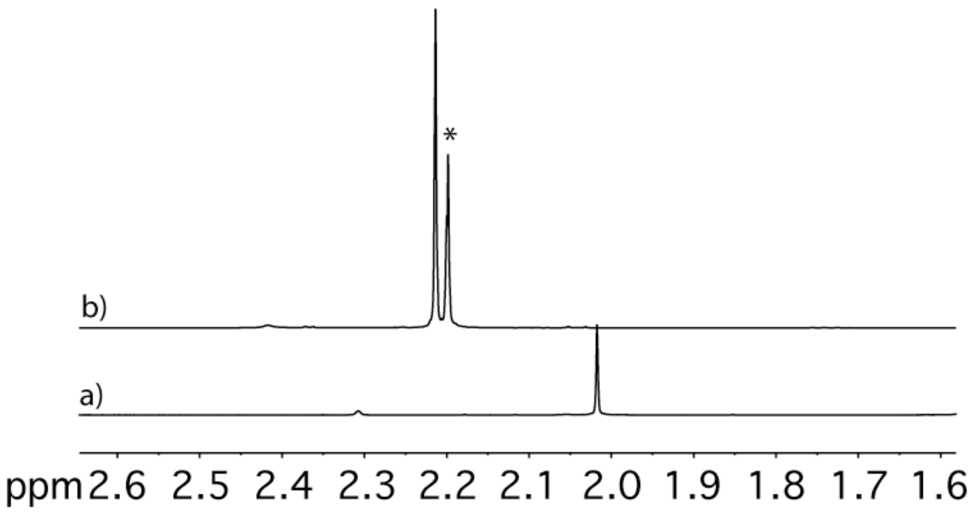

Figure S13. ^{1}H NMR spectra of a) an aliquot of an electrolyzed solution of **4a** (0.1 mmol, 1.0 equiv.) and [TMA]OAc (0.20 mmol, 2.0 equiv.) at 1.40 V *vs.* Ag^+/Ag (1.22 V *vs.* Fc^+/Fc) after 1.0 F/mol of charge was passed (electrolysis conditions: Undivided cell, glassy carbon working electrode, Pt counter electrode, and Ag^+/Ag reference electrode) and b) independently synthesized diacetyl peroxide (2.19 ppm, *) with acetic anhydride (2.21 ppm). Spectra were measured at 400 MHz in $CDCl_3$ at 23 °C.

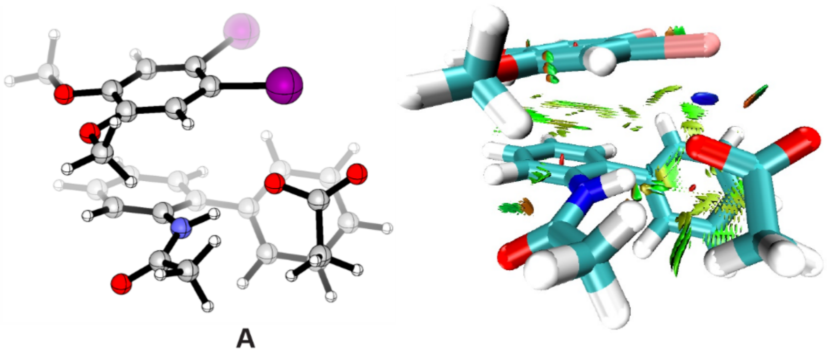

A

Figure S14. A noncovalent interactions plot (NCIPLOT) showing weak Van-Der Waals interactions between **5a,** acetate, and [**4a**]$^+$. The NCIPLOT was calculated using UB3LYP-D3/ DGDZVP2- DGDZVP (I)-SMD (2-methyl-1-propanol) level of theory.

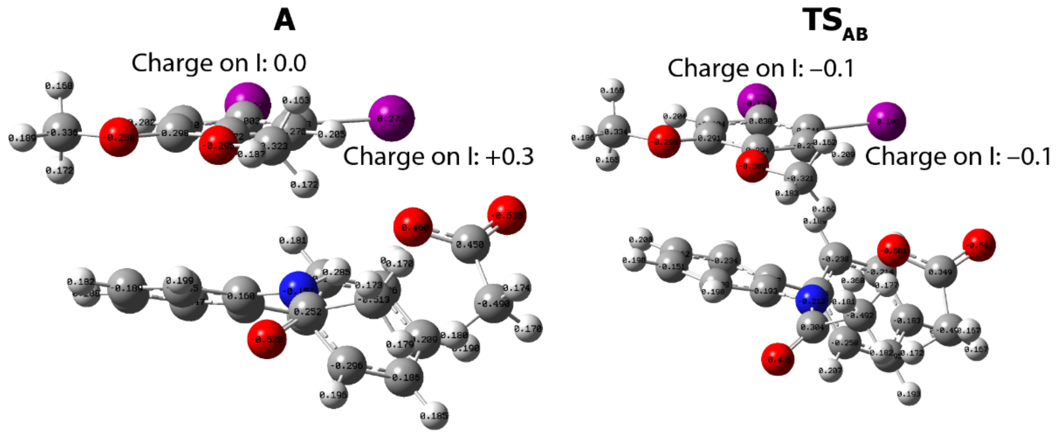

Figure S15. Mulliken charge analysis of ternary complex **A** versus MS-PCET transition state $\mathbf{TS_{AB}}$, which shows that iodine is more oxidized in **A** than in $\mathbf{TS_{AB}}$. Computations were performed using UB3LYP-D3/ DGDZVP2- DGDZVP (I)-SMD (2-methyl-1-propanol) level of theory.

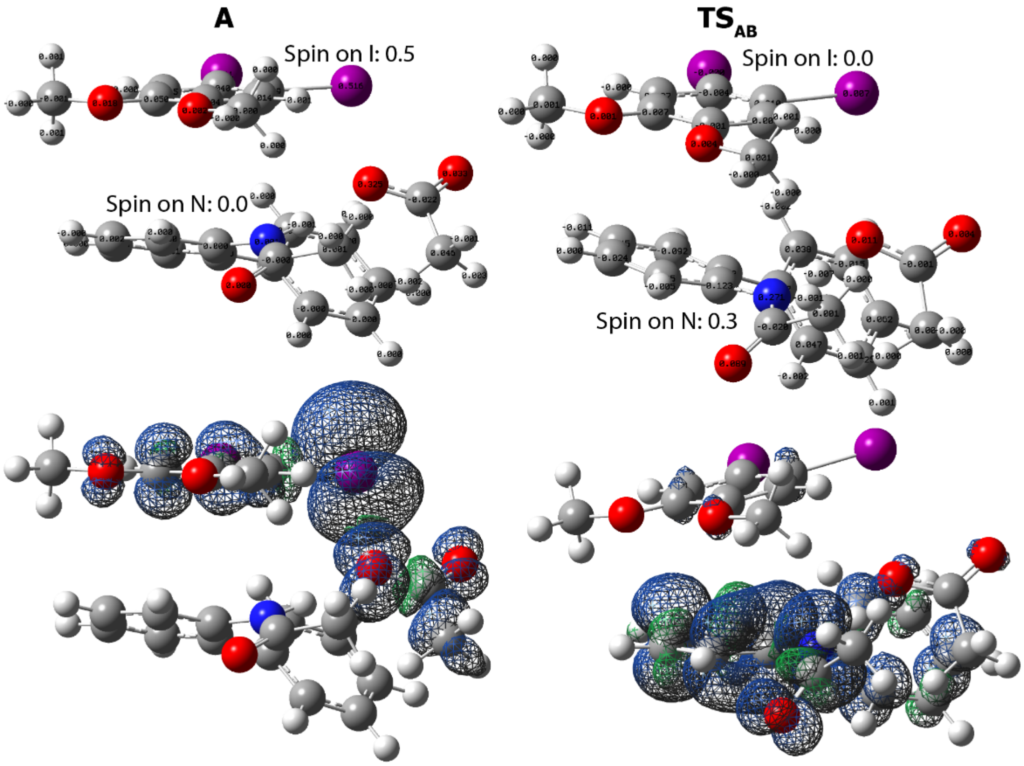

Figure S16. Mulliken spin density analysis and spin-isosurface plots of ternary complex **A** versus MS-PCET transition state **TS$_{AB}$**, which shows that the spin density shifts from iodine (0.5→0.0) to nitrogen (0.0→0.2) during the MS-PCET process. Computations were performed using UB3LYP-D3/ DGDZVP2- DGDZVP (I)-SMD (2-methyl-1-propanol) level of theory.

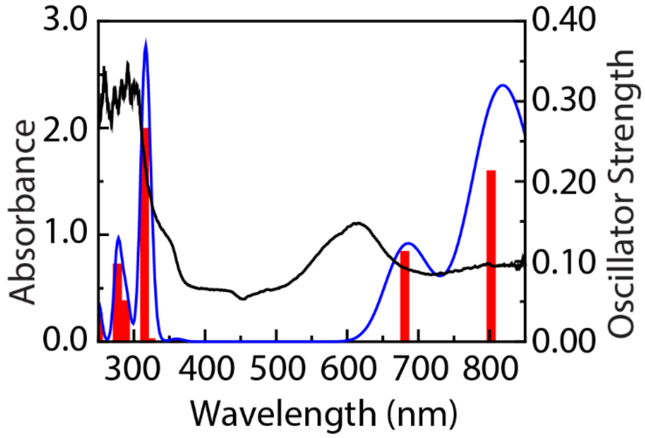

Figure S17. UV-vis spectrum (—), calculated TD–DFT absorption spectrum (—), and electronic configurations of excited states of [**4c**]$^{+}$ (—). Experimental setup: A 1.0 mm quart cell was charged with a hfip solution 5.0 mM **4c** and 0.20 M $TBAPF_6$, a Pt honeycomb dual working/counter electrode, and a Ag^{+}/Ag reference electrode. UV-vis data was collected in conjunction with electrolysis at 1.76 V *vs.* Ag^{+}/Ag (1.56 V *vs.* Fc^{+}/Fc).

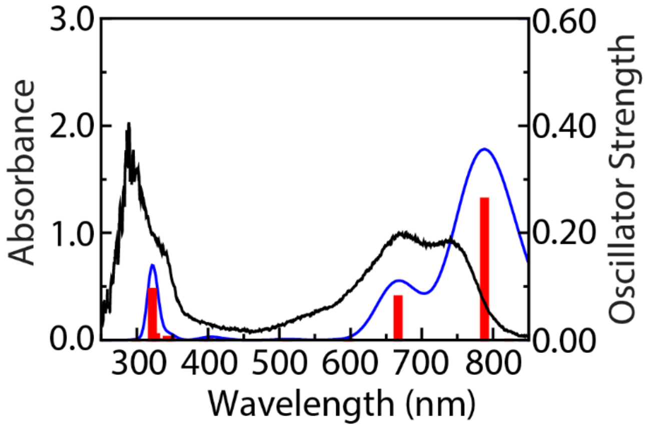

Figure S18. UV-vis spectrum (—), calculated TD−DFT absorption spectrum (—), and electronic configurations of excited states of [**4d**]$^+$ (—). Experimental setup: A 1.0 mm quart cell was charged with a tfe solution 5.0 mM **4d** and 0.20 M $TBAPF_6$, a Pt honeycomb dual working/counter electrode, and a Ag^+/Ag reference electrode. UV-vis data was collected in conjunction with electrolysis at 1.90 V *vs.* Ag^+/Ag (1.72 V *vs.* Fc^+/Fc) at –30°C.

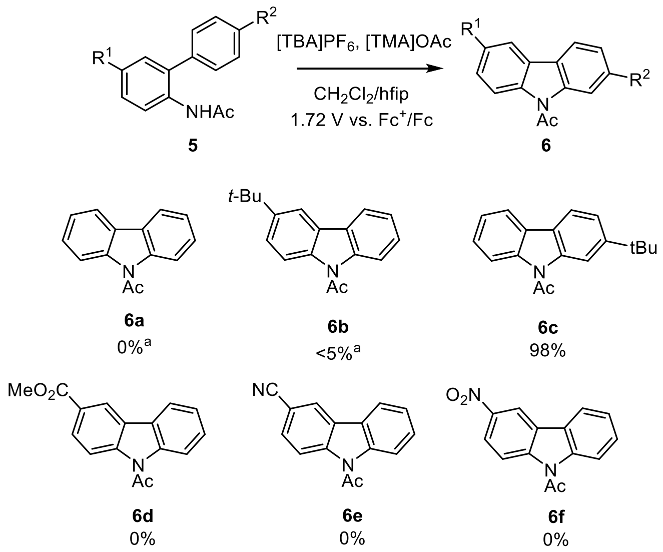

Figure S19. Evaluation of uncatalyzed background reactions of *N*-arylacetamides **5**. Yields are NMR yields. Standard conditions: **5** (0.20 mmol, 40 mM in 5.0 mL hfip), [TMA]OAc (2.0 equiv), $[TBA]PF_6$ (0.20 M), CPE at 1.72 V *vs.* Fc^+/Fc. CPE for ~50 C (2.6 F/mol), undivided cell, glassy anode, platinum cathode, and Ag^+/Ag reference. [a]Plating was observed on the working electrode after electrolysis.

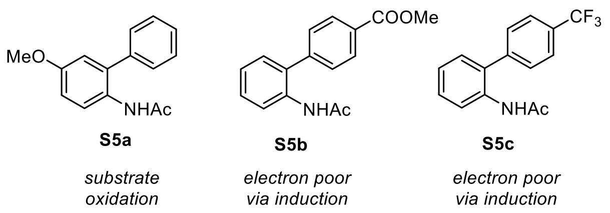

Figure S20. Summary of challenging substrates for the intramolecular C–H/N–H coupling electrocatalysis mediated by **4a**, **4c**, or **4d**.

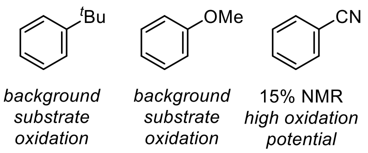

Figure S21. Challenging substrates for intermolecular C–H/N–H coupling electrocatalysis mediated by **4d**.

D. Mechanistic Data for 4-Iodoanisole Catalysis

We have carried out experiments with 4-iodoanisole (**4b**) that are analogous to those discussed for 1,2-diiodoveratrole (**4a**) in the main text. The conclusions of these experiments are consistent with that of **4a**, suggesting that N–H bond activation by **4b** and base proceeds via a PCET mechanism. SWV analysis indicates that acetate does not significantly affect the first oxidation wave of **4b** at 1.38 V *vs.* Fc+/Fc (Figure S22a), and that the iodanyl radical [**4b**]$^+$ together with acetate forms an adduct [**4b**]OAc, which displays an oxidative feature at 1.62 V *vs.* Fc^+/Fc. Use of the stronger base pivalate results in observation of the second oxidation feature at 1.55 V *vs.* Fc^+/Fc (Figure S22b), while use of the weaker base trifluoroacetate forms results in observation of the second oxidation feature at 1.85 V *vs.* Fc^+/Fc (Figure S22c). These features are obscured when CV experiments out carried out as the oxidative features of **4b** and [**4b**]OAc overlap (Figure S23).

Computational studies illustrated in Figure S24 show that acetate prefers to bind to iodine in [**4b**]$^+$ resulting in a acetate-stabilized I(II) compound with an I–O bond (**4b**–OAc, 1.4 kcal/mol), instead of binding to the π face of [**4b**]$^+$ to form a contact ion pair ([**4b**]$^+\cdots$OAc$^-$, 3.9 kcal/mol). The formation of [**4b**]–OAc by [**4b**]$^+$ and unsolvated acetate to make **4b**–OAc is favored by 1.1 kcal/mol (Figure S25). Compared to the *in situ* EPR spectrum of [**4b**]$^+$, the *in situ* EPR spectrum of [**4b**]$^+$ in the presence of acetate shows some perturbation by acetate without change in the hyperfine coupling (Figure S26).

The binding of substrate **5a** to **4b** or [**4b**]$^+$ was both thermoneutral (–1.3 and –0.9 kcal/mol, respectively) due to favorable π-π stacking (Figure S27, NCIPLOT between **5a** and [**4b**]$^+$ is provided in Figure S28). Previous electroanalytical experiment showed that substrate **5a** does not turn over catalyst **4b** on CV time scale.[8]

We carried out computational and experiment studies to analyze the various mechanisms by which [**4b**]$^+$ activates N–H bond (Figure S29). Disproportionation of iodanyl radical to generate [**4b**]OAc_2 is unlikely because the coresponding I(III) product was unobserved (Figure S29, path a). HAT at iodine to generate $\mathbf{7_{4b}}$ is excluded by the high energy barrier of 41.9 kcal/mol (Figure S29, path b). Reaction between [**4b**]$^+$ and hfip to generate iodanyl radical $\mathbf{8_{4b}}$ (ΔG = 2.2 kcal/mol) followed by I–O bond homolysis (ΔG = –10.4 kcal/mol) suggests that this pathway could be operational (Figure S29, path c). Meanwhile, HAT from **5a** to intermediate $\mathbf{8_{4b}}$ (ΔG = 50.1 kcal/mol) to generate $\mathbf{11_{4b}}$ was excluded due to high energetics. Acetoxy radical pathway (Figure S29, path d) was excluded since GC-headspace analysis shows no evidence of CO_2 evolution (Figure S30). The PCET mechanism has a favorable energetics (–10.4 kcal/mol, Figure S29, path e) and proceeds via a complex $\mathbf{A_{4b}}$ form by between **5a**, [**4b**]$^+$, and acetate (Figure S31a; NCIPLOT is provided in Figure S32). The coresponding PT-ET pathway is disfavored by 21.0 kcal/mol, while the ET-PT pathway is thermoneutral at 1.5 kcal/mol (Figure S31b). Due to similar engergetics of the PCET and ET-PT pathways, both mechanisms could be operational. The full mechanism involving [**4b**]$^+$ is detailed in Figure S33, in which substrate **5a** activation via PCET and product **6a** formation steps are similar to those of [**4a**]$^+$.

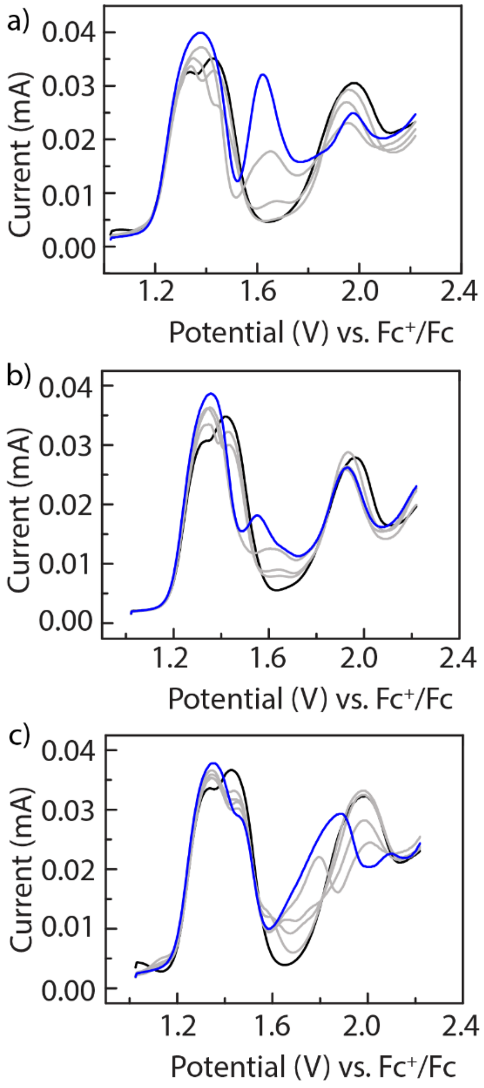

Figure S22. SWVs of 5.0 mM solutions of 4-iodoanisole (**4b**) in 0.20 M [TBA]PF_6/hfip. SWVs were collected at varying a) [TMA]OAc concentration: 0.0 mM (—), 2.8, 7.4, 16.0 (—), and 33.0 mM (—); b) [TMA]OPiv concentration: 0.0 mM (—), 5.82, 7.88, 11.9 (—), and 18.0 mM (—); and c) [TMA]TFA concentration: 0.0 mM (—), 4.60, 7.60, 11.6, 16.3 (—), and 20.7 mM (—). SWV conditions: Glassy carbon working electrode, Pt counter electrode, and Ag^+/Ag reference electrode, and pulsed at 15 Hz with 25 mV amplitude and 4 mV increments. SWVs were externally referenced to Fc^+/Fc.

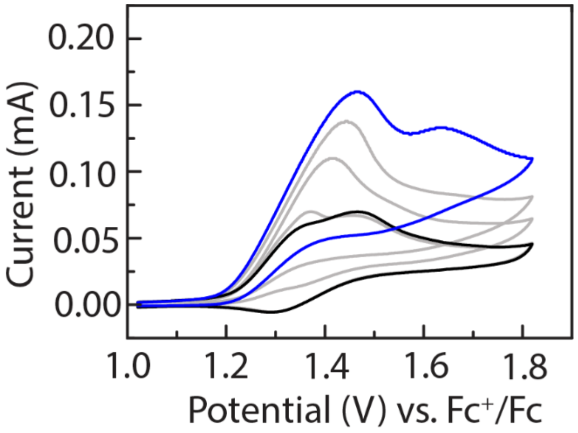

Figure S23. CVs of a 5.0 mM solution of **4b** in 0.20 M [TBA]PF_6/hfip. CVs were collected with varying [TMA]OAc concentration: 0.0 mM (—), 5.0, 12.5, 20.9 (—), and 36.9 mM (—). CV conditions: Glassy carbon working electrode, Pt counter electrode, $AgNO_3$/Ag reference electrode, and scan rate of 100 mV/s. The CV was externally referenced to Fc^+/Fc.

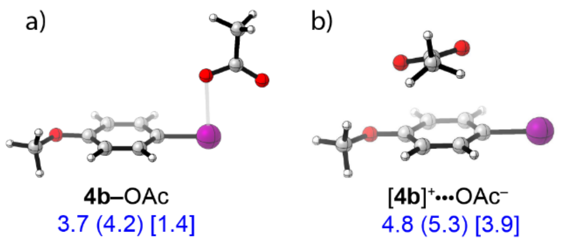

Figure S24. Computational evaluation of various acetate coordination modes with [**4b**]$^+$: (a) A neutral acetate-stabilized I(II) compound (**4b**–OAc) or b) a contact ion pair in which the acetate binds to the π face of [**4b**]$^+$ ([**4b**]$^+\cdots$OAc$^-$). The energies are referenced versus a solvent-separated ion pair of [**4b**]OAc in which the acetate ion is engaged in H-bonding with hfip (*i.e.*, [**4b**]$^+$ and hfip–OAc$^-$). Computations were performed using UB3LYP-D3/ DGDZVP2-DGDZVP (I)-SMD (2-methyl-1-propanol) level of theory, ΔE (ΔH) [ΔG].

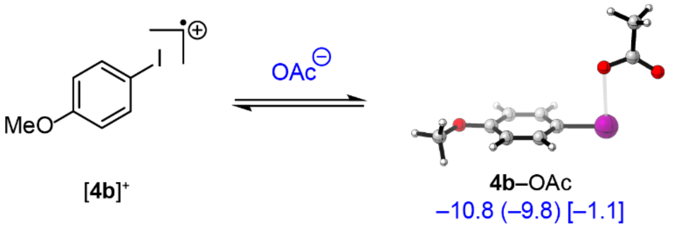

Figure S25. Computational evaluation of the binding of unsolvated acetate to [**4b**]$^{+}$. Computations were performed using UB3LYP-D3/ DGDZVP2- DGDZVP (I)-SMD (2-methyl-1-propanol) level of theory, ΔE (ΔH) [ΔG].

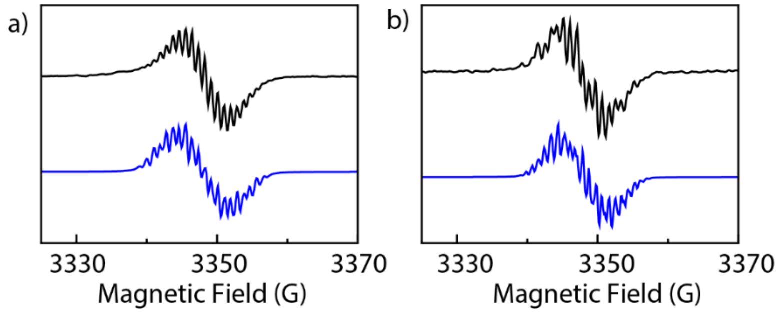

Figure S26. a) *In situ* EPR collected during the electrolysis of 1.0 mM **4b** in 0.20 M [TBA]PF_6/hfip at 1.60 V *vs.* Ag^+/Ag (1.42 V *vs.* Fc^+/Fc) (—) and simulated spectrum (—). b) *In situ* EPR collected during the electrolysis of 1.0 mM **4b** with 4.0 mM [TMA]OAc in 0.20 M [TBA]PF_6/hfip at 1.60 V *vs.* Ag^+/Ag (1.42 V *vs.* Fc^+/Fc) (—) and simulated spectrum (—). Electrolysis condition: Undivided electrochemical cell, glassy carbon working electrode, Pt counter electrode, and Ag^+/Ag reference electrode.

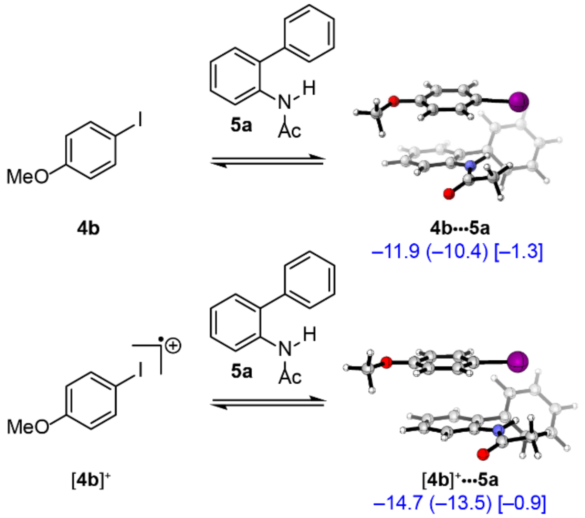

Figure S27. Computational evaluation of potential speciation equilibria of **4b** and [**4b**]+ with **5a**. Computations were performed using UB3LYP-D3/ DGDZVP2- DGDZVP (I)-SMD (2-methyl-1-propanol) level of theory, ΔE (ΔH) [ΔG].

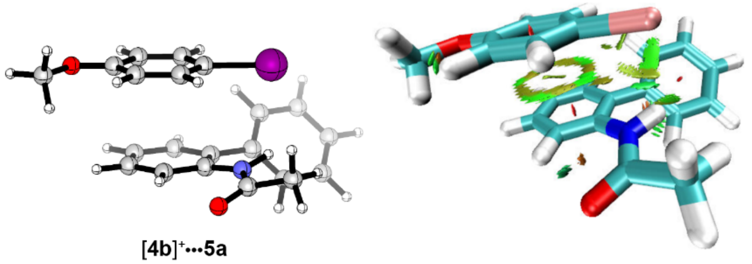
[4b]+•••5a

Figure S28. A noncovalent interactions plot (NCIPLOT) showing weak Van-Der Waals interaction via π-π stacking between **5a** and [**4b**]+. The NCIPLOT was calculated using UB3LYP-D3/ DGDZVP2- DGDZVP (I)-SMD (2-methyl-1-propanol) level of theory.

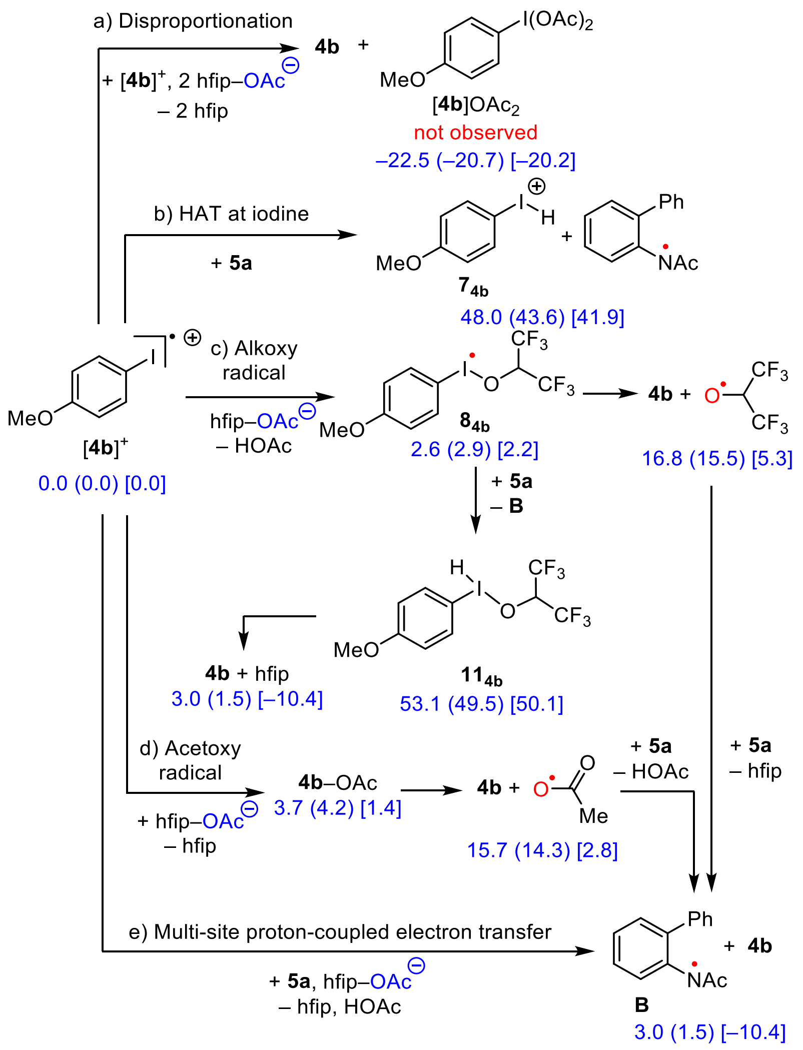

Figure S29. Potential reaction pathways available for [**4b**]+. Computations were performed using UB3LYP-D3/ DGDZVP2- DGDZVP (I)-SMD (2-methyl-1-propanol) level of theory , ΔE (ΔH) [ΔG].

NMR and headspace analysis of the electrolysis of 4-MeO-ArI with [TMA]OAc in hfip

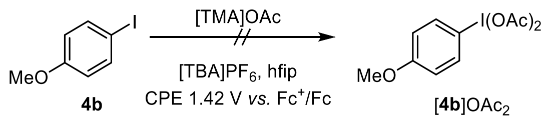

A 10-mL glass vial was charged with 4-iodoanisole (**4b**, 45.6 mg, 0.200 mmol, 1.00 equiv.), [TMA]OAc (55.9 mg, 0.420 mmol, 2.10 equiv.), [TBA]PF_6 (390 mg, 1.01 mmol, 5.02 equiv.), and hfip (5.0 mL) and was fitted with a glassy carbon working electrode, a Pt counter electrode, and a Ag^+/Ag reference electrode. A constant potential of 1.60V *vs.* Ag^+/Ag (1.42 V *vs.* Fc^+/Fc) was applied to the reaction mixture with stirring at 23 °C until ~80 C charge (~4.2 F/mol) was passed. The headspace of the reaction mixture was analyzed by gas chromatography to determine if any gaseous products were produced during electrolysis. Data is collected in Figure S30. Following electrolysis, 1,3,5-trimethoxybenzene was added to the reaction mixture and an aliquot was taken for ^{1}H NMR analysis, which indicated that 13% **4b** remained and no I(III) species were observed.

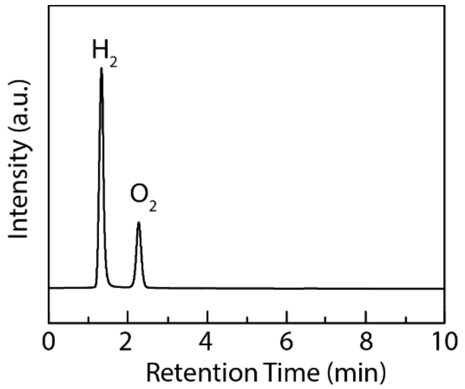

Figure S30. Gas chromatograph of headspace following the electrolysis of **4b** in the presence of [TMA]OAc at 1.42 V *vs.* Fc^+/Fc. The oxygen (O_2) peak was due to the ambient conditions used for reaction setup. The hydrogen (H_2) peak was due to the reduction of solvent. No decarboxylated products from acetate oxidation were observed. The headspace volume was 9.5 cm^3. The volume of the empty reaction chamber (14.5 cm^3) was determined via measuring the volume of acetone needed to fill the chamber.

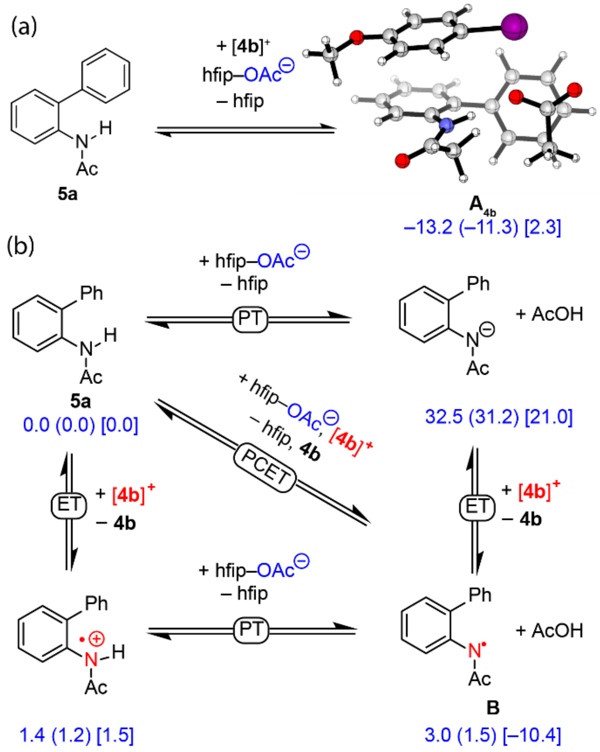

Figure S31. a) Computations of binding between [**4b**]+, **5a**, and acetate b) PCET square diagram for N–H bond activation using at iodanyl radical [**4b**]+ as oxidant and acetate as base. Computations were performed using UB3LYP-D3/ DGDZVP2- DGDZVP (I)-SMD (2-methyl-1-propanol) level of theory, ΔE (ΔH) [ΔG].

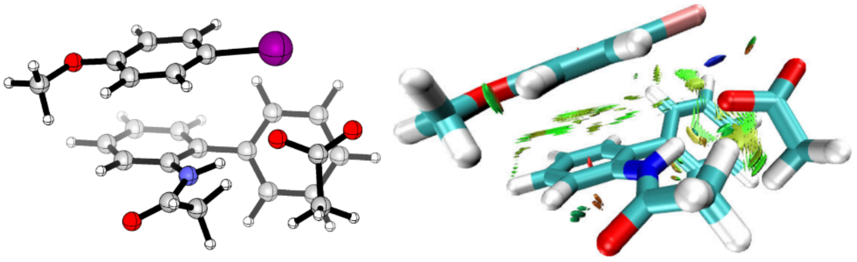

A_{4b}

Figure S32. A noncovalent interactions plot (NCIPLOT) showing weak Van-Der Waals interactions between **5a,** acetate, and [**4b**]$^{+}$. The NCIPLOT was calculated using UB3LYP-D3/ DGDZVP2- DGDZVP (I)-SMD (2-methyl-1-propanol) level of theory.

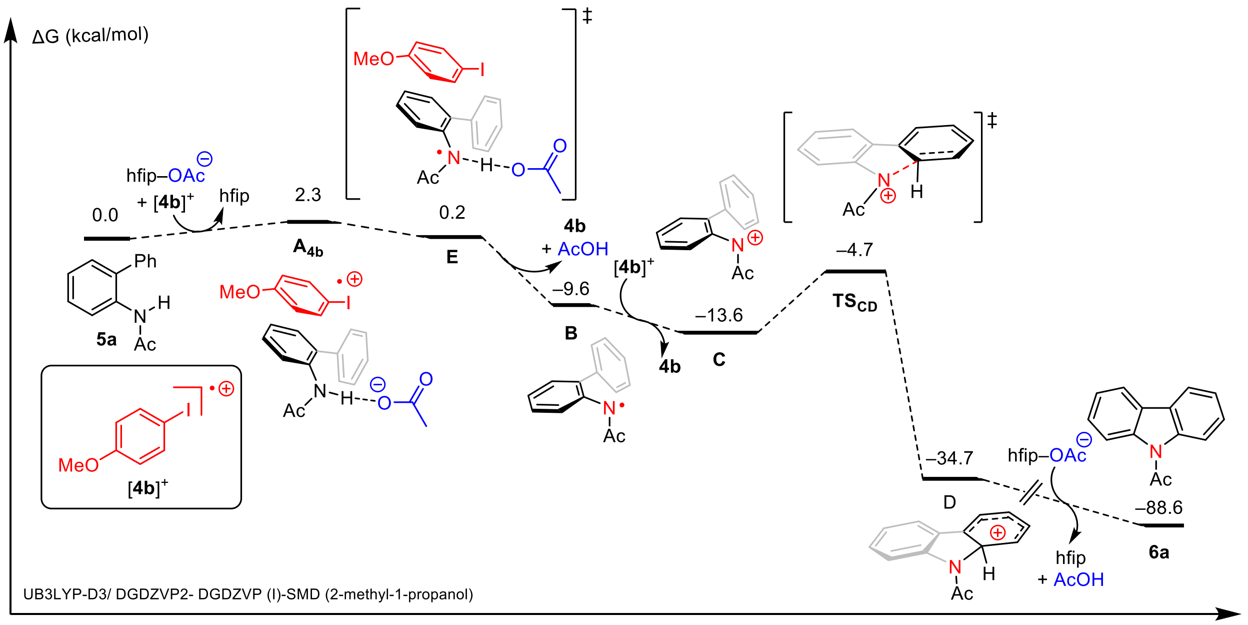

Figure S33. Reaction coordinate diagram of electrochemical catalyzed C–H/N–H coupling of **5a** by iodanyl radical [**4b**]+ and acetate. Computations were performed using UB3LYP-D3/ DGDZVP2- DGDZVP (I)-SMD (2-methyl-1-propanol) level of theory, ΔG; ΔE and ΔH were omitted for clarity.

E. Computational Data

E.1 Optimized Coordinates Associated with 1,2-Diiodoveratrole

Table S1. Optimized coordinates of **4a**.

C	−2.52239	−0.71034	0.00029
C	−1.30904	−1.39491	0.00060
C	−0.08258	−0.69900	0.00033
C	−0.08258	0.69900	−0.00033
C	−1.30904	1.39491	−0.00060
C	−2.52239	0.71034	−0.00029
I	1.68190	−1.90544	0.00103
I	1.68190	1.90544	−0.00103
H	−1.30277	−2.47697	0.00108
H	−1.30277	2.47697	−0.00108
O	−3.75279	1.30466	−0.00052
O	−3.75279	−1.30466	0.00052
C	−3.80472	2.74273	−0.00093
C	−3.80472	−2.74273	0.00093
H	−4.86622	−2.99603	0.00097
H	−3.32966	−3.15396	0.89897
H	−3.32962	−3.15446	−0.89687
H	−4.86622	2.99603	−0.00097
H	−3.32966	3.15396	−0.89897
H	−3.32962	3.15446	0.89687

E (UB3LYP) = −14299.9396734
H (Sum of electronic and thermal enthalpies) = −14299.781692
G (Sum of electronic and thermal free energies) = −14299.837760

Table S2. Optimized coordinates of [**4a**]$^{+}$.

C	−2.50258	−0.72750	0.00016
C	−1.28304	−1.41871	−0.00423
C	−0.07610	−0.72437	−0.00538
C	−0.07610	0.72437	0.00538
C	−1.28304	1.41871	0.00423
C	−2.50258	0.72750	−0.00016
I	1.67965	−1.88919	−0.02777
I	1.67965	1.88919	0.02777
H	−1.28213	−2.50072	−0.00899
H	−1.28213	2.50072	0.00899
O	−3.70357	1.28736	−0.00287
O	−3.70357	−1.28736	0.00287
C	−3.82526	2.73651	−0.00708
C	−3.82526	−2.73651	0.00708
H	−4.89776	−2.92678	0.01176
H	−3.36005	−3.14652	0.90769
H	−3.36705	−3.15135	−0.89487
H	−4.89776	2.92678	−0.01176
H	−3.36005	3.14652	−0.90769
H	−3.36705	3.15135	0.89487

E (UB3LYP) = −14299.7346633
H (Sum of electronic and thermal enthalpies) = −14299.576470
G (Sum of electronic and thermal free energies) = −14299.632413

Table S3. Optimized coordinates of **4a**–OAc.

C	–1.32273	–0.92539	–0.17355
C	–0.11242	–1.58906	–0.00987
C	0.85343	–1.11378	0.90403
C	0.60065	0.04287	1.66000
C	–0.62204	0.71357	1.49315
C	–1.58309	0.24634	0.59341
I	2.63348	–2.23060	1.03632
H	0.10082	–2.47820	–0.58840
H	–0.82018	1.60723	2.06973
O	–2.78629	0.83665	0.37711
C	–3.10308	2.03030	1.12146
H	–4.10242	2.31911	0.79282
H	–2.39314	2.83260	0.89305
H	–3.11242	1.82800	2.19798
O	3.66095	–0.32014	–0.50577
C	4.92834	–0.53093	–0.70051
O	5.58181	–1.51227	–0.28249
C	5.62043	0.57609	–1.49782
H	4.90826	1.17092	–2.07185
H	6.13327	1.22741	–0.78191
H	6.36649	0.12688	–2.15772
I	1.97173	0.89539	3.05182
O	–2.31303	–1.30547	–1.02942
C	–2.08633	–2.47426	–1.83986
H	–1.21875	–2.33475	–2.49458
H	–1.94745	–3.36385	–1.21497
H	–2.98744	–2.58848	–2.44433

E (UB3LYP) = –14528.444260
H (Sum of electronic and thermal enthalpies) = –14528.230514
G (Sum of electronic and thermal free energies) = –14528.305515

Table S4. Optimized coordinates of [**4a**]$^{+}$···OAc^{-}.

C	−1.85438	−1.27577	−0.56886
C	−1.00228	−2.16212	0.07615
C	0.03326	−1.65889	0.94807
C	0.15839	−0.28891	1.16125
C	−0.69207	0.61362	0.50975
C	−1.71597	0.10904	−0.38833
I	1.43201	−2.89359	1.93305
O	−2.46810	1.03425	−0.96387
C	−3.45258	0.63540	−1.95405
H	−3.89335	1.56834	−2.30363
H	−4.21658	0.00400	−1.49173
H	−2.95697	0.11432	−2.77741
H	0.93681	0.08501	1.81209
H	−2.61742	−1.65098	−1.23704
O	0.32321	0.34975	−2.31770
C	1.25862	−0.45145	−1.97629
O	1.24756	−1.70801	−2.18440
C	2.49019	0.14070	−1.28569
H	2.26833	1.11305	−0.83998
H	2.87366	−0.54431	−0.52488
H	3.27507	0.27739	−2.03934
I	−1.31920	−4.21574	−0.29585
O	−0.64634	1.93410	0.63040
C	0.36285	2.53658	1.48239
H	0.22456	2.20917	2.51676
H	1.36143	2.27722	1.12043
H	0.19657	3.61001	1.39901

E (UB3LYP) = −14528.4521005
H (Sum of electronic and thermal enthalpies) = −14528.238026
G (Sum of electronic and thermal free energies) = −14528.311065

Table S5. Optimized coordinates of **4a**···**5a**.

C	−1.38135	−1.11762	0.33687
C	−0.48484	−0.04277	0.24816
C	−0.94846	1.28832	0.38509
C	−2.31786	1.49248	0.62773
C	−3.21387	0.42303	0.70889
C	−2.74151	−0.88611	0.55703
N	0.90820	−0.27292	0.10840
C	1.52590	−1.22684	−0.64225
C	3.03460	−1.12048	−0.69700
O	0.91299	−2.10653	−1.27951
C	−0.03716	2.46405	0.30509
C	0.89815	2.59993	−0.73998
C	1.74196	3.71433	−0.80511
C	1.66490	4.71808	0.17097
C	0.72925	4.59947	1.20730
C	−0.11385	3.48513	1.27111
H	−1.00441	−2.12916	0.26469
H	−2.68353	2.50893	0.73548
H	−4.26675	0.60934	0.89849
H	−3.42252	−1.72807	0.63859
H	1.51590	0.41703	0.53509
H	3.46695	−2.11725	−0.58709
H	3.30810	−0.73774	−1.68685
H	3.44514	−0.45640	0.06646
H	0.95902	1.83766	−1.51021
H	2.45440	3.80274	−1.62089
H	2.32384	5.58057	0.12313
H	0.66297	5.36925	1.97125
H	−0.82083	3.39560	2.08890
C	−1.49679	0.01665	3.89054
C	−0.31165	0.73761	3.76415
C	0.90473	0.08629	3.47966
C	0.92763	−1.30485	3.34378
C	−0.26866	−2.03589	3.47337
C	−1.47628	−1.39306	3.73082
I	2.61910	1.34175	3.24297
H	−0.32469	1.81442	3.86367
H	−0.24738	−3.10962	3.34572

O	−2.68440	−2.02076	3.83140
C	−2.72398	−3.43861	3.59345
H	−3.77511	−3.71981	3.67620
H	−2.13941	−3.98086	4.34564
H	−2.35730	−3.68092	2.58977
I	2.67811	−2.45129	2.92258
O	−2.71937	0.56503	4.15527
C	−2.79346	1.99072	4.31819
H	−2.49569	2.50628	3.39971
H	−3.84149	2.20679	4.53220
H	−2.17101	2.32548	5.15601

E (UB3LYP) = −14971.4696540
H (Sum of electronic and thermal enthalpies) = −14971.058711
G (Sum of electronic and thermal free energies) = −14971.150673

Table S6. Optimized coordinates of [**4a**]$^{+}$···**5a**.

C	−1.03441	−1.01349	0.38124
C	−0.17653	0.08392	0.18827
C	−0.65634	1.41022	0.39178
C	−1.98393	1.57855	0.81212
C	−2.83155	0.48656	1.01359
C	−2.35107	−0.81367	0.78965
N	1.18433	−0.10496	−0.11758
C	1.77479	−1.14327	−0.79143
C	3.27568	−1.02339	−0.93244
O	1.13819	−2.08761	−1.28809
C	0.19283	2.61219	0.19012
C	0.98131	2.77568	−0.96718
C	1.74603	3.93251	−1.15143
C	1.73759	4.94754	−0.18398
C	0.95169	4.79847	0.96753
C	0.18475	3.64410	1.15081
H	−0.65346	−2.01615	0.24940
H	−2.36147	2.58454	0.96359
H	−3.85365	0.64397	1.34357
H	−2.99767	−1.66993	0.95483
H	1.80987	0.63761	0.17583
H	3.71127	−2.02384	−0.95459
H	3.49418	−0.53078	−1.88707
H	3.72769	−0.43932	−0.12729
H	0.97851	2.00815	−1.73459
H	2.33975	4.04662	−2.05414
H	2.33315	5.84464	−0.32775
H	0.94195	5.57828	1.72397
H	−0.40606	3.53012	2.05429
C	−1.84447	−0.16568	4.02579
C	−0.67852	0.57794	3.81461
C	0.50784	−0.05131	3.44153
C	0.53805	−1.48263	3.27580
C	−0.60823	−2.23416	3.52611
C	−1.80465	−1.60700	3.89091
I	2.20831	1.17821	3.22338
H	−0.70278	1.65230	3.93311
H	−0.58363	−3.30760	3.39741

O	−2.95828	−2.22959	4.12344
C	−3.02222	−3.67303	3.99590
H	−4.05300	−3.93231	4.23545
H	−2.33709	−4.14512	4.70582
H	−2.78475	−3.97155	2.97077
I	2.23901	−2.54259	2.62246
O	−3.02605	0.34020	4.37580
C	−3.17504	1.77849	4.46640
H	−2.93758	2.24129	3.50448
H	−4.22345	1.93984	4.71532
H	−2.53263	2.17650	5.25740

E (UB3LYP) = −14971.2663257
H (Sum of electronic and thermal enthalpies) = −14970.855765
G (Sum of electronic and thermal free energies) = −14970.948018

Table S7. Optimized coordinates of heteroleptic complex **A**.

C	−1.52406	−0.07688	0.41201
C	−0.43487	0.80731	0.37178
C	−0.64252	2.19698	0.53657
C	−1.94724	2.65304	0.79088
C	−3.03356	1.77383	0.82301
C	−2.81813	0.40545	0.61763
N	0.89319	0.31649	0.26246
C	1.30750	−0.76074	−0.46119
C	2.78744	−1.06888	−0.37744
O	0.54751	−1.44963	−1.17189
C	0.46660	3.18424	0.42971
C	1.30715	3.20368	−0.69873
C	2.33038	4.15115	−0.80666
C	2.54063	5.08491	0.21802
C	1.70487	5.07467	1.34233
C	0.67042	4.13807	1.44129
H	−1.35101	−1.14053	0.31745
H	−2.10965	3.71743	0.93238
H	−4.03510	2.15240	1.00556
H	−3.64834	−0.29328	0.65447
H	1.62015	0.84458	0.74757
H	3.27480	−0.59415	0.47555
H	2.91655	−2.15257	−0.32722
H	3.26559	−0.71651	−1.29763
H	1.15769	2.47728	−1.49180
H	2.97064	4.15566	−1.68423
H	3.34913	5.80650	0.14243
H	1.86548	5.78677	2.14701
H	0.03430	4.12863	2.31971
C	−1.54107	−0.30626	3.71449
C	−1.63970	0.99816	4.20093
C	−0.49337	1.78499	4.39321
C	0.76690	1.23950	4.10291
C	0.87596	−0.08316	3.62650
C	−0.26053	−0.85351	3.41533
I	−0.80295	3.73476	5.19792
H	−2.61197	1.41349	4.42839
H	1.85399	−0.48621	3.40116

O	−0.25984	−2.12338	2.92498
C	1.01401	−2.74685	2.67706
H	0.78251	−3.73843	2.28588
H	1.58818	−2.83903	3.60597
H	1.59144	−2.18767	1.93581
C	4.21789	2.25195	1.48871
O	4.86173	3.01060	2.24717
O	3.11297	1.64427	1.79453
C	4.75168	1.93195	0.09263
H	5.43512	1.08124	0.18909
H	3.94918	1.66526	−0.59316
H	5.30846	2.79176	−0.28408
I	2.57554	2.29695	4.34124
O	−2.59814	−1.12887	3.50265
C	−3.91507	−0.63906	3.82113
H	−3.99653	−0.41447	4.89040
H	−4.15782	0.24883	3.22940
H	−4.59619	−1.45075	3.56223

E (UB3LYP) = −15199.9827400
H (Sum of electronic and thermal enthalpies) = −15199.516094
G (Sum of electronic and thermal free energies) = −15199.621187

Table S8. Optimized coordinates of [**4a**]OAc_2.

C	−1.52386	−0.41125	−1.03252
C	−0.46535	−1.12589	−0.47874
C	0.11359	−0.70719	0.73325
C	−0.35963	0.42169	1.40904
C	−1.43318	1.13730	0.84984
C	−2.01652	0.73790	−0.35397
I	1.72894	−1.89047	1.43598
H	−0.08240	−2.00474	−0.98024
H	−1.80889	2.01166	1.36473
O	−3.05477	1.37598	−0.95618
C	−3.60302	2.54113	−0.30894
H	−4.41476	2.87685	−0.95599
H	−2.84929	3.33149	−0.22268
H	−4.00051	2.28811	0.68009
O	0.16963	−2.68089	2.80963
C	0.65817	−3.53865	3.68901
O	1.85153	−3.88502	3.70980
C	−0.37578	−4.05510	4.66459
H	−0.81522	−3.21207	5.20604
H	−1.17878	−4.55290	4.11276
H	0.08558	−4.75215	5.36492
O	2.83966	−0.74640	−0.12269
C	4.11953	−1.07206	−0.18276
O	4.63765	−1.93445	0.54715
C	4.89104	−0.29206	−1.22456
H	4.83605	0.77538	−0.99021
H	5.93152	−0.61815	−1.24184
H	4.43231	−0.44242	−2.20636
I	0.42238	1.15045	3.25355
O	−2.15136	−0.72795	−2.20067
C	−1.67973	−1.88074	−2.92377
H	−0.63179	−1.75651	−3.21928
H	−1.79815	−2.79280	−2.32792
H	−2.30669	−1.94180	−3.81461

E (UB3LYP) = −14756.9973745
H (Sum of electronic and thermal enthalpies) = −14756.726480
G (Sum of electronic and thermal free energies) = −14756.812124

Table S9. Optimized coordinates of ([**4a**]OAc)$^+$.

C	−1.28899	−0.83277	−0.21187
C	−0.01665	−1.37670	−0.13708
C	0.93997	−0.83089	0.75406
C	0.63067	0.27866	1.57399
C	−0.65337	0.82222	1.48976
C	−1.61585	0.28145	0.62255
I	2.76298	−1.81262	0.82455
H	0.25771	−2.21956	−0.75756
H	−0.90906	1.67796	2.10031
O	−2.87097	0.74400	0.49963
C	−3.29317	1.85776	1.32114
H	−4.33482	2.03144	1.05001
H	−2.69644	2.74761	1.09866
H	−3.22103	1.60066	2.38259
O	3.66723	−0.39669	−0.46575
C	4.96315	−0.70565	−0.64067
O	5.46542	−1.70267	−0.12198
C	5.67873	0.30034	−1.50546
H	5.13471	0.43592	−2.44341
H	5.69913	1.25970	−0.97978
H	6.69344	−0.05049	−1.69247
I	2.00721	1.22417	2.89141
O	−2.28428	−1.26951	−1.02510
C	−2.00277	−2.38806	−1.89088
H	−1.18889	−2.14877	−2.58419
H	−1.75184	−3.27954	−1.30532
H	−2.92336	−2.56180	−2.44933

E (UB3LYP) = −14528.243776
H (Sum of electronic and thermal enthalpies) = −14528.029120
G (Sum of electronic and thermal free energies) = −14528.099137

Table S10. Optimized coordinates of **7**.

C	−2.87255	0.01674	0.41044
C	−1.52704	−0.09138	0.01259
C	−0.82055	1.08974	−0.22849
C	−1.40922	2.35669	−0.06822
C	−2.74027	2.45237	0.33281
C	−3.48271	1.26172	0.57183
H	−3.43992	−0.88604	0.59431
H	−0.83336	3.25177	−0.26255
O	−4.77418	1.44179	0.94959
C	−5.58048	0.27093	1.19218
H	−6.56592	0.65023	1.46590
H	−5.65865	−0.34304	0.28823
H	−5.17002	−0.32106	2.01749
I	1.23422	1.11587	−0.82683
H	0.96997	0.62077	−2.37000
I	−0.74081	−2.05778	−0.19956
O	−3.41571	3.61968	0.51391
C	−2.70767	4.85153	0.27660
H	−1.85657	4.95095	0.95987
H	−2.36732	4.91335	−0.76315
H	−3.43023	5.64479	0.47323

E (UB3LYP) = −14300.3131745
H (Sum of electronic and thermal enthalpies) = −14300.147308
G (Sum of electronic and thermal free energies) = −14300.204691

Table S11. Optimized coordinates of **8**.

C	−0.54172	−0.31243	3.36244
C	0.32208	0.75821	3.60507
C	1.36969	0.64909	4.53337
C	1.54162	−0.56172	5.23381
C	0.66212	−1.64308	4.99842
C	−0.36451	−1.53808	4.07028
I	2.60580	2.36739	4.77014
H	0.18665	1.68697	3.06742
H	0.81299	−2.56998	5.53456
O	−1.24203	−2.53137	3.76318
C	−1.08612	−3.79524	4.43838
H	−1.87346	−4.43645	4.03949
H	−1.21781	−3.67951	5.51998
H	−0.10659	−4.23555	4.22187
I	3.08705	−0.91610	6.61823
O	−1.56886	−0.28865	2.47956
C	−1.80157	0.92250	1.73068
H	−0.93500	1.16666	1.10710
H	−2.66330	0.70837	1.09724
H	−2.03378	1.75608	2.40188
O	4.55235	−2.06443	4.53204
C	3.97629	−1.70602	3.34551
H	3.07455	−1.07837	3.46827
C	4.94320	−0.84216	2.49722
C	3.47253	−2.95532	2.57839
F	4.37443	−0.39527	1.34464
F	5.31587	0.24932	3.20940
F	6.07868	−1.50513	2.15595
F	4.47555	−3.81200	2.25487
F	2.82526	−2.63675	1.42545
F	2.59598	−3.64034	3.35348

E (UB3LYP) = −15089.368945
H (Sum of electronic and thermal enthalpies) = −15089.150897
G (Sum of electronic and thermal free energies) = −15089.234970

Table S12. Optimized coordinates of **11**.

C	−0.52101	−0.26120	3.43070
C	0.27732	0.85471	3.67919
C	1.36399	0.78692	4.57294
C	1.61696	−0.42206	5.22133
C	0.81260	−1.55204	4.99718
C	−0.25028	−1.48628	4.10090
I	2.50819	2.56633	4.82598
H	0.07023	1.78780	3.17225
H	1.04527	−2.48245	5.49673
O	−1.07606	−2.52713	3.79786
C	−0.84908	−3.78099	4.46990
H	−1.60985	−4.46019	4.08215
H	−0.97219	−3.67039	5.55331
H	0.14644	−4.17582	4.24240
I	3.30758	−0.75172	6.49165
O	−1.57476	−0.28100	2.56916
C	−1.87398	0.92403	1.83840
H	−1.03011	1.21596	1.20344
H	−2.73482	0.67887	1.21453
H	−2.13409	1.74177	2.51955
O	3.96375	−2.23103	4.52688
C	3.75218	−1.81696	3.24500
H	2.92448	−1.09704	3.10799
C	4.99001	−1.07036	2.68427
C	3.36496	−3.01097	2.33780
F	4.82388	−0.60605	1.41132
F	5.24538	0.01910	3.46132
F	6.12071	−1.82704	2.68529
F	4.32532	−3.97337	2.28179
F	3.09006	−2.64858	1.05063
F	2.24014	−3.60631	2.81869
H	2.64745	0.24474	7.65749

E (UB3LYP) = −15089.953144
H (Sum of electronic and thermal enthalpies) = −15089.726983
G (Sum of electronic and thermal free energies) = −15089.810456

Table S13. Optimized coordinates of **12**.

C	−1.76333	0.29982	−1.08847
C	−0.38722	−0.00826	−1.02487
C	0.39149	0.32312	0.09710
C	−0.23115	0.98860	1.16027
C	−1.58863	1.33193	1.10217
C	−2.37015	0.97506	−0.03659
I	2.44360	−0.20175	0.32400
O	−3.68124	1.33132	0.00986
C	−4.51200	0.98042	−1.11480
H	−5.50662	1.35504	−0.86959
H	−4.55001	−0.10646	−1.24793
H	−4.15183	1.46163	−2.03092
H	0.34656	1.23830	2.04039
H	−2.33637	0.01113	−1.96009
I	0.42220	−0.92993	−2.72118
O	−2.25136	1.98952	2.07748
C	−1.52445	2.38933	3.26014
H	−1.13510	1.51347	3.78920
H	−0.70997	3.07368	3.00107
H	−2.25553	2.90382	3.88478
O	0.77264	−2.75616	−1.95859
H	1.59827	−2.65726	−1.43757

E (UB3LYP) = −14375.5449148
H (Sum of electronic and thermal enthalpies) = −14375.370930
G (Sum of electronic and thermal free energies) = −14375.430751

Table S14. Optimized coordinates of **TS$_{AB}$**.

C	−1.19699	−0.21174	0.14144
C	−0.10786	0.71644	0.21031
C	−0.35339	2.06643	0.68578
C	−1.62907	2.38068	1.16663
C	−2.66783	1.45272	1.11148
C	−2.44698	0.15407	0.58544
N	1.17516	0.33161	−0.03546
C	1.50919	−0.75335	−0.85631
C	2.83419	−1.42020	−0.58802
O	0.77852	−1.11457	−1.78612
C	0.66427	3.12231	0.59104
C	1.44557	3.27103	−0.57561
C	2.36772	4.31400	−0.67708
C	2.53890	5.21014	0.38750
C	1.76468	5.06969	1.55006
C	0.82216	4.04771	1.64507
H	−1.01219	−1.22351	−0.19258
H	−1.82653	3.38253	1.53182
H	−3.65709	1.73130	1.46029
H	−3.25713	−0.56749	0.57588
H	2.06425	0.81119	0.62514
H	3.35371	−1.01527	0.27894
H	2.64131	−2.48632	−0.43354
H	3.46209	−1.32313	−1.47858
H	1.31061	2.58688	−1.40619
H	2.95748	4.42596	−1.58187
H	3.27103	6.00917	0.31385
H	1.90730	5.75021	2.38418
H	0.23943	3.93168	2.55228
C	−1.73729	−0.11941	3.84193
C	−2.00586	1.13220	4.39342
C	−0.96304	2.03069	4.69002
C	0.36413	1.65155	4.46446
C	0.63560	0.39600	3.88487
C	−0.39439	−0.48020	3.55573
I	−1.55132	3.94588	5.42872
H	−3.02938	1.42805	4.57966
H	1.66219	0.12174	3.68561

O	−0.21742	−1.69570	2.95912
C	1.13148	−2.10324	2.66843
H	1.04287	−3.04594	2.12644
H	1.69846	−2.25975	3.59336
H	1.64261	−1.36508	2.04578
C	4.07116	1.72238	1.12449
O	4.87207	2.24279	1.93654
O	2.93539	1.21139	1.50245
C	4.46357	1.63032	−0.34378
H	5.15186	0.78465	−0.45292
H	3.60756	1.46875	−0.99943
H	4.99387	2.53710	−0.64076
I	2.06297	2.82358	4.99809
O	−2.68342	−1.05170	3.52951
C	−4.06129	−0.71341	3.76020
H	−4.24968	−0.53681	4.82536
H	−4.35466	0.16765	3.17918
H	−4.63524	−1.57922	3.42623

E (UB3LYP) = −15199.980258
H (Sum of electronic and thermal enthalpies) = −15199.519050
G (Sum of electronic and thermal free energies) = −15199.622514

E.2 Optimized Coordinates Associated with 4-Iodoanisole

Table S15. Optimized coordinates of **4b**.

C	−1.19449	0.12806	3.85481
C	−0.02149	0.87521	3.73697
C	1.16602	0.24192	3.33695
C	1.17662	−1.12732	3.05766
C	−0.00257	−1.87907	3.17591
C	−1.19236	−1.25096	3.57559
I	2.96522	1.38075	3.15469
H	−0.03839	1.93761	3.95561
H	0.02885	−2.93926	2.95472
O	−2.39688	−1.89097	3.72212
C	−2.44483	−3.30328	3.45038
H	−3.47980	−3.60182	3.62708
H	−1.78339	−3.85770	4.12626
H	−2.17593	−3.51385	2.40875
H	2.09064	−1.62310	2.74811
H	−2.12094	0.60328	4.16329

E (UB3LYP) = −7266.12689524
H (Sum of electronic and thermal enthalpies) = −7265.995111
G (Sum of electronic and thermal free energies) = −7266.039828

Table S16. Optimized coordinates of [**4b**]$^{+}$.

C	−1.73638	−0.15246	3.89283
C	−0.57175	0.52867	3.62914
C	0.59540	−0.19982	3.26100
C	0.56650	−1.62219	3.16477
C	−0.59743	−2.31134	3.42697
C	−1.76763	−1.58120	3.79543
I	2.35618	0.81437	2.85607
H	−0.54060	1.61005	3.70022
H	−0.61587	−3.39163	3.35177
O	−2.93754	−2.13630	4.06721
C	−3.10085	−3.58258	4.00322
H	−4.14430	−3.75671	4.26220
H	−2.43940	−4.06035	4.73009
H	−2.89265	−3.93334	2.98923
H	1.46388	−2.16254	2.88397
H	−2.64700	0.36576	4.17558

E (UB3LYP) = −7265.91443126
H (Sum of electronic and thermal enthalpies) = −7265.782195
G (Sum of electronic and thermal free energies) = −7265.827250

Table S17. Optimized coordinates of **4b**–OAc.

C	−1.94227	−0.49077	−0.75476
C	−0.90576	−1.25690	−0.23301
C	−0.16289	−0.76155	0.85622
C	−0.45862	0.49042	1.41525
C	−1.50113	1.25907	0.88974
C	−2.24741	0.76943	−0.19845
I	1.40148	−1.91524	1.65425
H	−0.67588	−2.22358	−0.66779
H	−1.71780	2.22418	1.33136
O	−3.28050	1.43638	−0.78373
C	−3.64373	2.72938	−0.25862
H	−4.47937	3.06866	−0.87279
H	−2.81078	3.43529	−0.34700
H	−3.96269	2.65075	0.78646
H	0.11578	0.87227	2.25243
H	−2.52826	−0.85091	−1.59468
O	3.02316	−0.72490	−0.10490
C	4.23336	−1.17157	0.04779
O	4.60010	−2.02360	0.88827
C	5.25371	−0.55930	−0.91354
H	4.79354	0.13505	−1.61770
H	6.01047	−0.03816	−0.31900
H	5.74465	−1.37287	−1.45578

E(UB3LYP) = −7494.6318773
H (Sum of electronic and thermal enthalpies) = −7494.444377
G (Sum of electronic and thermal free energies) = −7494.508901

Table S18. Optimized coordinates of [**4b**]$^{+}$···OAc^{-}.

C	−1.88397	−1.31514	−0.50176
C	−0.85801	−2.11623	0.07262
C	0.15253	−1.53024	0.88279
C	0.15397	−0.17129	1.11265
C	−0.86868	0.63765	0.52816
C	−1.89206	0.04073	−0.27387
H	0.93102	0.27452	1.72062
H	−2.64694	−1.76988	−1.12312
O	0.81780	1.01874	−1.67364
C	1.34393	−0.12865	−1.88434
O	0.78286	−1.07188	−2.52902
C	2.75440	−0.37459	−1.34535
H	2.97351	0.27285	−0.49301
H	2.88801	−1.42311	−1.06690
H	3.47115	−0.14425	−2.14294
I	−0.84211	−4.17240	−0.26641
O	−0.97544	1.94538	0.70173
C	0.05751	2.66934	1.42477
H	0.08919	2.33664	2.46562
H	1.02222	2.52149	0.93317
H	−0.24243	3.71515	1.37015
H	0.93025	−2.15132	1.31269
H	−2.65124	0.68471	−0.70463

E (UB3LYP) = −7494.630251
H (Sum of electronic and thermal enthalpies) = −7494.442619
G (Sum of electronic and thermal free energies) = −7494.504966

Table S19. Optimized coordinates of **4b**···**5a**.

C	−1.64481	−1.11503	0.25785
C	−0.70897	−0.06872	0.20333
C	−1.13346	1.27353	0.37568
C	−2.49509	1.51443	0.62505
C	−3.42676	0.47364	0.67941
C	−2.99542	−0.84404	0.48854
N	0.67825	−0.32392	0.07837
C	1.31249	−1.42917	−0.40227
C	2.82233	−1.40254	−0.29379
O	0.72215	−2.40695	−0.90369
C	−0.19123	2.42728	0.33336
C	0.74046	2.58108	−0.71222
C	1.60738	3.67966	−0.74270
C	1.55715	4.64798	0.26945
C	0.62560	4.51138	1.30761
C	−0.24071	3.41371	1.33715
H	−1.30844	−2.13572	0.14634
H	−2.82741	2.53934	0.75913
H	−4.47431	0.68967	0.86784
H	−3.70240	−1.66697	0.54092
H	1.28534	0.39960	0.44623
H	3.13431	−2.21609	0.36876
H	3.24503	−1.59825	−1.28349
H	3.21428	−0.45912	0.09212
H	0.78003	1.84660	−1.51067
H	2.31717	3.78223	−1.55910
H	2.23433	5.49729	0.24926
H	0.58115	5.25370	2.09978
H	−0.94697	3.30797	2.15439
C	−1.18442	0.11833	3.90362
C	−0.01891	0.86996	3.75325
C	1.16282	0.23899	3.33910
C	1.18613	−1.13829	3.10900
C	0.01489	−1.89385	3.25479
C	−1.17944	−1.26098	3.63307
I	2.90944	1.40718	2.96194
H	−0.04200	1.93793	3.93610
H	0.04791	−2.95531	3.04254

O	−2.38703	−1.89695	3.75752
C	−2.44985	−3.29018	3.40690
H	−3.49648	−3.57687	3.52390
H	−1.82798	−3.89472	4.07745
H	−2.14083	−3.45022	2.36810
H	2.09630	−1.63204	2.78684
H	−2.11424	0.59655	4.19480

E (UB3LYP) = −7937.6519551
H (Sum of electronic and thermal enthalpies) = −7937.267293
G (Sum of electronic and thermal free energies) = −7937.350122

Table S20. Optimized coordinates of [**4b**]⁺···**5a**.

C	−1.06298	−1.06139	0.27094
C	−0.23751	0.07483	0.12458
C	−0.78111	1.38756	0.35119
C	−2.11605	1.49630	0.75533
C	−2.92031	0.36534	0.91675
C	−2.38594	−0.91413	0.66378
N	1.11126	−0.01827	−0.20374
C	1.93070	−1.13246	−0.29743
C	3.35838	−0.82664	−0.68171
O	1.53136	−2.28765	−0.10887
C	0.03611	2.61695	0.21224
C	0.74874	2.90158	−0.97091
C	1.50350	4.07457	−1.08006
C	1.55965	4.97968	−0.01093
C	0.84452	4.71031	1.16491
C	0.08440	3.54277	1.27443
H	−0.64946	−2.04350	0.10147
H	−2.52935	2.48327	0.93554
H	−3.95454	0.47245	1.22809
H	−3.00969	−1.79440	0.78255
H	1.59914	0.86613	−0.29893
H	4.02275	−1.48056	−0.11315
H	3.47894	−1.05908	−1.74593
H	3.63312	0.21697	−0.51634
H	0.68711	2.22383	−1.81786
H	2.03769	4.28693	−2.00182
H	2.14953	5.88789	−0.09506
H	0.88261	5.40834	1.99637
H	−0.46099	3.33555	2.18942
C	−1.74710	−0.19449	3.88154
C	−0.55374	0.45688	3.62564
C	0.58439	−0.29547	3.26876
C	0.51737	−1.71008	3.19492
C	−0.67268	−2.36748	3.46311
C	−1.82172	−1.61163	3.79897
I	2.40717	0.67616	2.92224
H	−0.50223	1.53688	3.69002
H	−0.71244	−3.44703	3.39208

O	−3.02711	−2.14074	4.06264
C	−3.19835	−3.57676	3.99090
H	−4.24615	−3.75243	4.23503
H	−2.55360	−4.07418	4.72151
H	−2.98280	−3.93657	2.98025
H	1.39614	−2.28234	2.91968
H	−2.64099	0.35826	4.14942

E (UB3LYP) = −7937.4438916
H (Sum of electronic and thermal enthalpies) = −7937.059299
G (Sum of electronic and thermal free energies) = −7937.141183

Table S21. Optimized coordinates of heteroleptic complex $\mathbf{A_{4b}}$.

C	−1.26474	−0.63366	0.28637
C	−0.32529	0.40983	0.35211
C	−0.77091	1.74084	0.54615
C	−2.14407	1.97437	0.71945
C	−3.07718	0.93429	0.65933
C	−2.62923	−0.37071	0.42809
N	1.07039	0.15906	0.32704
C	1.71634	−0.94448	−0.14715
C	3.21555	−0.96778	0.06148
O	1.14487	−1.88423	−0.73797
C	0.16031	2.90307	0.56292
C	0.99135	3.17091	−0.54000
C	1.83310	4.28815	−0.54112
C	1.86913	5.14583	0.56714
C	1.04504	4.88528	1.66989
C	0.19011	3.77743	1.66360
H	−0.92524	−1.64978	0.15145
H	−2.48093	2.99439	0.87959
H	−4.13563	1.14155	0.78721
H	−3.33485	−1.19541	0.38681
H	1.66389	0.86324	0.76572
H	3.56733	−0.20905	0.75985
H	3.49565	−1.96185	0.42026
H	3.70071	−0.81496	−0.90851
H	0.97482	2.50409	−1.39717
H	2.46709	4.48564	−1.40079
H	2.53696	6.00253	0.57361
H	1.07489	5.53906	2.53705
H	−0.44215	3.57457	2.52239
C	−1.32979	0.09505	3.87959
C	−0.32122	1.02335	4.10560
C	1.02294	0.62995	3.96199
C	1.35087	−0.67893	3.58079
C	0.33549	−1.60421	3.33220
C	−1.00987	−1.22079	3.48655
I	2.55388	2.01198	4.35569
H	−0.57452	2.03881	4.38853
H	0.60155	−2.60482	3.01508

O	−2.06984	−2.04752	3.28096
C	−1.81051	−3.38140	2.80206
H	−2.79184	−3.83643	2.65991
H	−1.24059	−3.95698	3.53982
H	−1.27381	−3.35766	1.84809
H	2.38662	−0.97778	3.46091
H	−2.37316	0.37656	3.97462
C	4.11488	2.64660	1.47271
O	4.67633	3.43662	2.26342
O	3.09764	1.89305	1.75724
C	4.65335	2.46486	0.05408
H	3.89945	2.05535	−0.61751
H	5.01779	3.42438	−0.31825
H	5.49641	1.76726	0.11165

E (UB3LYP) = −8166.1649665
H (Sum of electronic and thermal enthalpies) = −8165.724600
G (Sum of electronic and thermal free energies) = −8165.819749

Table S22. Optimized coordinates of [**4b**]OAc$_2$.

C	−1.65167	−0.43672	−0.98221
C	−0.58148	−1.17479	−0.48440
C	0.10963	−0.69062	0.63655
C	−0.25033	0.50608	1.26040
C	−1.32793	1.24407	0.75767
C	−2.03023	0.77316	−0.36597
I	1.73426	−1.80438	1.39829
H	−0.29475	−2.10523	−0.96204
H	−1.60322	2.17080	1.24612
O	−3.09080	1.41551	−0.93278
C	−3.52681	2.65796	−0.34600
H	−4.37247	2.98571	−0.95280
H	−2.73140	3.41039	−0.38608
H	−3.85263	2.50863	0.68932
H	0.29088	0.87061	2.12670
H	−2.20647	−0.78324	−1.84854
O	0.15302	−2.68500	2.70622
C	0.61284	−3.59970	3.54269
O	1.80817	−3.93539	3.59933
C	−0.46067	−4.20184	4.42353
H	−0.96154	−3.40722	4.98432
H	−1.21114	−4.69034	3.79443
H	−0.01985	−4.92610	5.10947
O	2.89160	−0.62898	−0.10212
C	4.17851	−0.93210	−0.11460
O	4.68435	−1.78967	0.62931
C	4.97662	−0.12953	−1.11894
H	4.90221	0.93376	−0.87094
H	6.02076	−0.44399	−1.10409
H	4.55418	−0.26971	−2.11823

E (UB3LYP) = −7723.1845685
H (Sum of electronic and thermal enthalpies) = −7722.939956
G (Sum of electronic and thermal free energies) = −7723.014660

Table S23. Optimized coordinates of $\mathbf{7_{4b}}$.

C	−3.09902	−0.00022	0.30506
C	−1.75353	−0.10619	−0.06650
C	−1.00160	1.05956	−0.21517
C	−1.53928	2.33530	0.00428
C	−2.87996	2.43075	0.37056
C	−3.66452	1.26877	0.52314
H	−3.68229	−0.90548	0.41996
H	−1.31643	−1.08430	−0.23402
H	−0.93789	3.23063	−0.10540
H	−3.33415	3.40083	0.54646
O	−4.95974	1.47681	0.88782
C	−5.80871	0.32508	1.07032
H	−6.78248	0.72381	1.35853
H	−5.90411	−0.24209	0.13787
H	−5.42455	−0.32097	1.86742
I	1.05774	0.87875	−0.74567
H	0.84178	1.07396	−2.36245

E (UB3LYP) = −7266.50233285
H (Sum of electronic and thermal enthalpies) = −7266.362787
G (Sum of electronic and thermal free energies) = −7266.408999

Table S24. Optimized coordinates of **8_{4b}.**

C	−0.55130	−0.28224	3.36113
C	0.33871	0.77914	3.61185
C	1.37289	0.61084	4.53642
C	1.51991	−0.61402	5.20945
C	0.62865	−1.67856	4.96295
C	−0.39704	−1.50964	4.04169
H	0.24145	1.72833	3.09938
H	0.74512	−2.62710	5.47505
I	3.09339	−0.87473	6.57806
O	−1.58741	−0.22553	2.48198
C	−1.80171	1.00050	1.75242
H	−0.93391	1.23956	1.12835
H	−2.66993	0.81310	1.11888
H	−2.01586	1.82870	2.43639
O	4.60060	−1.99237	4.53828
C	3.98156	−1.73784	3.34543
H	3.02661	−1.19267	3.45429
C	4.85968	−0.81885	2.46266
C	3.58430	−3.05356	2.63061
F	4.30172	−0.54739	1.25242
F	5.03706	0.36923	3.09659
F	6.09253	−1.33783	2.23154
F	4.65671	−3.80418	2.26865
F	2.84606	−2.83421	1.50902
F	2.82826	−3.80933	3.46467
H	2.06358	1.42726	4.71931
H	−1.09053	−2.31841	3.83228

E (UB3LYP) = −8055.555051
H (Sum of electronic and thermal enthalpies) = −8055.363223
G (Sum of electronic and thermal free energies) = −8055.438996

Table S25. Optimized coordinates of **11_{4b}**.

C	−0.53877	−0.30276	3.45880
C	0.31253	0.77757	3.74533
C	1.38028	0.60321	4.63698
C	1.57247	−0.64161	5.23412
C	0.73640	−1.73090	4.95840
C	−0.32044	−1.55442	4.06769
H	0.16900	1.74710	3.28433
H	0.91016	−2.70094	5.41014
I	3.24964	−0.93490	6.51876
O	−1.59983	−0.24143	2.60120
C	−1.86037	1.00779	1.93206
H	−1.01156	1.30030	1.30388
H	−2.73454	0.82605	1.30464
H	−2.08501	1.80069	2.65412
O	4.09779	−2.22872	4.48975
C	3.79900	−1.88332	3.20620
H	2.81616	−1.39888	3.05641
C	4.81099	−0.84978	2.64758
C	3.73546	−3.14255	2.30653
F	4.52765	−0.43159	1.37919
F	4.79435	0.26436	3.43236
F	6.09404	−1.30241	2.63819
F	4.90005	−3.84625	2.28646
F	3.41362	−2.87311	1.00713
F	2.77422	−3.98526	2.77515
H	2.04788	1.43303	4.84130
H	−0.98324	−2.38066	3.83022
H	2.49022	−0.10269	7.75117

E (UB3LYP) = −8056.139077
H (Sum of electronic and thermal enthalpies) = −8055.939331
G (Sum of electronic and thermal free energies) = −8056.012321

Table S26. Optimized coordinates of **12_{4b}**.

C	–1.68495	0.27805	–1.08260
C	–0.29045	0.07023	–1.02153
C	0.47078	0.48473	0.08572
C	–0.16647	1.10953	1.15526
C	–1.56138	1.31888	1.10951
C	–2.31193	0.90106	–0.01418
H	0.42121	1.42626	2.00781
H	–2.26058	–0.04229	–1.94413
I	0.66518	–0.86283	–2.61782
O	–2.27190	1.91453	2.09320
C	–1.57561	2.36325	3.27784
H	–1.09797	1.52040	3.78780
H	–0.83333	3.12686	3.02416
H	–2.34565	2.79507	3.91822
O	0.46229	–2.79483	–2.06241
H	1.22766	–2.99259	–1.48506
H	–3.38261	1.07788	–0.02504
H	1.54276	0.32131	0.11623

E (UB3LYP) = –7341.73366864
H (Sum of electronic and thermal enthalpies) = –7341.585965
G (Sum of electronic and thermal free energies) = –7341.634719

Table S27. Optimized coordinates of **E** (transition state between $\mathbf{A_{4b}}$ and **B**).

C	−0.91541	−0.31549	0.30163
C	0.07863	0.71288	0.22856
C	−0.29960	2.08054	0.51203
C	−1.60939	2.34091	0.91913
C	−2.55624	1.31678	1.01562
C	−2.20296	−0.01419	0.69293
N	1.39810	0.42439	0.02988
C	1.83758	−0.68877	−0.68886
C	3.27384	−1.09546	−0.47430
O	1.12270	−1.25755	−1.52651
C	0.64600	3.19848	0.34301
C	1.43110	3.31479	−0.82206
C	2.29833	4.39869	−0.98253
C	2.41169	5.36640	0.02439
C	1.63418	5.25634	1.18820
C	0.74535	4.19143	1.34008
H	−0.62620	−1.34168	0.12170
H	−1.90313	3.36224	1.13641
H	−3.56914	1.54629	1.33188
H	−2.93661	−0.80742	0.79346
H	2.21884	1.02920	0.72622
H	3.69174	−0.70484	0.45230
H	3.32679	−2.18640	−0.47989
H	3.86298	−0.72503	−1.32027
H	1.34653	2.56843	−1.60475
H	2.89337	4.48320	−1.88703
H	3.10174	6.19700	−0.09282
H	1.72989	5.99688	1.97704
H	0.15826	4.09919	2.24855
C	−2.21706	−0.51140	4.24031
C	−2.01089	0.87745	4.32450
C	−0.75199	1.41905	4.00835
C	0.28487	0.59409	3.58177
C	0.06853	−0.79211	3.48624
C	−1.17109	−1.34374	3.82524
C	4.01984	2.19210	1.36058
O	4.62537	2.87112	2.22111
O	2.91900	1.55175	1.64199

C	4.60717	2.05419	–0.03489
H	5.35348	1.25246	–0.00450
H	3.85420	1.79804	–0.77974
H	5.11245	2.97966	–0.31660
H	–0.60933	2.49302	4.06992
H	–1.34052	–2.41261	3.75247
I	1.63841	–2.05301	2.80614
H	1.23988	1.02775	3.30731
H	–3.17543	–0.95318	4.48499
O	–2.96983	1.77894	4.69688
C	–4.28384	1.27929	5.00623
H	–4.87912	2.15760	5.26180
H	–4.72705	0.77548	4.13963
H	–4.25400	0.59514	5.86180

E (UB3LYP) = –8166.162011
H (Sum of electronic and thermal enthalpies) = –8165.727668
G (Sum of electronic and thermal free energies) = –8165.823132

E.3 Optimized Coordinates Associated with Other Structures

Table S28. Optimized coordinates of [**4c**]+.

C	–2.49591	–0.69828	0.01447
C	–1.25449	–1.40669	0.00327
C	–0.06608	–0.70904	–0.00578
C	–0.07190	0.72458	–0.00781
C	–1.32042	1.41470	0.00788
C	–2.50935	0.72656	0.02072
I	1.63483	1.90621	–0.03036
H	–1.24823	–2.48847	0.00402
H	–1.32699	2.49906	0.00811
O	–3.67901	–1.28319	0.02014
C	–3.80157	–2.73773	–0.00910
H	–4.87393	–2.92565	–0.00729
H	–3.33573	–3.16702	0.88143
H	–3.34511	–3.12805	–0.92218
H	–3.46353	1.24232	0.03098
C	1.23134	–1.51343	–0.00798
F	1.99059	–1.23839	1.08445
F	1.98388	–1.24863	–1.10722
F	1.00117	–2.8465	–0.001

E (UB3LYP) = –7603.05924327
H (Sum of electronic and thermal enthalpies) = –7602.919410
G (Sum of electronic and thermal free energies) = –7602.974389

Table S29. Optimized coordinates of [**4d**]⁺.

C	−2.78352	−0.30513	0.16789
C	−1.59425	−1.00675	−0.04849
C	−0.33854	−0.40040	0.02090
C	−0.26509	1.01595	0.32228
C	−1.45383	1.72025	0.52639
C	−2.71127	1.11167	0.45979
I	1.33178	−1.61704	−0.30394
I	1.52798	2.08204	0.48377
H	−1.64030	−2.06303	−0.28040
H	−1.38913	2.77560	0.75901
C	−4.10516	−1.04725	0.02412
C	−3.94773	1.94358	0.77004
C	−5.22762	1.23286	0.28318
C	−5.26206	−0.24600	0.65538
H	−6.08991	1.74891	0.71725
H	−5.30889	1.34674	−0.80296
H	−5.22421	−0.35849	1.74394
H	−6.20574	−0.69356	0.32776
C	−3.98774	2.12799	2.31423
H	−3.09659	2.65658	2.66401
H	−4.87013	2.71972	2.57859
H	−4.04121	1.17082	2.83937
C	−3.88108	3.33552	0.10539
H	−3.09030	3.96553	0.52025
H	−3.72751	3.24904	−0.97489
H	−4.83211	3.85057	0.27434
C	−4.05043	−2.43771	0.69357
H	−3.36589	−3.12261	0.18652
H	−3.75096	−2.35799	1.74333
H	−5.04888	−2.88469	0.65622
C	−4.35068	−1.23766	−1.49982
H	−3.54299	−1.82034	−1.95176
H	−5.29214	−1.77795	−1.64275
H	−4.41589	−0.28139	−2.02510

E (UB3LYP) = −14384.0158382
H (Sum of electronic and thermal enthalpies) = −14383.713884
G (Sum of electronic and thermal free energies) = −14383.779318

Table S30. Optimized coordinates of $\mathbf{TS_{CD}}$.

C	0.24541	−2.10186	2.15480
C	−0.08731	−1.16275	1.12093
C	−1.17225	−0.20263	1.33793
C	−1.71533	−0.04031	2.63281
C	−1.30418	−0.89888	3.63174
C	−0.33445	−1.93116	3.38667
N	0.52502	−1.01200	−0.04660
C	1.50969	−1.87328	−0.56614
C	1.24728	−2.38084	−1.95383
O	2.53877	−2.07718	0.07881
C	−1.58084	0.43957	0.14404
C	−2.12008	1.75498	0.10081
C	−2.11546	2.43692	−1.09852
C	−1.62610	1.82630	−2.29642
C	−1.17995	0.51781	−2.30420
C	−1.21869	−0.22242	−1.09798
H	1.00733	−2.85214	1.98312
H	−2.49831	0.68882	2.81034
H	−1.74892	−0.82901	4.61993
H	−0.05057	−2.58303	4.20745
H	2.11246	−2.94568	−2.30248
H	0.35758	−3.02003	−1.94820
H	1.04600	−1.53704	−2.62153
H	−2.41048	2.25005	1.02114
H	−2.45194	3.46858	−1.13432
H	−1.65873	2.39145	−3.22350
H	−0.90013	0.02703	−3.23017
H	−1.35536	−1.29736	−1.16993

E (UB3LYP) = −670.626234
H (Sum of electronic and thermal enthalpies) = −670.389865
G (Sum of electronic and thermal free energies) = −670.442477

Table S31. Optimized coordinates of carbocation intermediate **D**.

C	−0.14233	−1.15689	1.20747
C	−1.23477	−0.27058	1.45966
C	−1.75871	−0.09295	2.76238
C	−1.18943	−0.81399	3.79656
C	−0.11080	−1.69926	3.53705
N	0.16889	−1.15358	−0.15778
C	1.04909	−2.05529	−0.77398
C	1.01354	−2.18309	−2.27440
O	1.83418	−2.71016	−0.08647
C	−1.59333	0.34215	0.24259
C	−2.43949	1.42952	−0.03713
C	−2.28566	2.07155	−1.25277
C	−1.28397	1.68125	−2.21238
C	−0.49471	0.59157	−2.00932
C	−0.81061	−0.29783	−0.84935
H	1.25045	−2.56508	2.10348
H	−2.58435	0.59067	2.92972
H	−1.56001	−0.70826	4.81109
H	0.32414	−2.25009	4.36633
H	1.46943	−3.14012	−2.53071
H	0.00368	−2.13482	−2.68736
H	1.61779	−1.38687	−2.72103
H	−3.10168	1.82402	0.72582
H	−2.88841	2.94941	−1.46944
H	−1.14990	2.28699	−3.10263
H	0.24176	0.29086	−2.74283
H	−1.55848	−0.98583	−1.31061

E (UB3LYP) = −670.677912
H (Sum of electronic and thermal enthalpies) = −670.438795
G (Sum of electronic and thermal free energies) = −670.490375

Table S32. Optimized coordinates of biarylacetamide **5a**.

C	–0.83917	–1.16190	0.02590
C	–0.06583	0.00760	–0.05862
C	–0.64705	1.26732	0.22945
C	–2.00763	1.31005	0.58141
C	–2.77541	0.14556	0.67869
C	–2.18351	–1.09373	0.40334
N	1.27708	–0.03476	–0.51490
C	2.20030	–1.02598	–0.33254
C	3.55427	–0.75928	–0.95452
O	1.96199	–2.08580	0.27734
C	0.12832	2.53956	0.17172
C	1.35031	2.68117	0.85928
C	2.04962	3.89348	0.82936
C	1.54238	4.98486	0.10977
C	0.32648	4.85464	–0.57557
C	–0.37601	3.64383	–0.54162
H	–0.39099	–2.11647	–0.21615
H	–2.45817	2.27204	0.80699
H	–3.82053	0.20492	0.96835
H	–2.76696	–2.00779	0.46769
H	1.62692	0.81763	–0.93708
H	3.74047	0.30251	–1.13425
H	4.32912	–1.16631	–0.30130
H	3.60384	–1.28889	–1.91264
H	1.74544	1.84700	1.43112
H	2.98592	3.98819	1.37268
H	2.08700	5.92460	0.08485
H	–0.07380	5.69428	–1.13743
H	–1.31374	3.54870	–1.08141

E (UB3LYP) = –671.5060984
H (Sum of electronic and thermal enthalpies) = –671.255605
G (Sum of electronic and thermal free energies) = –671.312377

Table S33. Optimized coordinates of aminyl radical **5a•** (intermediate **B**).

C	−0.58223	−1.13536	0.13476
C	0.16932	0.09166	0.09425
C	−0.54171	1.35169	0.21662
C	−1.93608	1.32025	0.31834
C	−2.64547	0.11111	0.33584
C	−1.95926	−1.12092	0.24911
N	1.48825	0.11648	−0.14479
C	2.30586	−0.99366	−0.07350
C	3.11976	−1.25988	−1.31599
O	2.43503	−1.65213	0.97408
C	0.17117	2.64937	0.25033
C	1.32279	2.83263	1.04373
C	1.95784	4.07747	1.09863
C	1.46212	5.15938	0.35650
C	0.31789	4.98789	−0.43547
C	−0.32692	3.74709	−0.48137
H	−0.05079	−2.07735	0.05103
H	−2.48056	2.25380	0.42025
H	−3.72744	0.12299	0.43003
H	−2.51425	−2.05427	0.26710
H	2.45561	−1.35677	−2.18185
H	3.78270	−0.40874	−1.50463
H	3.71007	−2.16919	−1.19072
H	1.70694	2.00646	1.63142
H	2.83730	4.20508	1.72389
H	1.96066	6.12389	0.39597
H	−0.06999	5.81879	−1.01843
H	−1.20489	3.62163	−1.10813

E (UB3LYP) = −670.8416481
H (Sum of electronic and thermal enthalpies) = −670.605527
G (Sum of electronic and thermal free energies) = −670.663979

Table S34. Optimized coordinates of cation **5a⁺** (intermediate **C**).

C	−0.07352	−2.48302	1.75278
C	−0.13822	−1.31914	0.86615
C	−1.03415	−0.17491	1.21607
C	−1.52305	−0.13509	2.53422
C	−1.33805	−1.21623	3.38380
C	−0.63832	−2.40915	2.98740
N	0.66806	−1.14671	−0.13054
C	1.57389	−2.11808	−0.59440
C	2.97485	−1.98995	−0.07274
O	1.19244	−2.91936	−1.44616
C	−1.37889	0.81819	0.23367
C	−1.70079	2.14634	0.64049
C	−2.01365	3.11091	−0.30566
C	−2.05771	2.77208	−1.67276
C	−1.77962	1.46155	−2.08957
C	−1.43921	0.49010	−1.15376
H	0.48647	−3.35496	1.43124
H	−2.13655	0.69380	2.86657
H	−1.77940	−1.18405	4.37635
H	−0.55965	−3.23445	3.68755
H	3.35004	−0.98050	−0.27055
H	2.97133	−2.13589	1.01299
H	3.61236	−2.73253	−0.55480
H	−1.64247	2.42350	1.68687
H	−2.22197	4.12964	0.00590
H	−2.32048	3.52859	−2.40664
H	−1.84567	1.19947	−3.14077
H	−1.27956	−0.53012	−1.47849

E (UB3LYP) = −670.638023
H (Sum of electronic and thermal enthalpies) = −670.400380
G (Sum of electronic and thermal free energies) = −670.400380

Table S35. Optimized coordinates of anion **5a**⁻ (Figure 6b).

C	−0.90382	−1.17180	−0.18923
C	−0.09120	−0.01653	−0.26368
C	−0.65595	1.22487	0.14568
C	−1.99821	1.26444	0.57233
C	−2.78895	0.11250	0.63222
C	−2.22841	−1.11714	0.25135
N	1.18386	−0.08978	−0.85056
C	2.16487	−0.67048	−0.17783
C	3.50232	−0.76828	−0.90104
O	2.11794	−1.12242	1.02950
C	0.13514	2.48606	0.13860
C	1.42921	2.53913	0.69513
C	2.15142	3.73753	0.71795
C	1.59878	4.91073	0.18199
C	0.31231	4.87150	−0.37301
C	−0.41154	3.67172	−0.39049
H	−0.47498	−2.12012	−0.50246
H	−2.41894	2.21584	0.88781
H	−3.81800	0.17024	0.97608
H	−2.82215	−2.02721	0.29472
H	3.44354	−0.40383	−1.92985
H	4.25209	−0.18172	−0.35638
H	3.84557	−1.80921	−0.90333
H	1.86015	1.64214	1.12651
H	3.14482	3.75839	1.15888
H	2.16233	5.83976	0.19708
H	−0.12583	5.77210	−0.79572
H	−1.40263	3.65097	−0.83524

E (UB3LYP) = −671.0072553
H (Sum of electronic and thermal enthalpies) = −670.770955
G (Sum of electronic and thermal free energies) = −670.826340

Table S36. Optimized coordinates of aminyl radical cation **5a**•+ (Figure 6b).

C	–0.76041	–1.19975	0.30806
C	–0.00922	–0.01482	0.06458
C	–0.65070	1.29478	0.16804
C	–2.04354	1.33150	0.35501
C	–2.76747	0.16251	0.55074
C	–2.11416	–1.10384	0.54192
N	1.28599	–0.04753	–0.36570
C	2.20881	–1.12465	–0.39919
C	3.53090	–0.74494	–1.00486
O	1.93238	–2.23283	0.04355
C	0.11536	2.54234	0.13073
C	1.35122	2.66680	0.81947
C	2.04511	3.87626	0.80914
C	1.53220	4.97683	0.10647
C	0.30606	4.86794	–0.57374
C	–0.40605	3.67226	–0.54892
H	–0.27320	–2.16174	0.25743
H	–2.54360	2.29076	0.42655
H	–3.83478	0.21230	0.74345
H	–2.69639	–2.00669	0.69790
H	1.66573	0.82993	–0.71529
H	3.98297	0.07214	–0.43139
H	4.19067	–1.61172	–0.99943
H	3.38403	–0.39099	–2.03136
H	1.73391	1.83760	1.40531
H	2.97728	3.96714	1.35832
H	2.07810	5.91567	0.09374
H	–0.08677	5.71909	–1.12179
H	–1.34374	3.59068	–1.08885

E (UB3LYP) = –671.2912837
H (Sum of electronic and thermal enthalpies) = –671.040867
G (Sum of electronic and thermal free energies) = –671.097517

Table S37. Optimized coordinates of carbazole **6a**.

C	−0.52188	−2.60937	1.80446
C	−0.75485	−1.59092	0.86987
C	−1.58540	−0.49432	1.19345
C	−2.19413	−0.39721	2.45100
C	−1.96454	−1.41012	3.38593
C	−1.13728	−2.50035	3.05922
N	−0.27868	−1.42312	−0.46760
C	0.57164	−2.33215	−1.09719
C	1.02139	−2.07223	−2.51051
O	0.95129	−3.34419	−0.49427
C	−1.63135	0.37767	0.03683
C	−2.30257	1.58849	−0.17267
C	−2.16876	2.22654	−1.40783
C	−1.37130	1.65064	−2.41209
C	−0.69452	0.43940	−2.21460
C	−0.82719	−0.20205	−0.97544
H	0.10878	−3.45451	1.57601
H	−2.83052	0.44972	2.68961
H	−2.42480	−1.35838	4.36854
H	−0.96660	−3.28207	3.79401
H	1.66951	−2.89827	−2.80416
H	0.16772	−2.02523	−3.19221
H	1.58021	−1.13486	−2.57910
H	−2.91431	2.01791	0.61505
H	−2.67921	3.16673	−1.59582
H	−1.27090	2.15101	−3.37084
H	−0.09840	0.04693	−3.02322

E (UB3LYP) = −670.2960373
H (Sum of electronic and thermal enthalpies) = −670.068311
G (Sum of electronic and thermal free energies) = −670.119656

Table S38. Optimized coordinates of hfip.

C	−0.11397	0.21841	0.40964
C	1.34236	0.71511	0.52307
C	−0.77041	0.56522	−0.94514
O	−0.10921	−1.17923	0.56993
F	−0.13220	−0.00573	−1.99453
F	−2.04718	0.10803	−0.94972
F	−0.81425	1.90180	−1.16417
F	1.82217	0.41860	1.75545
F	1.43657	2.05688	0.35965
F	2.16145	0.12848	−0.38275
H	−0.68589	0.74488	1.17857
H	−0.74004	−1.42176	1.26649

E (UB3LYP) = −790.09196861
H (Sum of electronic and thermal enthalpies) = −790.020534
G (Sum of electronic and thermal free energies) = −790.067593

Table S39. Optimized coordinates of hexafluoroisopropoxy radical (hfip•).

O	4.28242	−1.62920	3.48629
C	5.49796	−2.22310	3.31033
H	5.56080	−3.02876	4.07116
C	5.59777	−2.92850	1.92764
C	6.66834	−1.24687	3.62156
F	4.57228	−3.79672	1.79448
F	5.52529	−2.04170	0.90743
F	6.75287	−3.61871	1.79935
F	6.71877	−0.22594	2.73375
F	7.86442	−1.87662	3.59975
F	6.49410	−0.71857	4.85150

E (UB3LYP) = −789.405542
H (Sum of electronic and thermal enthalpies) = −789.348097
G (Sum of electronic and thermal free energies) = −789.394264

Table S40. Optimized coordinates of adduct of hfip and acetate anion (hfip–OAc⁻).

C	−0.07283	0.30668	0.41975
C	1.43103	0.63621	0.40959
C	−0.72859	0.50703	−0.96078
O	−0.25122	−1.01215	0.83336
F	−0.20819	−0.30380	−1.92006
F	−2.05301	0.22327	−0.87599
F	−0.62032	1.78527	−1.41252
F	1.93782	0.48194	1.65890
F	1.68379	1.91742	0.02964
F	2.14673	−0.17584	−0.41324
H	−0.55253	1.02624	1.09674
H	−0.86439	−1.00855	1.66658
O	−1.96847	1.19052	2.80224
C	−2.23626	0.05882	3.30081
O	−1.75996	−1.06177	2.87456
C	−3.18622	−0.03430	4.48944
H	−3.53072	0.95314	4.80307
H	−4.04763	−0.65338	4.21677
H	−2.67930	−0.52987	5.32405

E (UB3LYP) = −1018.8154409
H (Sum of electronic and thermal enthalpies) = −1018.689319
G (Sum of electronic and thermal free energies) = −1018.751335

Table S41. Optimized coordinates of acetate anion ($^{-}$OAc).

C	0.16332	−0.47553	−0.05207
O	1.32323	−0.63865	0.45721
O	−0.26128	0.60459	−0.58180
C	−0.78474	−1.68091	−0.00883
H	−1.04056	−1.89751	1.03477
H	−1.70377	−1.49596	−0.57033
H	−0.27920	−2.56539	−0.41038

E (UB3LYP) = −228.70028487
H (Sum of electronic and thermal enthalpies) = −228.646497
G (Sum of electronic and thermal free energies) = −228.679752

Table S42. Optimized coordinates of acetoxy radical (•OAc).

C	0.08396	−0.52078	0.00295
O	1.31042	−0.52158	−0.34845
O	−0.27210	0.66254	0.31938
C	−0.80100	−1.71246	0.01695
H	−1.58957	−1.58073	0.76195
H	−1.25798	−1.80582	−0.97491
H	−0.21134	−2.60956	0.22184

E (UB3LYP) = −228.48598488
H (Sum of electronic and thermal enthalpies) = −228.433076
G (Sum of electronic and thermal free energies) = −228.466807

Table S43. Optimized coordinates of acetic acid (AcOH).

C	0.09176	−0.46381	−0.08762
O	1.27405	−0.64405	0.55249
O	−0.18861	0.58673	−0.65916
C	−0.78854	−1.67873	−0.00445
H	−0.96061	−1.93207	1.04618
H	−1.73576	−1.48742	−0.50757
H	−0.27760	−2.52658	−0.47171
H	1.80155	0.17409	0.46480

E (UB3LYP) = −229.1705269
H (Sum of electronic and thermal enthalpies) = −229.103659
G (Sum of electronic and thermal free energies) = −229.136274

Table S44. Optimized coordinates of water (H_2O).

O	−0.38237	1.98437	0.00000
H	0.58715	2.02575	0.00000
H	−0.66698	2.91207	0.00000

E (UB3LYP) = −76.4694519
H (Sum of electronic and thermal enthalpies) = −76.444491
G (Sum of electronic and thermal free energies) = −76.466594

Table S45. Optimized coordinates of $H_3O \cdot PF_6$.

O	−0.63341	2.18112	−0.02743
H	−0.40725	1.83208	0.89312
H	0.18114	2.19122	−0.57588
H	−1.28391	1.57961	−0.44954
P	−0.49071	2.08304	3.76418
F	0.05369	0.77830	4.58474
F	−1.95376	1.36521	3.58725
F	0.00946	1.32570	2.31810
F	0.99099	2.77239	3.86926
F	−1.01321	3.35257	2.86249
F	−0.97081	2.81036	5.14242

E (UB3LYP) = −1017.8831021
H (Sum of electronic and thermal enthalpies) = −1017.816959
G (Sum of electronic and thermal free energies) = −1017.862248

Table S46. Optimized coordinates of hexafluorophosphate anion (PF_6^-).

P	0.17118	0.49215	0.00000
F	0.17118	0.49215	1.64815
F	–1.47697	0.49215	0.00000
F	0.17118	–1.15600	0.00000
F	1.81933	0.49215	0.00000
F	0.17118	0.49215	–1.64815
F	0.17118	2.14031	0.00000

E (UB3LYP) = –940.9894830
H (Sum of electronic and thermal enthalpies) = –940.964331
G (Sum of electronic and thermal free energies) = –941.000738

F. Additional Data

NMR analysis of the electrolysis and of 4a with [TMA]OAc in hfip

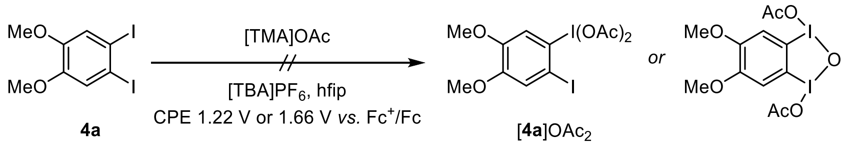

A 10-mL glass vial was charged with 1,2-diiodo-4,5-dimethoxybenzene (**4a**, 19.5 mg, 50.0 μmol, 1.00 equiv.), [TMA]OAc (26.5 mg, 0.200 mmol, 4.00 equiv.), [TBA]PF_6 (390 mg, 1.01 mmol, 20.2 equiv.), and hfip (5.0 mL). The reaction vessel was fitted with a glassy carbon anode, platinum cathode, and a Ag^+/Ag reference electrode. A constant potential of 1.40 V *vs.* Ag^+/Ag (1.22 *vs.* Fc^+/Fc) or 1.84 V *vs.* Ag^+/Ag (1.66 *vs.* Fc^+/Fc) was applied to the reaction mixture and stirred at 23 °C until ~13 C charge (~2.6 F/mol) is passed. Following electrolysis, 1,3,5-trimethoxybenzene was added to the reaction mixture and an aliquot was taken for ^{1}H NMR analysis, which indicated that 92% or 22% **4a** remained after the electrolysis at 1.22 *vs.* Fc^+/Fc or 1.66 *vs.* Fc^+/Fc, respectively. In both electrolyses, no I(III) species were observed by ^{1}H NMR.

NMR analysis of the electrolysis of 5a and 4a in hfip to probe the effect of [TMA]OAc

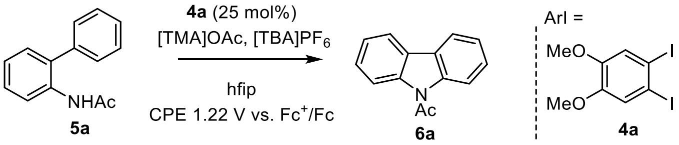

Electrolysis in the presence of [TMA]OAc. The 10-mL anodic cell of a divided cell was charged with *N*-biphenylacetamide (**5a**, 42.3 mg, 0.200 mmol, 1.00 equiv.), 1,2-diiodo-4,5-dimethoxybenzene (**4a**, 19.5 mg, 50.0 μmol, 0.250 equiv.), [TMA]OAc (53.0 mg, 0.400 mmol, 2.00 equiv.), [TBA]PF_6 (390 mg, 1.01 mmol, 5.02 equiv.), and hfip (5.0 mL). The cathodic cell was charged with [TBA]PF_6 (390 mg, 1.01 mmol, 5.02 equiv.) and hfip (5.0 mL). The anodic cell was fitted with a glassy carbon working electrode and a Ag^+/Ag reference electrode. The cathodic cell was fitted with a Pt counter electrode. A constant potential of 1.40 V *vs.* Ag^+/Ag (1.22 V *vs.* Fc^+/Fc) was applied to the reaction mixture and stirred at 23 °C until ~50 C charge (~2.6 F/mol) is passed. Following electrolysis, 1,3,5-trimethoxybenzene was added to the anodic cell and an aliquot was taken for ^{1}H NMR analysis, which indicated that product **6a** was obtained in 36% yield.

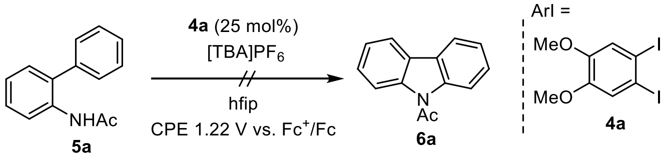

Electrolysis in the absence of [TMA]OAc. The 10-mL anodic cell of a divided cell was charged with *N*-biphenylacetamide (**5a**, 42.3 mg, 0.200 mmol, 1.00 equiv.), 1,2-diiodo-4,5-dimethoxybenzene (**4a**, 19.5 mg, 50.0 μmol, 0.250 equiv.), [TBA]PF_6 (390 mg, 1.01 mmol, 5.02 equiv.), and hfip (5.0 mL). The cathodic cell was charged with [TBA]PF_6 (390 mg, 1.01 mmol, 5.02 equiv.) and hfip (5.0 mL). The anodic cell was fitted with a glassy carbon working electrode and a Ag^+/Ag reference electrode, and the cathodic cell was fitted with a Pt counter electrode. A constant potential of 1.40 V *vs.* Ag^+/Ag (1.22 V *vs.* Fc^+/Fc) was applied to the reaction mixture and stirred at 23 °C until ~50 C charge (~2.6 F/mol) is passed. Following electrolysis, 1,3,5-trimethoxybenzene was added to the anodic cell and an aliquot was taken for ^{1}H NMR analysis, which indicated that product **6a** was not observed.

Table S47. Impact of the base on the electrocatalytic intramolecular C–H/N–H coupling using **4a** as catalyst. Reaction conditions: *N*-biphenylacetamide (**5a**, 0.200 mmol, 1.00 equiv.), 1,2-diiodoveratrole catalyst (**4a**, 50.0 μmol, 0.250 equiv.), [TMA]X salt (0.400 mmol, 2.00 equiv.), [TBA]PF_6 (390 mg, 1.01 mmol, 5.02 equiv.), hfip (5.0 mL), a glassy carbon working electrode, a Pt counter electrode, and a Ag^+/Ag reference electrode in an undivided cell setup. A constant potential of 1.40 V *vs.* Ag^+/Ag (1.22 V *vs.* Fc^+/Fc) was applied, and the reaction mixture was stirred at 23 °C until 50 C charge (~2.6 F/mol) was passed.

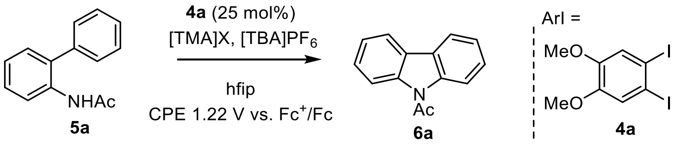

Entry	Counter anion X^-	pK_a of conjugate acid of X^-	[a]Yield of 6a
**1**	hydroxide	14.0	32
**2**	pivalate	5.03	68
**3**	**acetate**	**4.76**	**72**
**4**	p-methoxybenzoate	4.47	70
**5**	pentafluorobenzoate	1.75	58
**6**	4-pyridinecarboxylate	0.99	49
**7**	trifluoroacetate	0.30	13
**8**	triflate	−14.7	0

[a]Determined by 1H NMR using 1,3,5-trimethoxybenzene as internal standard.

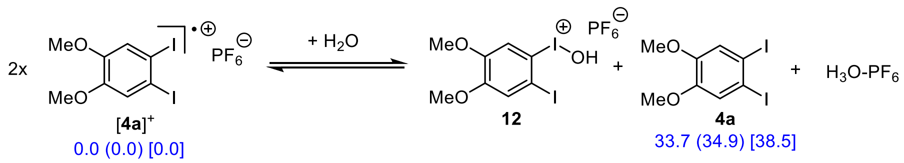

Figure S34. Computational evaluation of the disproportionation of [**4a**]$^+$ to generate **11** and **4a** in the presence of water.[36] The large energy penalty suggests that this mechanism is unlikely. This calculation was carried out using UB3LYP-D3/ DGDZVP2- DGDZVP (I)-SMD (2-methyl-1-propanol) level of theory, ΔE (ΔH) [ΔG].

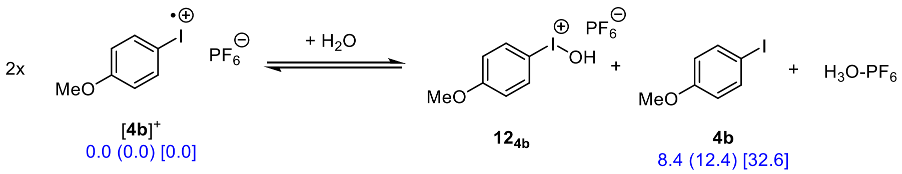

Figure S35. Computational evaluation of the disproportionation of [**4b**]$^+$ to generate **11$_{4b}$** and **4b** in the presence of water.[36] The large energy penalty suggests that this mechanism is unlikely. This calculation was carried out using UB3LYP-D3/ DGDZVP2- DGDZVP (I)-SMD (2-methyl-1-propanol) level of theory, ΔE (ΔH) [ΔG].

G. NMR Spectra of New Compounds

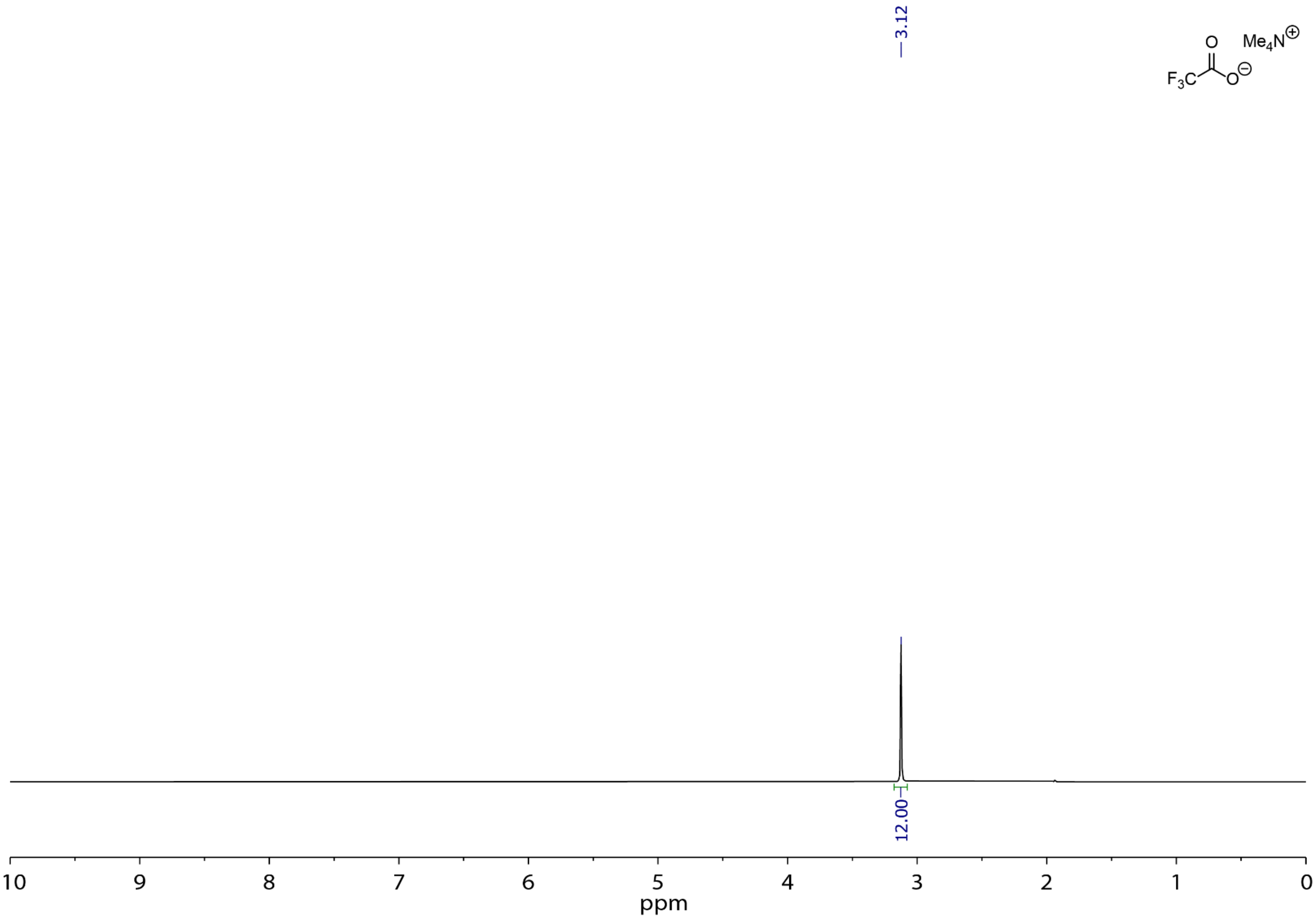

Figure S36. ^{1}H NMR spectrum of tetramethylammonium trifluoroacetate ([TMA]OTFA) in CD_3CN (400 MHz) at 23 °C.

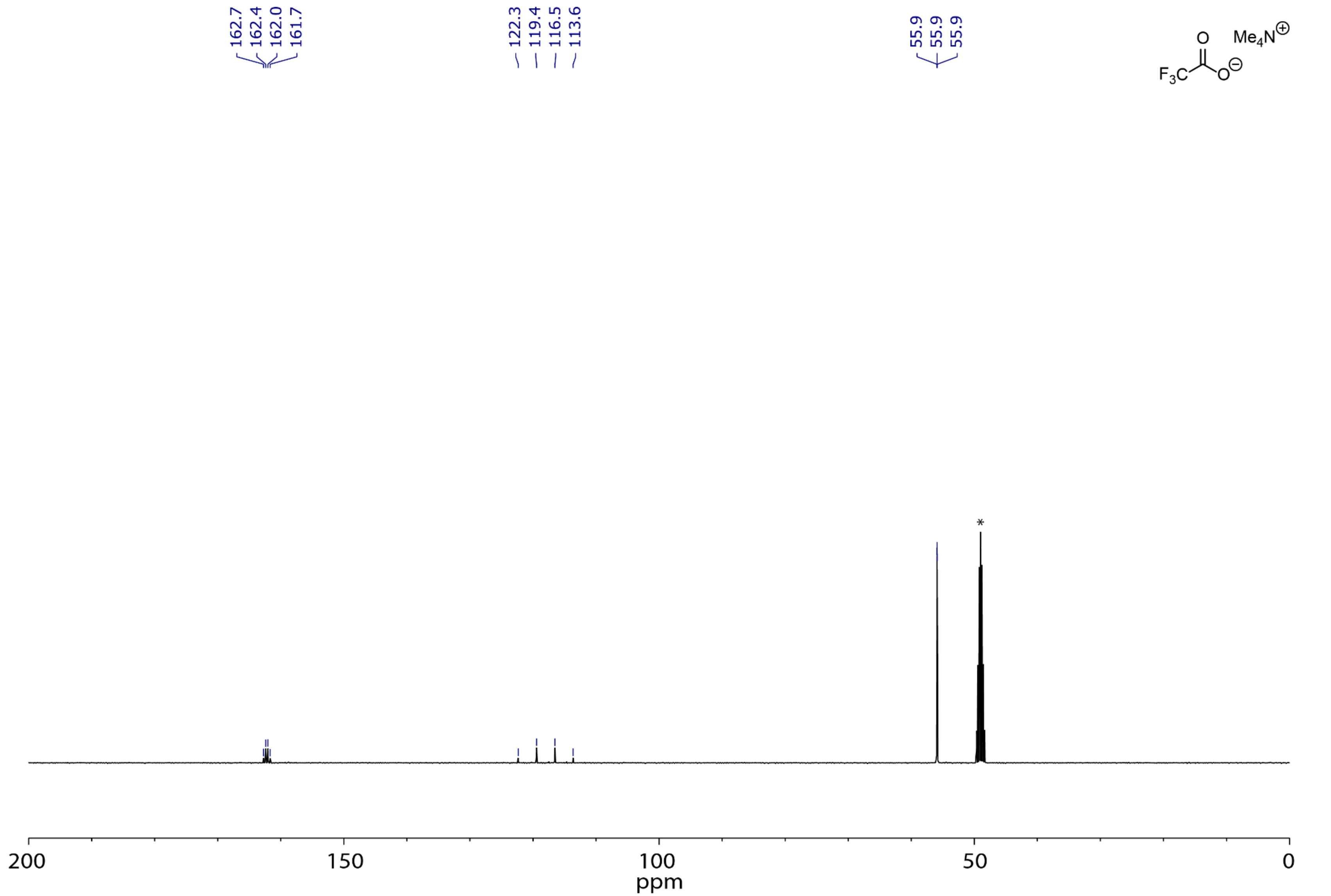

Figure S37. ^{13}C NMR spectrum of tetramethylammonium trifluoroacetate ([TMA]OTFA) in CD_3OD (400 MHz) at 23 °C. Solvent peak is marked with *.

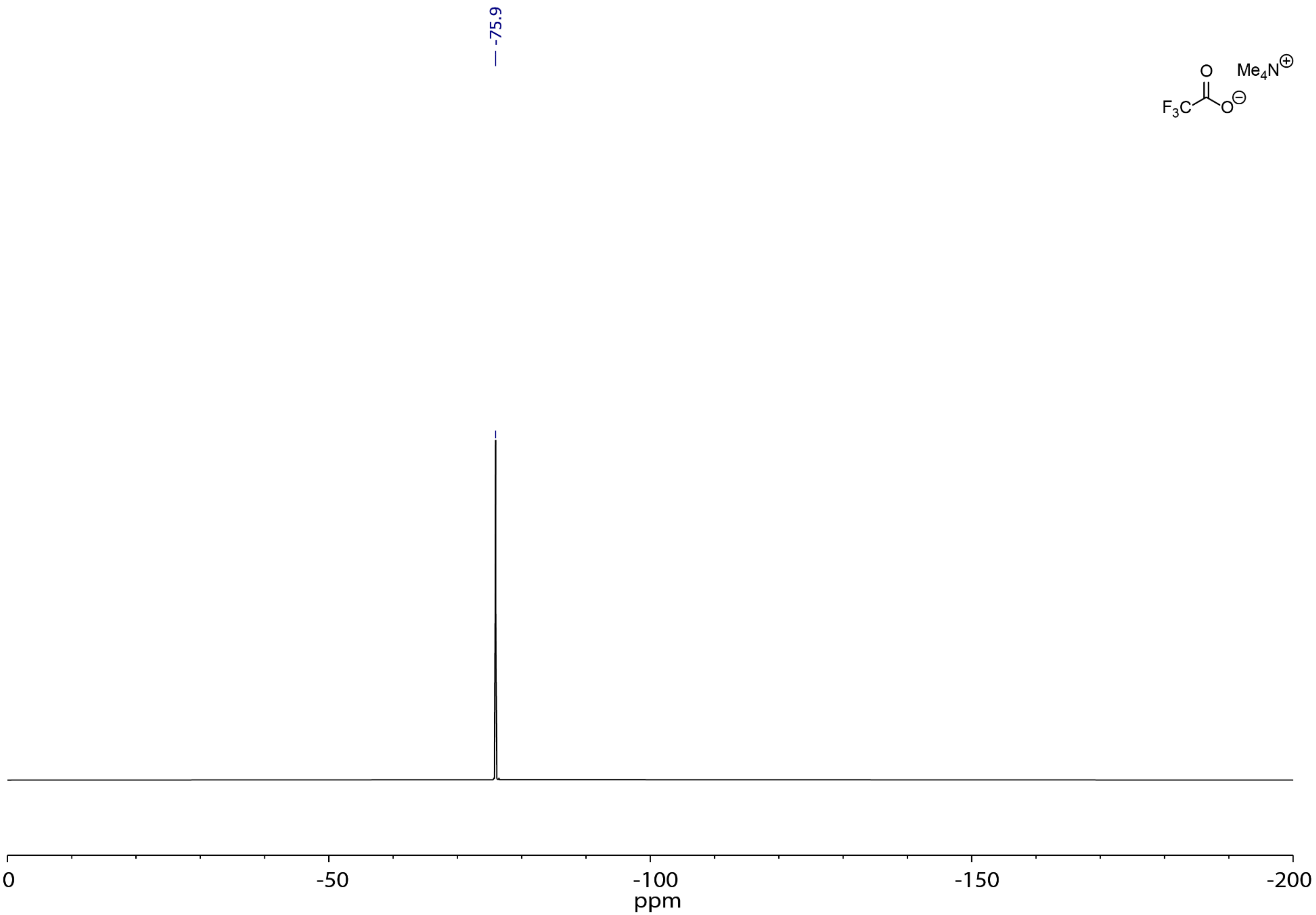

Figure S38. ^{19}F NMR spectrum of tetramethylammonium trifluoroacetate ([TMA]OTFA) in CD_3CN (400 MHz) at 23 °C.

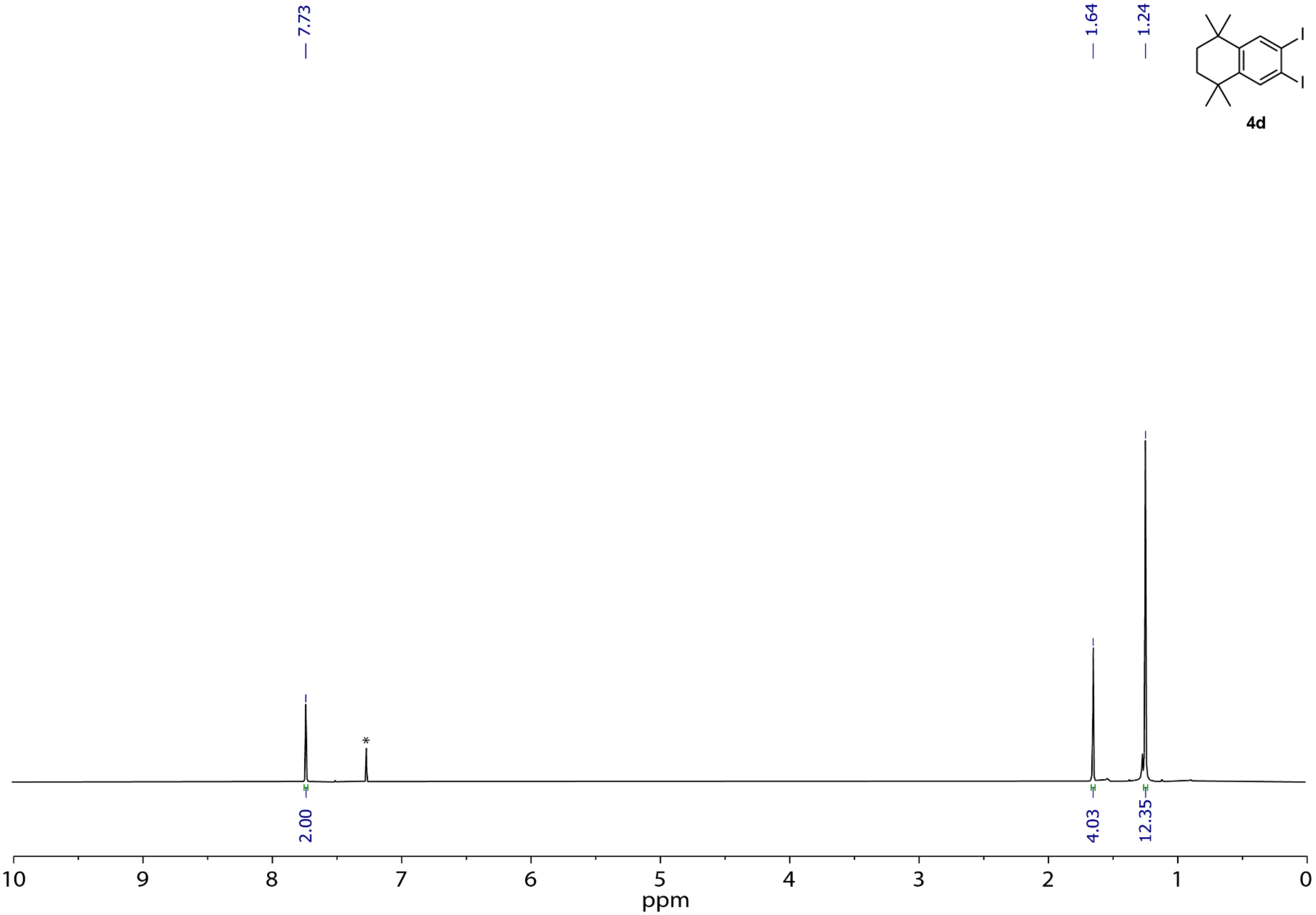

Figure S39. ^{1}H NMR spectrum of 6,7-diiodo-1,1,4,4-tetramethyl-1,2,3,4-tetrahydronaphthalene (**4d**) in CD_3Cl_3 (400 MHz) at 23 °C. Solvent peak is marked with *.

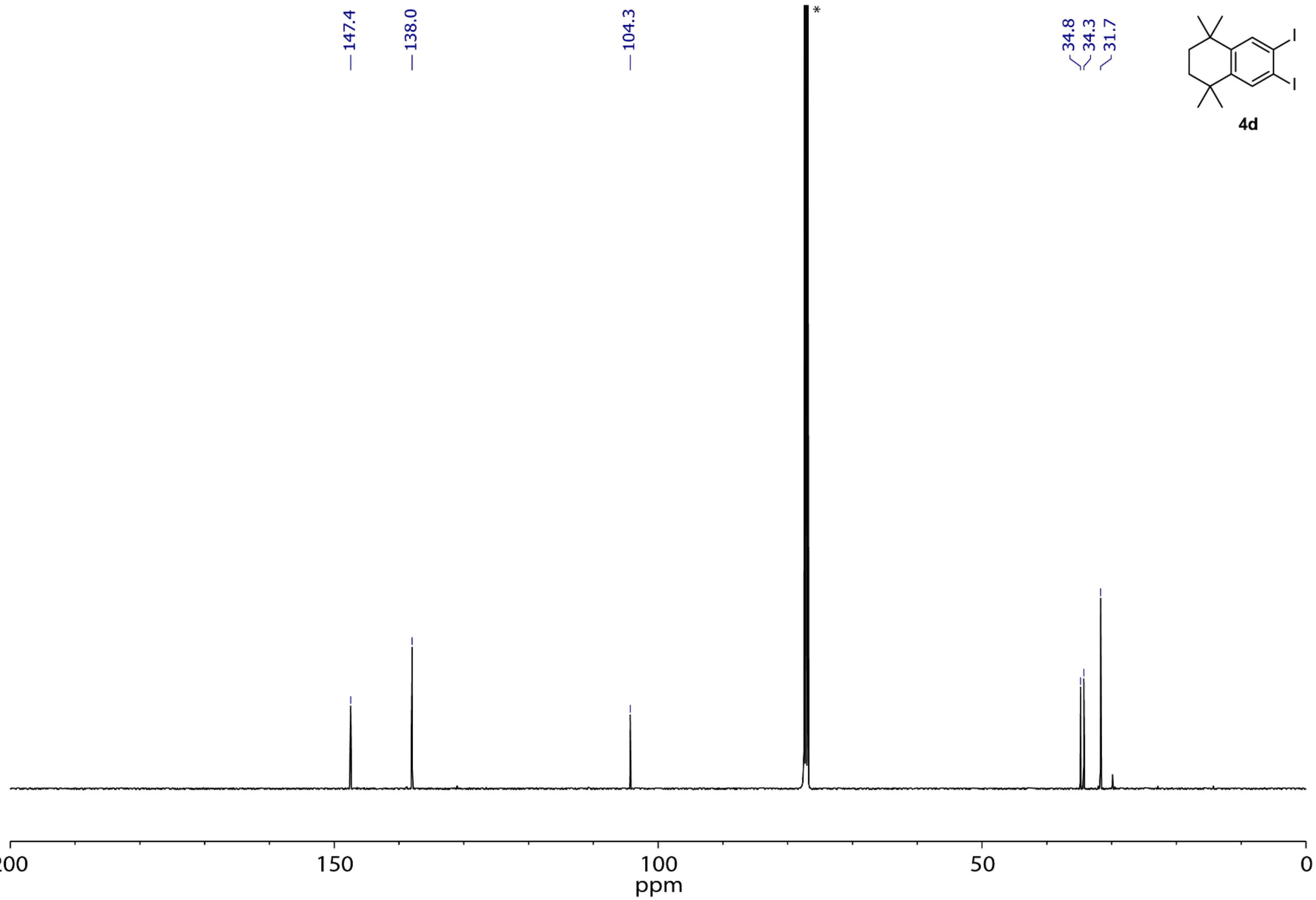

Figure S40. ^{13}C NMR spectrum of 6,7-Diiodo-1,1,4,4-tetramethyl-1,2,3,4-tetrahydronaphthalene (**4d**) in CD_3Cl_3 (400 MHz) at 23 °C. Solvent peak is marked with *.

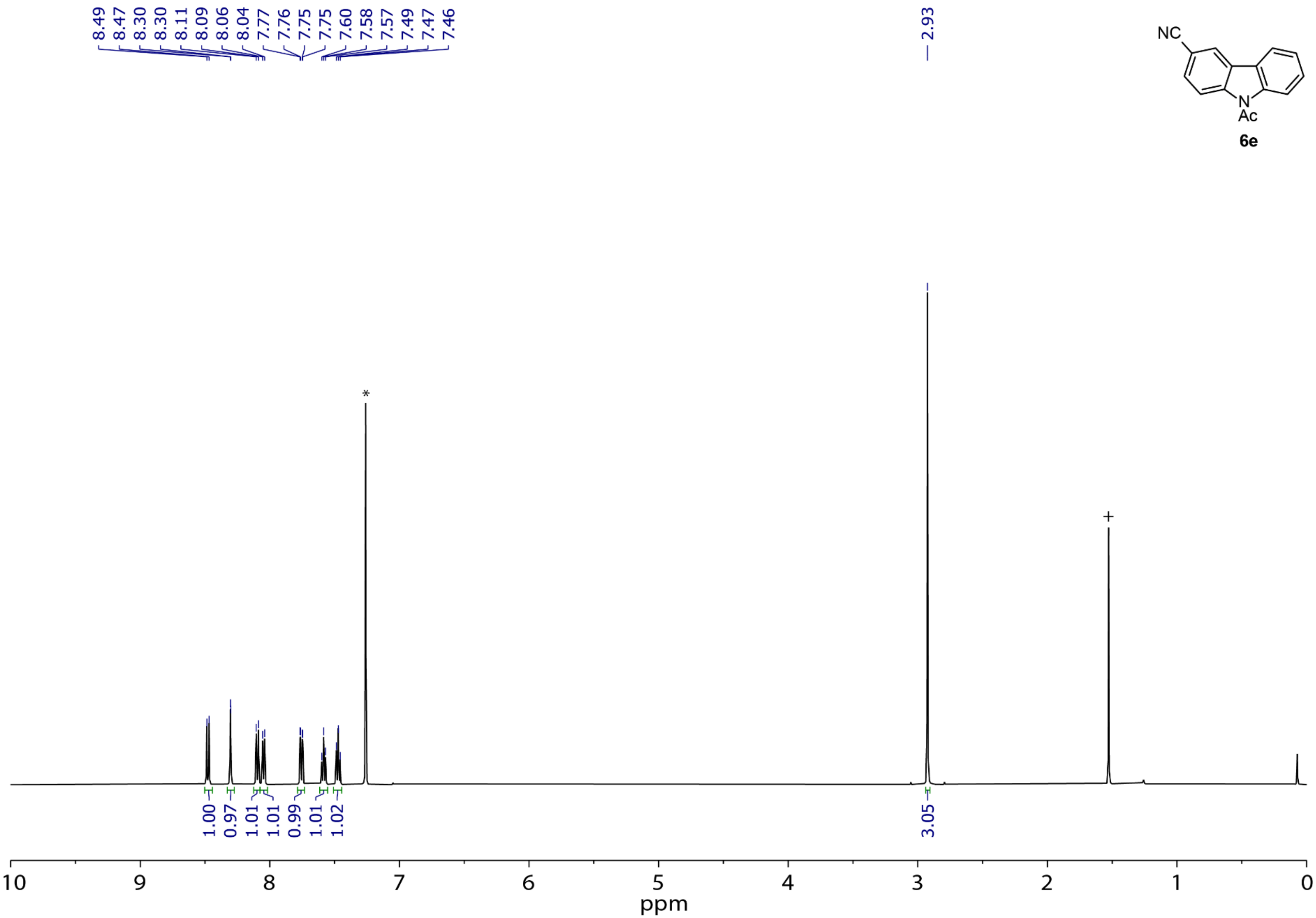

6e

Figure S41. ^{1}H NMR spectrum of 9-acetyl-9H-carbazole-3-carbonitrile (**6e**) in CD_3Cl_3 (400 MHz) at 23 °C. Solvent peak is marked with *. Water peak is marked with $^{+}$.

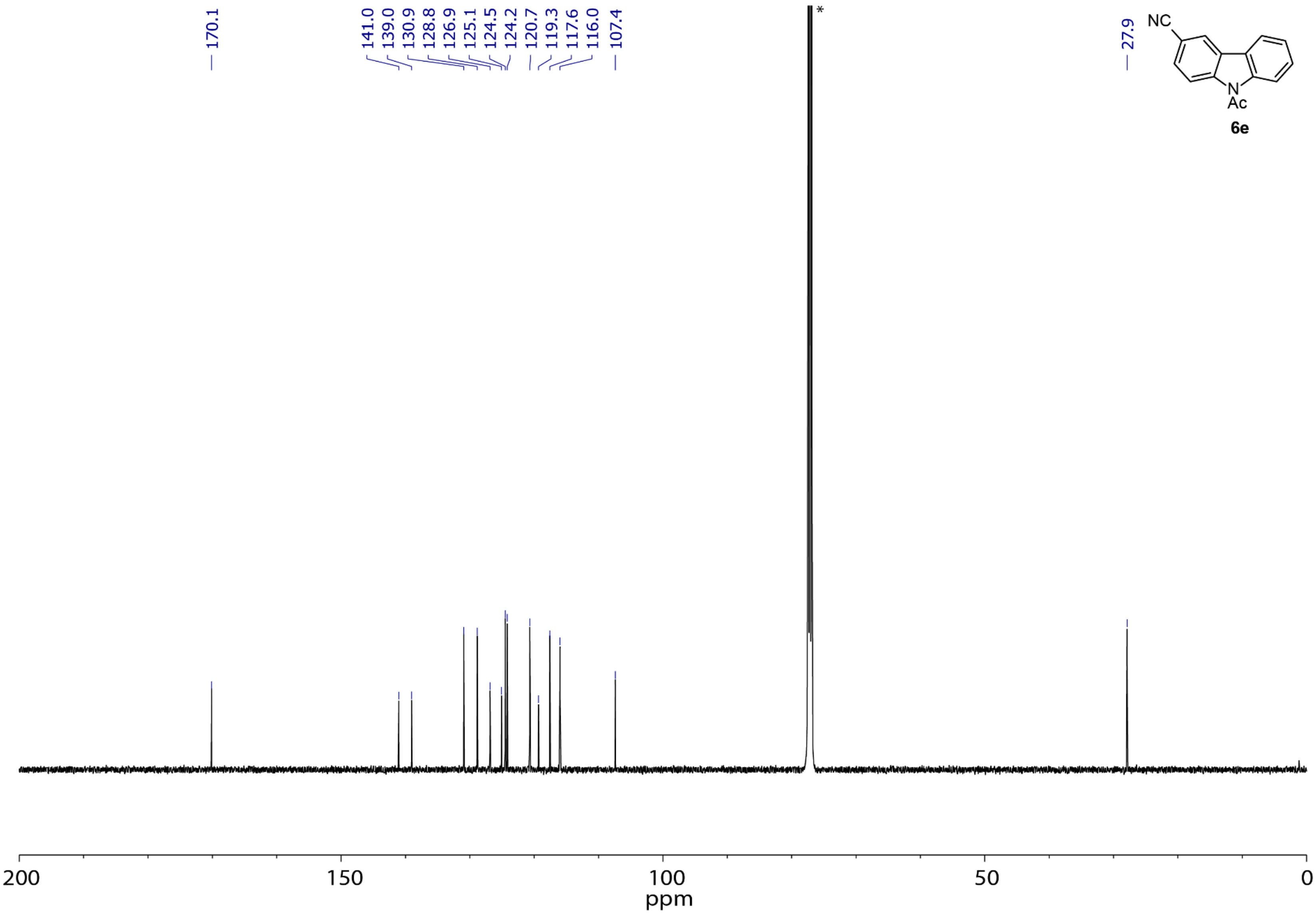

Figure S42. ^{13}C NMR spectrum of 9-acetyl-9H-carbazole-3-carbonitrile (**6e**) in CD_3Cl_3 (400 MHz) at 23 °C. Solvent peak is marked with *.

H. References

1. Motterlini, R. A.; Mann, B. E.; Scapens, D. A. Therapeutic Delivery of Carbon Monoxide. *U.S. Patent US 2010105770*, April 28, 2010.
2. Toro, J. M. S.; den Hartog, T.; Chen, P. Cyclopropanation of Styrenes and Stilbenes Using Lithiomethyl Trimethylammonium Triflate as Methylene Donor. *Chem. Commun.* **2014**, *50*, 10608–10610.
3. Frey, B. L.; Figgins, M. T.; Van Trieste, G. P., III; Carmieli, R.; Powers, D. C. Iodine–Iodine Cooperation Enables Metal-Free C–N Bond-Forming Electrocatalysis via Isolable Iodanyl Radicals. *J. Am. Chem. Soc.* **2022**, *144*, 13913–13919.
4. Yoshida, S.; Shimomori, K.; Kim, Y.; Hosoya, T. Single C−F Bond Cleavage of Trifluoromethylarenes with an ortho-Silyl Group. *Angew. Chem. Int. Ed.* **2016**, *55*, 10406–10409.
5. Brosse, N.; Pinto, M. F.; Jamart-Grégoire, B. Preparation of Multiply Protected Alkylhydrazine Derivatives by Mitsunobu and PTC Approaches. *Eur. J. Org. Chem.* **2003**, *2003*, 4757–4764.
6. Hyun, S.-M.; Yuan, M.; Maity, A.; Gutierrez, O.; Powers, D. C. The Role of Iodanyl Radicals as Critical Chain Carriers in Aerobic Hypervalent Iodine Chemistry. *Chem* **2019**, *5*, 2388–2404.
7. Rose, I.; Bezzu, C. G.; Carta, M.; Comesaña-Gándara, B.; Lasseuguette, E.; Ferrari, M. C.; Bernardo, P.; Clarizia, G.; Fuoco, A.; Jansen, J. C.; et al. Polymer Ultrapermeability from the Inefficient Packing of 2D Chains. *Nature Mater.* **2017**, *16*, 932–937.
8. Maity, A.; Frey, B. L.; Hoskinson, N. D.; Powers, D. C. Electrocatalytic C–N Coupling via Anodically Generated Hypervalent Iodine Intermediates. *J. Am. Chem. Soc.* **2020**, *142*, 4990–4995.
9. Fulmer, G. R.; Miller, A. J.; Sherden, N. H.; Gottlieb, H. E.; Nudelman, A.; Stoltz, B. M.; Bercaw, J. E.; Goldberg, K. I. NMR Chemical Shifts of Trace Impurities: Common Laboratory Solvents, Organics, and Gases in Deuterated Solvents Relevant to the Organometallic Chemist. *Organometallics* **2010**, *29*, 2176–2179.
10. Frisch, M. J.; Trucks, G. W.; Schlegel, H. B.; Scuseria, G. E.; Robb, M. A.; Cheeseman, J. R.; Scalmani, G.; Barone, V.; Petersson, G. A.; Nakatsuji, H.; Li, X.; Caricato, M.; Marenich, A. V.; Bloino, J.; Janesko, B. G.; Gomperts, R.; Mennucci, B.; Hratchian, H. P.; Ortiz, J. V.; Izmaylov, A. F.; Sonnenberg, J. L.; Williams-Young, D.; Ding, F.; Lipparini, F.; Egidi, F.; Goings, J.; Peng, B.; Petrone, A.; Henderson, T.; Ranasinghe, D.; Zakrzewski, V. G.; Gao, J.; Rega, N.; Zheng, G.; Liang, W.; Hada, M.; Ehara, M.; Toyota, K.; Fukuda, R.; Hasegawa, J.; Ishida, M.; Nakajima, T.; Honda, Y.; Kitao, O.; Nakai, H.; Vreven, T.; Throssell, K.; Montgomery, J. A., Jr.; Peralta, J. E.; Ogliaro, F.; Bearpark, M. J.; Heyd, J. J.; Brothers, E. N.; Kudin, K. N.; Staroverov, V. N.; Keith, T. A.; Kobayashi, R.; Normand, J.; Raghavachari, K.; Rendell, A. P.; Burant, J. C.; Iyengar, S. S.; Tomasi, J.; Cossi, M.; Millam, J. M.; Klene, M.; Adamo, C.; Cammi,

R.; Ochterski, J. W.; Martin, R. L.; Morokuma, K.; Farkas, O.; Foresman, J. B.; Fox, D. J. *Gaussian 16 Rev. C. 01*; Wallingford, CT, 2016.

11. Lee, C.; Yang, W.; Parr, R. G. Development of the Colle-Salvetti Correlation-Energy Formula into a Functional of the Electron Density. *Phys. Rev. B* **1988**, *37*, 785.
12. Becke, A. D. Density-Functional Thermochemistry. I. The Effect of the Exchange-Only Gradient Correction. *J. Chem. Phys.* **1992**, *96*, 2155–2160.
13. Grimme, S. Accurate Description of Van der Waals Complexes by Density Functional Theory Including Empirical Corrections. *J. Comput. Chem.* **2004**, *25*, 1463–1473.
14. Grimme, S.; Antony, J.; Ehrlich, S.; Krieg, H. A Consistent and Accurate Ab Initio Parametrization of Density Functional Dispersion Correction (DFT-D) for the 94 Elements H-Pu. *J. Chem. Phys.* **2010**, *132*, 154104.
15. Grimme, S. Density Functional Theory with London Dispersion Corrections. *Wiley Interdiscip. Rev.: Comput. Mol. Sci.* **2011**, *1*, 211–228.
16. Ehrlich, S.; Moellmann, J.; Grimme, S. Dispersion-Corrected Density Functional Theory for Aromatic Interactions in Complex Systems. *Acc. Chem. Res.* **2013**, *46*, 916–926.
17. Godbout, N.; Salahub, D. R.; Andzelm, J.; Wimmer, E. Optimization of Gaussian-Type Basis Sets for Local Spin Density Functional Calculations. Part I. Boron through Neon, Optimization Technique and Validation. *Can. J. Chem.* **1992**, *70*, 560–571.
18. Klamt, A.; Schüürmann, G. COSMO: A New Approach to Dielectric Screening in Solvents with Explicit Expressions for the Screening Energy and Its Gradient. *J. Chem. Soc., Perkin Trans. 2* **1993**, 799–805.
19. Tomasi, J.; Persico, M. Molecular Interactions in Solution: An Overview of Methods Based on Continuous Distributions of the Solvent. *Chem. Rev.* **1994**, *94*, 2027–2094.
20. Andzelm, J.; Kölmel, C.; Klamt, A. Incorporation of Solvent Effects into Density Functional Calculations of Molecular Energies and Geometries. *J. Chem. Phys.* **1995**, *103*, 9312–9320.
21. Barone, V.; Cossi, M. Quantum Calculation of Molecular Energies and Energy Gradients in Solution by a Conductor Solvent Model. *J. Phys. Chem. A* **1998**, *102*, 1995–2001.
22. Marenich, A. V.; Cramer, C. J.; Truhlar, D. G. Universal Solvation Model Based on Solute Electron Density and on a Continuum Model of the Solvent Defined by the Bulk Dielectric Constant and Atomic Surface Tensions. *J. Phys. Chem. B* **2009**, *113*, 6378–6396.
23. Gonzalez, C.; Schlegel, H. B. Reaction Path Following in Mass-Weighted Internal Coordinates. *J. Phys. Chem.* **1990**, *94*, 5523–5527.
24. Deng, L.; Ziegler, T.; Fan, L. A Combined Density Functional and Intrinsic Reaction Coordinate Study on the Ground State Energy Surface of H_2CO. *J. Chem. Phys.* **1993**, *99*, 3823–3835.
25. Deng, L.; Ziegler, T. The Determination of Intrinsic Reaction Coordinates by Density Functional Theory. *Int. J. Quantum Chem.* **1994**, *52*, 731–765.
26. Johnson, E. R.; Keinan, S.; Mori-Sánchez, P.; Contreras-García, J.; Cohen, A. J.; Yang, W. Revealing Noncovalent Interactions. *J. Am. Chem. Soc.* **2010**, *132*, 6498–6506.

27. Contreras-García, J.; Johnson, E. R.; Keinan, S.; Chaudret, R.; Piquemal, J.-P.; Beratan, D. N.; Yang, W. NCIPLOT: A Program for Plotting Noncovalent Interaction Regions. *J. Chem. Theory Comput.* **2011**, *7*, 625–632
28. Chinnagolla, R. K.; Jeganmohan, M. Ruthenium-Catalyzed Ortho-Arylation of Acetanilides with Aromatic Boronic Acids: An Easy Route to Prepare Phenanthridines and Carbazoles. *Chem. Commun.* **2014**, *50*, 2442–2444.
29. Tsang, W. C. P.; Munday, R. H.; Brasche, G.; Zheng, N.; Buchwald, S. L. Palladium-Catalyzed Method for the Synthesis of Carbazoles via Tandem C−H Functionalization and C−N Bond Formation. *J. Org. Chem.* **2008**, *73*, 7603–7610.
30. Tsang, W. C. P.; Zheng, N.; Buchwald, S. L. Combined C−H Functionalization/C−N Bond Formation Route to Carbazoles. *J. Am. Chem. Soc.* **2005**, *127*, 14560–14561.
31. Antonchick, A. P.; Samanta, R.; Kulikov, K.; Lategahn, J. Organocatalytic, Oxidative, Intramolecular C–H Bond Amination and Metal-Free Cross-Amination of Unactivated Arenes at Ambient Temperature. *Angew. Chem. Int. Ed.* **2011**, *50*, 8605–8608.
32. Chng, L. L.; Yang, J.; Wei, Y.; Ying, J. Y. Palladium Nanomaterials in Catalytic Intramolecular C–H Amination Reactions. *Chem. Commun.* **2014**, *50*, 9049–9052.
33. Cho, S. H.; Yoon, J.; Chang, S. Intramolecular Oxidative C−N Bond Formation for the Synthesis of Carbazoles: Comparison of Reactivity Between the Copper-Catalyzed and Metal-Free Conditions. *J. Am. Chem. Soc.* **2011**, *133*, 5996–6005.
34. Wang, S.; Mao, H.; Ni, Z.; Pan, Y. Pd(II)-Catalyzed Intramolecular C–H Activation/C–C Cross Coupling for the Synthesis of Carbazoles from Diaryl Acetamides. *Tetrahedron Lett.* **2012**, *53*, 505–508.
35. Maity, A.; Frey, B. L.; Hoskinson, N. D.; Powers, D. C. Electrocatalytic C–N Coupling via Anodically Generated Hypervalent Iodine Intermediates. *J. Am. Chem. Soc.* **2020**, *142*, 4990–4995.
36. Thai, P.; Frey, B. L.; Figgins, M. T.; Thompson, R. R.; Carmieli, R.; Powers, D. C. Selective Multi-Electron Aggregation at a Hypervalent Iodine Center by Sequential Disproportionation. *Chem. Commun.* **2023**, *59*, 4308–4311.
